# Tracking Ideal Varieties and Cropping Techniques for Agroecological Weed Management: A Simulation-Based Study on Pea

**DOI:** 10.3389/fpls.2022.809056

**Published:** 2022-04-04

**Authors:** Nathalie Colbach, Emeline Felten, Christelle Gée, Antony Klein, Laura Lannuzel, Christophe Lecomte, Thibault Maillot, Florence Strbik, Jean Villerd, Delphine Moreau

**Affiliations:** Agroécologie, INRAE, Institut Agro, Univ. Bourgogne, Univ. Bourgogne Franche-Comté, F-21000, Dijon, France

**Keywords:** pea (*Pisum sativum*), weed damage, trait, yield loss, yield gap, ideotype, multi-criteria decision, trade-off

## Abstract

Pea or *Pisum sativum* L. is a key diversification crop, but current varieties are not very competitive against weeds. The objective was to identify, depending on the type of cropping system and weed flora, (1) the key pea parameters that drive crop production, weed control and weed contribution to biodiversity, (2) optimal combinations of pea-parameter values and crop-management techniques to maximize these goals. For this, virtual experiments were run, using FLORSYS, a mechanistic simulation model. This individual-based 3D model simulates daily crop-weed seed and plant dynamics over the years, from the cropping system and pedoclimate. Here, this model was parameterized for seven pea varieties, from experiments and literature. Moreover, ten virtual varieties were created by randomly combining variety-parameter values according to a Latin Hypercube Sampling (LHS) plan, respecting parameter ranges and correlations observed in the actual varieties. A global sensitivity analysis was run, using another LHS plan to combine pea varieties, crop rotations and management techniques in nine contrasting situations (e.g., conventional vs. organic, no-till, type of weed flora). Simulated data were analyzed with classification and regression trees (CART). We highlighted (1) Parameters that drive potential yield and competitivity against weeds (notably the ability to increase plant height and leaf area in shaded situations), depending on variety type (spring vs. winter) and cropping system. These are pointers for breeding varieties to regulate weeds by biological interactions; (2) Rules to guide farmers to choose the best pea variety, depending on the production goal and the cropping system; (3) The trade-off between increasing yield potential and minimizing yield losses due to weeds when choosing pea variety and management, especially in winter peas. The main pea-variety rules were the same for all performance goals, management strategies, and analyses scales, but further rules were useful for individual goals, strategies, and scales. Some variety features only fitted to particular systems (e.g., delayed pea emergence is only beneficial in case of herbicide-spraying and disastrous in unsprayed systems). Fewer variety rules should be compensated by more management rules. If one of the two main weed-control levers, herbicide or tillage, was eliminated, further pea-variety and/or management rules were needed.

## Introduction

Today, pea (*Pisum sativum* L.) is a minor crop in temperate arable cropping systems (e.g., for France^1^). However, integrating pea into rotations potentially has many benefits. As a legume crop, it allows reducing nitrogen fertilizer use (Nemecek et al., 2008) and diversifying cereal-based rotations to control weed infestations (Chauvel et al., 2001) or pests (Ratnadass et al., 2012). Pea also provides proteins for both human and animal food supply (Voisin et al., 2014; Duc et al., 2015; Watson et al., 2017).

Pea reduces weed infestation at the rotation scale by diversifying weed control options and disrupting the growing season of the more harmfula utumn-emerging weeds (Chauvel et al., 2001). These effects are essential in the necessary shift from herbicide-based to agroecological weed management (Weisberger et al., 2019). However, the current pea varieties are not very competitive against weeds, notably because of a slow crop establishment (McDonald, 2003). Pea is known to be genetically diverse, in terms of flowering precocity, soil cover, plant height, and architecture (Espósito et al., 2007; Vocanson and Jeuffroy, 2008; Burstin et al., 2015; Spies et al., 2017), and collections of pea lines are available (Smýkal et al., 2012; Tayeh et al., 2015; Siol et al., 2017). Potentially, more competitive varieties could be produced. But, though the traits that make a crop competitive against weeds are known in general (Colbach et al., 2019) and, particularly for cereals (maize, Ford and Pleasant, 1994; barley, Christensen, 1995; Mennan and Zandstra, 2005; wheat, Drews et al., 2009; rice, Mahajan et al., 2014), they are not yet known specifically for pea. These studies highlighted the key role of shade response, which is not among the traits routinely monitored during crop selection.

Screening existing pea lines in experiments is not only time-consuming but presents methodological drawbacks when it comes to assessing weed harmfulness. It is notoriously difficult to correctly estimate crop yield loss due to weeds because it is next to impossible to ensure a continuously weed-free control to measure potential yield (Colbach et al., 2020c). Many studies thus use weed biomass (or worse, weed densities) as a proxy for yield loss (see references in Colbach et al., 2020c) but the results are often difficult to extrapolate to other situations. Indeed, competitive crop traits were shown to vary in terms of impact between years, because of weather or management effects (see references in Andrew et al., 2015).

Working with mechanistic (process-based) models makes it easier to identify limiting factors and relevant species traits. Consequently, many studies now rely on simulation to assess crop genotypes or to identify ideotypes (i.e., theoretical ideal crop plants that combine traits to optimize crop performance in a particular environment, crop management and end-use, Martre et al., 2015), whether for, e.g., tolerance to climate change (Tao et al., 2017), fruit quality (Memmah et al., 2014) or for weed management (Bastiaans et al., 1997; Colbach et al., 2019). One of the rare pea studies (Jeuffroy et al., 2012) compared contrasting pea varieties (including virtual ones based on expert knowledge), evaluating their performance in response to soil compaction in interaction with tillage/sowing dates and weather. However, their objective was to assess the robustness of genotypes and management strategies relative to weather repetitions rather than to identify key parameters or optimal parameter-value combinations.

The quality of these various assessments is highly dependent on the model type and its prediction quality. When focusing on pea competitivity against weeds, we need models that produce realistic predictions of crop-weed interactions in a large range of cropping systems, pedoclimates, and weed floras. This means an individual-based model to account for the temporal and morphological plasticity in weeds (Renton, 2013; Colbach et al., 2021). It should be parameterized for many crop and weed species to account for the diverse weed floras in arable crops (Fried et al., 2008). It must include all crop management techniques to account for both direct and indirect (e.g., via impacts on the environment) effects on weeds (Colbach and Debaeke, 1998; Colbach et al., 2021), and this over several years or decades to account for weed seed persistence in the soil (Lewis, 1973). To date, FLORSYS (Colbach et al., 2021) is the model that best meets these needs.

The objective of the present study was to identify parameters that make pea competitive against weeds, depending on management strategies. To achieve this, we (1) parameterized the FLORSYS model with contrasting pea varieties from experiments and literature, (2) created virtual varieties based on these data, (3) ran virtual experiments with these actual and virtual varieties in contrasting cropping systems to identify the pea parameters that drive crop production, weed disservices and services (hence (dis)services), (4) identified optimal pea-parameter value combinations, depending on the production goal and the cropping-system type. Production goals included not only yield, but also reduced herbicide use and weed contribution to biodiversity.

## Materials and Methods

### The Virtual Field FLORSYS

FLORSYS is a virtual field on which cropping systems can be experimented with a large range of virtual measurements of crop, weed, and environmental state variables (Gardarin et al., 2012; Munier-Jolain et al., 2013, 2014; Colbach et al., 2014a,b, 2021; Pointurier et al., 2021).

#### Weed and Crop Life Cycle

The input variables of FLORSYS consist of (1) a description of the simulated field (daily weather, latitude, and soil characteristics); (2) all the crops and management operations in the field, with dates, tools, and options; and (3) the initial weed seed bank. These input variables influence the annual life cycle of annual weeds and crops, with a daily time-step.

Pre-emergence stages (surviving, dormant, and germinating seeds, emerging seedlings) are driven by seed depth, soil structure, temperature, and water potential. The crop-weed canopy is represented in 3D, with each crop and weed plant schematized as a cylinder (above ground) and on top of a spilled cone (below ground). Post-emergence processes (e.g., photosynthesis, respiration, growth, shade response) are driven by light availability and air temperature. At plant maturity, weed seeds are added to the soil seed bank; crop seeds are harvested to determine crop yield. Nitrogen stress is disregarded in the present model version, and water stress is only considered for pre-emergent processes.

The model is currently parameterized for 26 frequent and contrasting annual weed species and 32 crop species (section A.2 of the Supplementary Material).

#### Effect of Cultural Techniques

Life-cycle processes depend on the dates, options, and tools of management techniques (tillage, sowing, herbicides, mechanical weeding, mowing, harvesting), in interaction with weather and soil conditions on the day the operations are carried out (section A.3 of the Supplementary Material).

#### Indicators of Weed Impact on Crop Production and Biodiversity

FLORSYS simulates crop yield as well as indicators assessing weed impacts on crop production and biodiversity (Mézière et al., 2015; Colbach et al., 2020a) (see section A.4 of the Supplementary Material). Here, one yield indicator was considered, i.e., potential crop yield from weed-free simulations. Then, two indicators of weed harmfulness for crop production were analyzed: crop yield loss, as the relative crop yield difference of simulations with and without weeds, as well as field infestation. In addition, two indicators of weed contribution to biodiversity were included, i.e., weed species richness (illustrating wild plant biodiversity) and weed contribution to feeding domestic bees (as an example for functional biodiversity).

#### Domain of Validity

FLORSYS was evaluated with independent field data (Colbach et al., 2016; Pointurier et al., 2021). This showed that crop yields, daily weed species densities, and densities averaged over the years were generally well predicted and ranked as long as a corrective function was added to keep weeds from flowering during winter at more southern latitudes. Higher crop yield losses than those reported in previous field studies resulted from the simulation plan. This does not adapt practices to simulated weed floras and interannual weather variability (as farmers or trial managers would do), in order to discriminate the effect of crop species and management practices on weeds from the effect of weeds on the choice of crops and practices (Colbach and Cordeau, 2018).

### Parameterize Contrasting Pea Varieties

#### Choosing Contrasting Pea Varieties and Necessary Experiments

**FIGURE 1:**
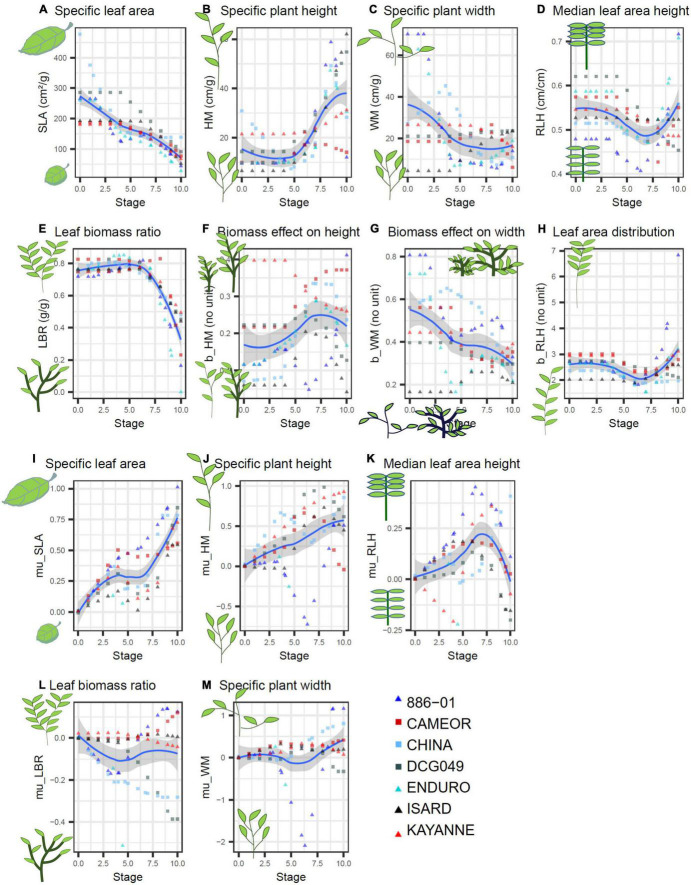
Parameters describing potential plant morphology in unshaded conditions **(A–H)** and shading response **(I–M)** measured on 7 pea varieties (red symbols = spring varieties, blueish colors = winter varieties, ▲ = afila, ■ = Leafy) over plant stages (BBCH) estimated from garden-plot experiments. SLA, plant leaf area/plant leaf biomass; LBR, plant leaf biomass/plant above-ground biomass; HM, plant height/plant above-ground biomass^b_HM^; WM, plant width/plant above-ground biomass^b_WM^; RLH, plant height below which 50% of leaf area are located/total plant height; b_RLH, shape parameter for leaf area distribution along plant height. mu_X shapes shade response of morphogical variable X (SLA, LBR, etc.) *via* X = X_unshaded_ ⋅ exp(-mu_X ⋅ shading intensity) (Colbach, 2020 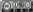).

For this study, seven pea varieties contrasting in terms of seasonality and morphology were chosen (Table 1A) and parameterized for FLORSYS. In FLORSYS, a crop species is represented by 220 parameters (section A.4 of the Supplementary Material). To parameterize pea varieties, we used existing literature (Lecomte et al., 2003; Raveneau et al., 2011; Tayeh et al., 2015; Castel et al., 2017; Varela Nicola, 2017; Colbach et al., 2020b), extracted parameters from other models (Brisson et al., 2009; Pointurier et al., 2021) and set up experiments when no parameter source was available. This was mainly the case for parameters describing potential plant morphology in unshaded conditions and response to shading by neighbor plants. These key processes drive crop-weed competition and determine how fast plants occupy space once they emerge, how much space they occupy, and how they adapt light capture when surrounded by neighbor plants. In FLORSYS, potential plant morphology in unshaded conditions depends on eight parameters per species and stage, for 11 plant stages (Figures 1A–H). Two further parameters determine maximum plant height and width in unlimiting growth conditions. These parameters determine plant dimensions, leaf area, and leaf-area distribution along plant height in unshaded conditions. Another five parameters per species and stage drive species response to shading, determining whether shaded plants invest more into plant height vs. width or into leaf vs. stem biomass, whether they reduce their leaf thickness to increase leaf area, and whether they shift their leaves upward or downward (Figures 1I–M).

**TABLE 1:** List of pea (*Pisum sativum* L.) varieties tested in the present experiments.

A. A few species parameters measured in garden plot experiments and field experiments.									
Variety	Seasonality^&^	Leaf morphology^#^	Usage	Seed mass (mg)^§^		Harvest index^☼^ (g/g)		Plant dimensions in unlimited growth conditions^$^	
								**Height (cm)**	**Width (cm)**
Cameor	Spring	Leafy	Garden	157		0.45		75	72
Kayanne	Spring	afila	Protein	183		0.52		121	89
China	Winter Hr	Leafy	Forage	153		0.35		165	100
DCG0449	Winter Hr	Leafy	Forage	102		0.30		160	105
886/1	Winter Hr	afila	Protein	131		0.40		145	120
Enduro	Winter hr	afila	Protein	187		0.52		110	70
Isard	Winter hr	afila	Protein	153		0.54		97	63

**B. A few indicators of pea production and weed impacts simulated by FLORSYS. Lsmeans (and SE) were calculated after analyses of variance of indicator as a function of pea variety, situation, repetition, simulation year, and interactions (not significantly different at *p* = 0.05 if followed by the same letter, based on least significant difference test). *SD*s are standard deviations per variety. Cells of each column were colored from green = best (highest yield, lowest yield loss or field infestation, lowest standard-error) to red = worst (vice-versa).**									

**Variety**	**Potential yield (t/ha)**			**Yield loss (%)**			**Field infestation (t/ha)**		

Cameor	4.51	(1.35)	d	48.6	(37.5)	d	2.73	(2.89)	a
Kayanne	5.18	(1.26)	b	33.7	(36.5)	e	2.01	(2.29)	e
China	2.65	(0.97)	g	58.9	(33.2)	b	2.19	(1.65)	d
DCG0449	5.07	(1.03)	c	55.1	(34.5)	c	2.41	(2.03)	c
886/1	3.98	(0.96)	e	56.3	(33.2)	c	2.56	(2.11)	b
Enduro	3.14	(1.77)	f	74.1	(27.1)	a	2.57	(1.91)	b
Isard	6.67	(1.15)	a	57.1	(32.5)	bc	2.29	(1.79)	cd

*^&^Reactive (dominant Hr) or non-reactive (recessive hr) to photoperiod type. ^#^afila (recessive af) or Leafy (dominant Af) type. ^§^Dry mass per seed. ^☼^The ratio of dry seed biomass/dry above-ground plant biomass. ^$^Without shading and with unlimited soil resources.*

#### Garden Plot Experiments

The principle for estimating parameters of potential morphology and shading response in garden plots was described by Colbach et al. (2020b), based on the experimental and computational approaches developed by Munier-Jolain et al. (2014). In summary, isolated plants were sown in garden plots at the usual crop sowing dates, either under shading nets to mimic shading by neighbor plants or in unshaded conditions. The plants were placed at a distance exceeding 50 cm to avoid any interference. With each sampling date, plant-plant distance increased as more plants were taken out for measurements. Temperature and incident photosynthetically radiation (PAR) were measured continuously from sowing up to harvest inside and outside the shading cage. The plots were fertilized, watered, and hand-weeded regularly to avoid any water or nitrogen stress. Experiments were carried out in Dijon Burgundy, France (47°19′2.624″N, 5°4′26.883″E, 257 m asl), in 2010 (varieties 886/1, China, Enduro) and 2018 (varieties Cameor, DCG0449, Isard, Kayanne). The soil was 0.33 g/g clay, 0.49 g/g silt, and 0.17 g/g sand, with pH = 8.3 and 0.31 g organic matter/g soil.

For each variety, four to eight plants were sampled at five stages, ranging from the 2-leaf stage to the end of flowering. A vertical RGB image of each sampled plant was taken with a Canon powerShot SX20 IS (Canon Inc., Tokyo, Japan) commercial digital camera. For each image, a white calibration square (10 cm x 10 cm) was placed next to the plant to determine the spatial resolution of the camera. Vegetation was discriminated from the background using the excess green vegetation index (ExG) developed by Woebbecke et al. (1995). Then, binary thresholding (B&W) was performed using Otsu’s method (Otsu, 1979; Gée et al., 2021). Finally, the distribution of leaf area vs. relative plant height was determined. All the image-processing algorithms were implemented in Matlab (Version 2016b, The Mathworks, Natick, MA, United States).

Then, plant height and width, leaf area and biomass as well as total above-ground biomass were measured. For the latter two, leaves (including petioles), stems, and reproductive parts were discriminated. At each sampling stage and for each variety, these measurements were used to determine eight parameters (Figures 1A–H) characterizing potential plant morphology by fitting key state variables (e.g., plant height or width) against above-ground biomass using the plants grown in unshaded conditions. Then, five shade-response parameters (Figures 1I–M) were estimated by fitting the main morphology variables (e.g., specific leaf area SLA) against shading intensity (i.e., 1 – PAR in shaded/PAR in unshaded conditions) with a non-linear regression using all (shaded and unshaded) plants sampled at the same stage. Finally, for each variety, the parameter values from the five sampling stages were extrapolated to obtain parameter values for 11 BBCH stages ranging from emergence (stage 0) to full maturity (stage 10), using non-parametric regressions. All statistical analyses were carried out, using SAS (PROC REG, NLIN, and LOESS). Further details can be found in section B of the Supplementary Material.

### Virtual Experiments

#### Create Virtual Pea Varieties

To determine correlations among pea parameters, Pearson correlations coefficients were calculated among the 220 parameters of the seven parameterized pea varieties, using corr() of Hmisc package of R (R Core Team, 2016). In addition, the minimum and maximum values for each quantitative parameter were determined for pea (section D.3 of the Supplementary Material). This range of variation was extended to [0.9 × min, 1.1 × max] for each parameter, but capped to remain biologically realistic (e.g., a proportion such as a harvest index needed to be in [0, 1] g/g).

Furthermore, ten virtual pea varieties were created by randomly drawing parameter values inside the extended range of variation of the seven actual varieties following a Latin Hypercube Sampling (LHS) plan and using the correlation matrix as constraints. This was done using the randomLHS() and LHScorcorr() functions of the lhs package of R.

#### Simulation Plan

The simulation plan aimed to evaluate the impact of pea parameters and management techniques in contrasting cropping systems, with a global sensitivity analysis (i.e., varying factors simultaneously to analyze the sensitivity to factors and their interactions, Saltelli et al., 2000). First, nine situations were chosen, varying in terms of rotation length and diversity, management techniques, and initial weed seed bank (Table 2). The two contrasting weed seed banks were based on a preliminary simulation study linking weed species to weed impact (section D.1 of the Supplementary Material).

**TABLE 2:** Contrasting situations used to stratify the simulation plan testing the sensitivity of crop production and weed impact to pea parameters and management techniques with FLORSYS simulations. The two alternative weed seed banks were based on a preliminary simulation study linking weed species to weed impact (section D.1 of the Supplementary Material). For each situation, 400 cropping systems were built by randomly choosing management techniques based on a Latin Hypercube Sampling (LHS) plan and respecting the constraints of the situation. Pea varieties were chosen among a pool of 18 (virtual and actual) varieties.

Situation	Possible crops in the rotation (in any order, each crop only once)	Possible management techniques				Weed species pool
		Tillage	Herbicides	Mechanical weeding	Fertilizer^§^		

Reference	Pea, WW, WOSR	Yes	Yes	No	Mineral	Complete (26 species)

Change management strategy						
Complete		Yes	Yes	Yes	Mineral	Same as reference
Organic	Same as reference	Yes	No	Yes	Organic		
No till		No	Yes	No	Mineral	

**Change crop rotation**						

2-year rotation	Pea, WW	Same as reference				Same as reference
4-year rotation	Pea, WW, WOSR, B					
6-year rotation	Pea, WW, WOSR, B, S, M					

**Change initial weed seed bank^$^**						

Harmful	Same as reference	Same as reference				6 harmful weeds
Harmful + bee food						6 harmful dicots promoting bee food

*WW, winter wheat; WOSR, winter oilseed rape; B, winter or spring barley; S, sunflower; M, maize. ^§^Though competition for nitrogen is disregarded as simulations are run with unlimited nitrogen conditions, organic fertilizer influences weed seed germination/emergence by adding a layer of organic matter on the soil surface. ^$^The two alternative seed banks were based on a preliminary simulation study linking weed species to weed impact (section D.1 of the Supplementary Material).*

For each situation, 400 cropping systems were built by randomly choosing management techniques based on a Latin Hypercube Sampling (LHS) plan and respecting the constraints of Table 2. For pea, varieties were chosen among a pool of 18 varieties: the seven actual varieties of Table 1, the ten virtual ones of section “Create Virtual Pea Varieties,” and a final one whose parameters were estimated from simulations with the STICS crop model (Brisson et al., 2003). Dates and options of techniques (e.g., depth, speed, and tool for tillage) were randomly chosen for each crop but respecting agronomic logic (e.g., winter wheat could not be sown in spring, sowing and harvesting dates of pea varieties allowed full maturation, no-tillage after crop sowing). The analysis of the Pearson correlations among simulation factors showed that our simulation plan was adequate to avoid confusing effects among management techniques. Indeed, the median correlation coefficient among crop management techniques as well as between techniques and rotation or situation varied from 0.05 to 0.09, depending on variety types and crops (details in section D.2.1 of the Supplementary Material). Pea parameters and management techniques were even less correlated (0.03–0.06). The Latin Hypercube Sampling (LHS) plan thus allowed decorrelating management variables. Correlations among rotation variables or between rotation and situation were much higher (0.12–0.21), reflecting the rotation rules fixed for each situation. Pea parameters were highly correlated (0.23–0.3), reflecting the biological correlations among variety characteristics, which we introduced by constraining the LHS via parameter correlations.

The 9 × 400 cropping systems were simulated over 12 consecutive years to assess long-term effects and repeated five times with five weather series consisting of randomly chosen records from the INRAE Dijon weather station (INRAE Climatik database). Each system × repetition was run twice, once starting with weeds and once without, to assess potential crop yield. Crop yield loss due to weeds was then calculated as (yield from weed-free simulation – yield from weedy simulation)/(yield from weed-free simulation). This simulation plan succeeded in producing differences in pea and weed (dis)services across situations (see details in section D.6 of the Supplementary Material).

#### Statistics

To make the indicators of potential crop yield and weed (dis)services (see section “Indicators of Weed Impact on Crop Production and Biodiversity”) comparable, they were rescaled to [0,1] with 0 corresponding to the worst value (lowest yield or biodiversity, highest harmfulness) across all situations, cropping systems, years and weather repetitions, and 1 to the best (highest yield or biodiversity, lowest harmfulness). To stress that a rescaled weed-harmfulness value of 1 means low harmfulness (e.g., low yield loss), we renamed such indicators as “weed-harmfulness control” (e.g., yield-loss control).

To analyze trade-offs among indicators of yield and weed (dis)services, a PCA was run across all situations with the PCA() function of R software version 4.0.1 (R Core Team, 2021), separately for cropping systems with spring vs. winter varieties.

**FIGURE 2:**
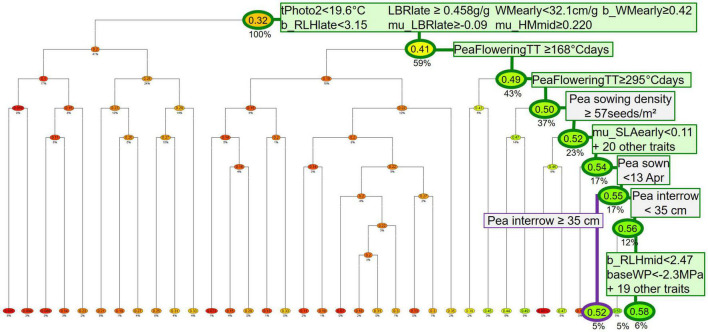
Example of regression and classification tree (CART) analyzing potential yield of spring pea from weed-free simulations (response variable) as a function of pea parameters (in green), pea management techniques, and other-crop techniques (in gray) (predictors). Only pea crops with herbicides were used here. The highlighted branches show the best and third-best performances (which are also shown in Table 5A). Boxes show principal and surrogate predictors and thresholds for splitting branches (only for the two highlighted branches). Leaves (at the bottom of the tree) and nodes show indicator values normed to [0 = lowest yield, 1 = highest yield of simulated data set]; leaves are colored from red (lowest yield) through yellow (intermeidate yield) to green (highest yield). Percentages below nodes show the proportion of individuals that fall into that node. The root node at the top of the tree has 100% since the dataset has not been split yet (Colbach, 2021 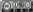).

To identify which simulation factors (situation, pea parameters, management techniques for peas and other crops, weather repetition) influence the indicators, classification and regression trees (CART) (Breiman et al., 1984) were run per pea variety type (spring or winter), either on annual data (years with a pea) or data averaged over the simulation, including years grown with crops other than a pea (rotation scale). The trees predict a continuous response variable (here either individual indicators, or a combination of several indicators hence called “performance profile”) from a set of discrete or continuous predictors (here situation, pea-variety parameters, crop management techniques). The data set is recursively split into two subsets along a threshold value of the predictor in order to maximize the difference between subsets (see example in Figure 2). Branches are combinations of predictor values that lead to predictions contained in leaf nodes. The trees were computed with the R package mvpart (Glenn De’ath, 2014). The optimal tree size was internally identified using a 10-fold cross-validation procedure to avoid overfitting. In the following, R^2^ refers to the cross-validated R^2^ of the resulting tree.

**TABLE 3:** Variability in weed (dis)service indicators is explained by the different types of simulation factors.

Type of explanatory variables (CART predictors)	Yield		Weed services and disservices				Performance profiles	
	Potential (without weeds)	Actual (with weeds)	Yield loss	Field infestation	Bee food	Species richness	Integrated	Agroecology^$^
		
	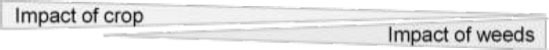							
**A. Spring pea – Years with pea**								
Pea parameters	0.72	0.36	0.11	0.03	0.01	0.01	0.19	0.11
Pea techniques	0.04	0.14	0.22	0.26	0.11	0.15	0.30	0.22
Other-crop techniques	0.01	0.12	0.14	0.26	0.19	0.12	0.28	0.25
Situation	0.00	0.02	0.07	0.07	0.08	0.16	0.03	0.06
TOTAL	0.77	0.64	0.54	0.61	0.40	0.44	0.80	0.65
**B. Spring pea – Average over rotation**								
Pea parameters	0.42	0.22	0.03	0.04	0.02	0.03	0.11	0.04
Pea techniques	0.04	0.17	0.17	0.13	0.15	0.06	0.25	0.13
Other-crop techniques	0.21	0.33	0.39	0.45	0.37	0.33	0.46	0.50
Situation	0.16	0.06	0.18	0.20	0.17	0.33	0.04	0.02
TOTAL	0.83	0.78	0.77	0.81	0.71	0.75	0.85	0.69
**C. Winter pea – Years with pea**								
Pea parameters	0.60	0.27	0.18	0.02	0.01	0.02	0.22	0.28
Pea techniques	0.07	0.14	0.13	0.11	0.11	0.11	0.34	0.17
Other-crop techniques	0.02	0.10	0.12	0.25	0.16	0.12	0.17	0.18
Situation	0.00	0.07	0.13	0.10	0.09	0.16	0.10	0.06
TOTAL	0.70	0.57	0.56	0.49	0.37	0.42	0.83	0.69
**D. Winter pea – Average over rotation**								
Pea parameters	0.30	0.14	0.06	0.04	0.04	0.04	0.16	0.06
Pea techniques	0.08	0.12	0.06	0.06	0.13	0.07	0.12	0.16
Other-crop techniques	0.30	0.29	0.37	0.45	0.34	0.29	0.49	0.37
Situation	0.10	0.18	0.26	0.20	0.15	0.32	0.05	0.04
TOTAL	0.78	0.73	0.75	0.75	0.66	0.71	0.81	0.63

*Partial R^2^ of descriptors from classification and regression trees (CART) were summed per type of simulation factor; CARTs were run separately on cropping systems including spring vs. winter pea and considering either only years with pea or averages over all simulated years (note that yield then includes yield from crops other than pea). Cells were colored from white (0, no impact) through yellow (0.5, intermediate impact) to green (1, highest impact). Gray triangles schematize the relative impact of crops and weeds on indicators. ^§^The “Integrated” profile aggregates weed-infested yield and herbicide use reduction, and the “Agroecology” profile moreover considers bee food offer.*

**TABLE 4:** The key pea parameters and techniques driving potential yield and yield loss due to weeds in Spring (S) and Winter (W) pea.

A. Pea parameters (Parameter names terminating in “early,” “mid” or “late” respectively concern plants at BBCH stages [0,4], [4,8] or [8,10])			Min-max in pea varieties (other crops)^$^	Prob of increase^§^		Reasons for effect (based on the analysis of simulated state variables)
Name	Meaning	Unit		Potential yield	Yield loss control	

Germination and pre-emergent growth						
darknessReduction	Reduction in germination if seeds are in darkness	seeds/seeds	0.90; 1.10* (0.40; 1.00)		S W 0.00	Fewer crop seeds germinate and emerge, reducing crop canopy density and leaving more space/light to weeds

g0	Time from germination triggering (sowing if moist soil, post-sowing rainfall otherwise) to first germinated seed	°C days	15.0; 24.9 (6.4; 33.3)		S W 0.00	Crop mergence is delayed, protecting spring-sown crops from frost. Crop germination and emergence are delayed, reducing crop growth duration

rb	Shape parameter for pre-emergent root growth. The higher this value, the later the growth onset but the higher the growth speed	none	1.24; 1.52* (1.24; 1.52)		S W 1.00	Pre-emergent crop root elongation starts earlier, thus reducing crop seedling loss due to early drought

gamma	Increase of seedling mortality with seed depth	seedlings⋅ seedlings^–^^1^*CPSTABLEENTER*⋅lnmm^–1^	0.30; 0.37* (0.41; 0.58)		S W 0.00	More crop seedling loss during pre-emergent growth

shootDiameter	Shoot diameter during pre-emergent shoot growth	mm	1.98; 2.42 (0.44; 2.20)		S W 1.00	Lower pre-emergent seedling mortality in buried crop seeds

**Potential morphology (in the absence of shading)**						

Emax	Potential maximal extension of the root system	mm	213; 301 (191; 363)		S W 1.00	A more voluminous crop root system leaves less water for weed seed germination and pre-emergent growth

max_height	Maximum plant height	cm	15.0; 24.9 (6.4; 33.3)		S W 0.00	Taller crop plants grow above weeds, increasing crop biomass production and shade cast on weeds, and this from emergence onward

LBRlate	Leaf biomass ratio (leaf biomass/total above-ground biomass) during reproduction if no shading	g/g	0.27; 0.59 (0.00; 0.98)	S 1.00 W		Crop plants have a larger light interception area, increasing crop biomass production and shade cast on weeds

WMearly	Plant width per above-ground plant biomass after emergence if no shading	cm/g	4.31; 66.18 (0.01; 100.00)	S 0.00 W		Unshaded crop plants are wider per unit biomass and more prone to be shaded by taller neighbor plants and more exposed to damage by mechanical weeding

b_WMearly	Sensitivity of plant width to above-ground plant biomass after emergence (b_WM = 0: plant width is constant; b_WM > 0: plant width increases with plant biomass)	none	0.17; 0.79 (0.05; 0.75)	S 1.00 W		Heavier crop plants are wider, increasing soil coverage and light interception by the crops, increasing crop biomass production and shade cast on weeds

b_RLHlate	Unevenness of leaf area distribution along plant height during reproduction. The lower this parameter, the more uniformly leaf area is distributed along plant height	none	1.94; 4.62 (1.28; 20.64)	S 0.00 W		Crop leaf area is distributed unevenly along plant height in crops resulting in a heterogeneous canopy

**Shading response**						

mu_HMmid	Increase in plant height per biomass HM if shaded during vegetative stage	none	−0.30; 0.61 (0.00; 1.77)	S 1.00 W		Shaded crop plants increase their height per unit per unit biomass to grow above weeds, increasing crop biomass production and shade cast on weeds

mu_LBRlate	Increase in leaf biomass ratio LBR if shaded during reproduction	none	−0.33; 0.12 (−0.62; 1.39)	S 1.00 W		Shaded crop plants increase leaf area. Crop plants intercept more light, produce more biomass and cast more shade onto weeds

**Plant phenology**						

PeaFloweringTT	Time from plant emergence to flowering onset	°C days	82; 2696 (434; 4701)	S 1.00 W 0.91		More crop biomass is produced before reproduction sets inA longer crop growth duration delays the harvest and leaves more time for the weeds to produce biomass inside the crop

**Temperature requirements**						

tPhoto2	Temperature above which net photosynthesis is maximum	°C	15; 27 (10; 30)	S 0.00 W		Maximum crop photosynthesis rate is reached only on hot days, reducing crop biomass production

**B. Pea techniques**		**Min-max in data set^$^**	**Probability of increase^§^**			**Reasons for effect (based on the analysis of simulated state variables)**
**Meaning**	**Unit**		**Potential yield**	**Yield loss control**	**Field infestation control**	

**Tillage**						

Superficial tillage (disregarding rolling)	Number per year	0; 10			S W 0.60	Empties weed seed bank by stimulating germination during summer fallow (“false seed bed technique”), destroys weed plants prior to crop sowing. But stimulates weed seed germination and emergence of excavated weed seeds which can emergence after crop sowing

Maximum tillage depth	cm	0; 29		S 0.17	S 0.06 W	Buries more weed seeds, reducing weed seed germination and emergence. But stimulates weed seed germination and emergence of excavated weed seeds

				W 1.00		

Tillage depth averaged over all tillage operations (excluding rolling and shredding)	cm	0; 20		S 0.22 W	S 0.24 W	Buries more weed seeds, reducing weed seed germination and emergence. But stimulates weed seed germination and emergence of excavated weed seeds

Residue shredding during summer fallow	Number per year	0; 2		S 1.00 W 1.00		Destroys weeds during summer fallow when they are most important for feeding bees

Time from previous harvest to first tillage operation	days	1; 285			S 0.09 W 0.02	More time for weed seeds to be imbibed by rain on soil surface and getting more sensitive to false seed bed operations. But leaves more time for summer annuals to grow before destroying them. Less time to till frequently, larger risk of triggered weed seed germinations resulting in weeds emerging after crop sowing

Time from last tillage to crop sowing	days	0; 127		S 0.00 W 0.00	S 0.00 W 0.00	Leaves more time for weeds to reinfest the field after the last tillage operation cleaned out all weeds

**Pea sowing**						

Sowing date	Julian days	1 Oct; 15 Apr	S W 0.85	S 0.02	S 0.01 W 0.00	Leaves more time for false seed bed techniques; shortens the time during which weeds can grow inside the crop. But shortens the crop growth duration

				W 0.67		

Sowing density	seeds/m^2^	25; 115	S 1.00 W 0.89			More crop plants and leaf area to produce crop biomass and shade weeds. But crop-crop competition can hinder crop biomass production in very high crop densities

Interrow width if row sowing	cm	4; 52	S W 0.00			More competition between plants inside a given row, more light unintercepted by crops inside interrow

**Harvest**						

Pea leaves and stems were left in the field after harvest	1 (yes) or 0 (no)	0; 0.8		S 1.00 W 1.00	S 0.41 W	Hinders weed emergence after harvest. But leaves harvested weed seeds in filed

**Herbicides and Mechanical weeding**						

Time from sowing to 1st herbicide spraying	days	1; 294			S W 0.71	Earlier treatments miss later emerging weeds

Total mortality of monocot weeds due to herbicides	[0,1]				S W 0.99	Fewer weeds survive

Mechanical weeding operations	Number per year	0; 5		S W 1.00		Destroys weed plants inside the crop

The most influential parameters and techniques per indicator were selected based on relative variable importance predictor (VIP > 0.1) in classification and regression trees analyzing weed (dis)service indicators of years with pea as a function of pea parameters, pea management techniques, and other-crop management techniques. ^§^Probability cells were colored from green (1, 100% probability that an increase in parameter/technique values results in an increased indicator value) to red (0, 100% probability that an increase in parameter/technique values results in a decreased indicator value) through white (0.5, 50% for both increased and decreased indicator values depending on interactions with other parameters or techniques). Empty cells indicate that the parameters/techniques had no significant effect on the indicator. For complete results, see sections E.2–E.7 of the Supplementary Material. *This parameter does not vary for actual varieties as variety-specific information was available when parameterizing pea in FLORSYS. ^§^ if b_WM is zero, plant width does not depend on plant biomass; the larger b_WM is, the more width increases with biomass. ^$^5 and 95% percentiles of values.

**TABLE 5:** Optimal combinations of pea parameters, pea management, and other-crop management, depending on the goal, situation, analysis scale, and pea variety type (complete results in section E.8 of the Supplementary Material). The three best scenarios (branches B1, B2, and B3) identified with classification and regression trees applied to 400 cropping systems × 12 years × 10 weather repetitions per situation. Indicator values were normed to [0, 1] with 0 the worst performance (i.e., lowest yield or highest yield loss) over all situations and 1 the best one (i.e., highest yield or lowest yield loss). In the case of multivariate response variables, branches were ordered by increasing values of the minimum of the response variables (see section “Statistics”). A and C: rules were colored from green (100% of the parameter range of variation of actual varieties consistent with the rule) to red (0% of the range covered) through white (50%).

A. Potential yield (without weeds) - Herbicides in pea - Years with spring pea (tree of 35 branches, leaf averages = [0.001, 0.58]).								
Name	Meaning	Stage/Condition	Unit	Min, max in actual pea varieties	B 1	B 2	B 3	Rank in tree
						
					*N* = 6%	*N* = 5%	*N* = 5%	
					*M* = 0.58	*M* = 0.53	*M* = 0.52	

Pea parameters								
**tPhoto2**	**Temperature above which net photosynthesis is maximum**		**°C**	**15, 27**	**<19.6**			**1**

**WMearly**	**Plant width per above-ground plant biomass if no shading**	**Post-emergence**	**cm/g**	**4.3, 66.2**	**<32.1**			**1**

**b_WMearly**	**Sensitivity of plant width to above-ground plant**	**Post-emergence**	**none**	**0.17, 0.79**	**≥0.427**			**1**
b_WMmid	**biomass** (the larger b_WM, the more width with	Vegetative	none	0.21, 0.60	<0.434			4
b_WMlate	biomass)	Reproduction	none	0.23, 0.43	≥0.323			4

LBRearly		Post-emergence	g/g	0.72, 0.83	<0.821			4
LBRmid	**Leaf biomass ratio if no shading**	Vegetative	g/g	0.75, 0.82	<0.805			4
**LBRlate**		**Reproduction**	**g/g**	**0.27, 0.59**	**≥0.45**			1

SLAlate	Specific Leaf Area SLA if no shading	Reproduction	cm^2^/g	58.6, 139	<108			4

RLHearly	Relative leaf area height (relative plant height below which	Post-emergence	cm/cm	0.48, 0.62	<0.573			4
RLHmid	50% of leaf area are located) if no shading	Vegetative	cm/cm	0.44, 0.58	<0.536			4

b_RLHearly	**Unevenness of leaf area distribution along plant**	Post-emergence	none	2.02,3.01	<2.97			4
**b_RLHlate**	**height** (low values indicate a uniformly distributed leaf area)	**Reproduction**	**none**	**1.94, 4.62**	**<3.15**			**1**

HMearly		Post-emergence	cm/g	6.5, 25.5	≥12.5			4
HMmid	Plant height per above-ground plant biomass if no shading	Vegetative	cm/g	11.7, 22.1	≥14.1			4
HMlate		Reproduction	cm/g	15.6, 50.4	≥22.0			4

b_HMlate	Sensitivity of plant height to above-ground plant biomass	Reproduction	none	0.12, 0.37	<0.321			4

**mu_LBRlate**	**Increase in leaf biomass ratio LBR if shaded**	**Reproduction**	**none**	**−0.33, 0.12**	**≥-0.09**			**1**

**mu_HMmid**	**Increase in plant height per biomass HM if shaded**	**Vegetative**	**none**	**−0.30, 0.61**	**≥0.220**			**1**

mu_SLAearly	Increase in specific leaf area SLA if shaded	Post-emergence	none	0.04, 0.13	<0.112			4

PeaFloweringTT	Time from plant emergence to flowering onset		°C days	846, 4824	≥295			2

g0	Time from germination triggering (sowing in moist soil, rain) to first germinated seed		°C days	15, 24.9	<23.4			4

g50	Time from germination triggering to 50% germinated non-dormant seeds		°C days	29, 32	<31.6			4

gb	Shape parameter for germination progress (large gb = later and faster germination)		none	2.18, 2.50	≥2.29			4

LA0	Initial leaf area at emergence	Emergence	cm^2^	0.47, 3.98	≥0.980			4

se_LA	Standard-deviation of initial post-emergence leaf area	Emergence	cm^2^	0.39, 1.96	≥0.554			4

rateCyl	Depth at which the root system extension is maximal/root system depth		cm/cm	0.07, 0.26	<0.196			4

soilPen	Resistance to soil compaction (0 = none, 1 = total)		cm/cm	0.82, 1.00	<0.969			4

max_width	Maximum plant diameter		cm	69, 132	≥88.5			4

tPhoto1	Temperature at which photosynthesis starts		°C	0.00, 1.10	≥0.200	<0.200		7

baseTempDev	Base temperature for development		°C	0	≥0.057	<0.0577		7

TTflo	Duration of flowering stage		°C days	400, 950	≥570	<570		7

TTmat	Duration of maturation		°C days	490, 670	<621	≥621		7

baseWP	Base water potential for germination		MPa	−2.30	<−2.31	≥−2.31		7

nonDormantMin	Minimum proportion of non-dormant seeds		seed/seed	1.00	<0.974	≥0.974		7

g0	Time from germination triggering to first germinated seed		°C days	15.0, 24.8	≥16.3	<16.3		7

darknessReduction		If seeds are in darkness	seed/seed	1.00	**≥**1.00	<1.00		7

reductionSurface	Reduction in germination	If seed is on soil surface	seed/seed	0.54	**≥**0.540	<0.540		7

reductionDepth		With seed depth	se⋅se^–1^⋅cm^–1^	0.00059, 0.00091	**≥**0.0006	<0.0006		7

shootDiameter	Shoot diameter	Pre-emergence	mm	2.20	<2.18	**≥**2.18		7

shootLength	Maximum shoot length (heterotrophic growth)	Pre-emergence	mm	363, 437	<425	**≥**425		7

rootLength	Maximum root length (heterotrophic growth)	Pre-emergence	mm	54.8, 65.1	<61.5	**≥**61.5		7

r50	Time until the seedling reaches 50% of rootLength	Pre-emergence	°C days	100, 122	<113	**≥**113		7

rb	Shape parameter for root growth (large rb = later and faster growth)	Pre-emergence	none	1.38	**≥**1.42	<1.42		7

gamma	Increase of seedling mortality with seed depth	Pre-emergence	sl⋅sl^–1^⋅mm^–1^	0.34	**≥**0.336	<0.336		7

C0surface	Smallest surface clod causing seedling death	Pre-emergence	mm	36.3	**≥**36.4	<36.4		7

LA0	Initial leaf area	Emergence	cm^2^	0.47, 3.98	**≥**1.40	<1.40		7

se_LA	Standard-deviation of leaf area	Emergence	cm^2^	0.39, 1.96	**≥**0.665	<0.665		7

RGR	Relative growth rate after emergence	Emergence	cm^2^/°C day	0.009, 0.027	<0.0197	**≥**0.0197		7

b_RLHmid	Unevenness of leaf area distribution along plant height	Vegetative	none	1.99, 2.67	<2.47	**≥**2.47		7

	**Varieties corresponding to these rules**				Virtual2 Virtual7	Kayanne	Kayanne Virtual2 Virtual7	

**Pea techniques**								

	Sowing density		seeds/m^2^	25, 115	**≥**56.5			3

	Sowing date			10 Jan, 15 Apr	<13 Apr			5

	Interrow width if row sowing		cm	4, 52	<34.5		**≥**34.5	6

**B. Weed-infested yield - Herbicides in pea - Years with spring pea (tree of 71 branches, leaf averages = [0.01, 0.55]).**								

**Branches**	**B 1**			**B 2**		**B 3**		

**N (prop of total)**	**0.1%**			**1.1%**		**0.9%**		

**Mean [0,1]**	**0.55**			**0.53**		**0.53**		

**Pea parameters**								

Main rules	Same 7 first rules as in Table 5A (old plants keep their leaves, shading → taller & leafier plants, max photosynthesis at lower temperatures, uniform leaf distribution along plant height etc.)							

Main difference with Table 5A	Shorter growth period: PeaFloweringTT in [190.1, 670.3]							

Root parameters	6 (vs. 2 in Table 5A) slowing down root-system extension and limiting it to superficial layers					

Potential plant morphology	High leaf area and leaf biomass (vs. tall plants irrespective of biomass in Table 5A)					

Germination parameters & pre- and post-emergent growth	3 (vs. 5 in Tables 5A–B1) slowing down emergence and early growth					

Germination parameters & pre-emergent growth	Similar to Tables 5A–B1 (susceptible to depth and compaction)				

Temperature, phenology	No rules (other than PeaFloweringTT) in contrast to Table 5A							

Varieties corresponding to these rules (in **bold**, same as for Table 5A)	**Virtual7**			Cameor **Virtual2 Virtual7**		Cameor **Kayanne Virtual2 Virtual7**		

**Pea techniques**								

Time from last tillage to sowing	≥81 day			<21 days		21-46 day		

Sowing date	<10 March					<10 March		

Herbicides with >50% efficiency on dicots				<2.5 ops/year				

Herbicide efficiency on dicots	≥93%							

Herbicide spectrum (% species killed at >90%)	≥50%							

**Rotation**								

	Rotation≥3.5 years							

	Pea frequency <0.29					No Wheat before Pea		

	Wheat frequency<0.29							

**Other-crop techniques**								

Average over rotation	Max tillage depth ≥12 cm							

	<0.29 rolling ops/year							

Wheat				Systemic herbicides ≥ 0.5/year		Variety in {Caphorn, Orvantis, Virtual1, V2, V4, V5, V6, V8, V9}		

				Sowing < 12 Oct		1*^st^* herbicide < 64 days after sowing		

Barley	Sowing < 20 Jun							

**C. Weed-infested yield - Herbicides in pea - Years with winter pea [tree of 94 branches, leaf averages = (0.03,0.67)].**								

**Name**	**Meaning**		**Unit**	**Min, max in actual pea varieties**	**B 1**	**B 2**	**B 3**	**Rank in tree**

					**0.1%**	**0.1%**	**0.9%**	

					***M* = 0.67**	***M* = 0.56**	***M* = 0.50**	

**Pea parameters**								

rateWidth	Speed of root system width extension		mm/day under optimal temperature	4.04, 6.00	≥4.12		<4.12	1

rateDepth	Speed of root system depth extension			10.5, 15.3	≥11.2		<11.2	1

mu_LBRmid	Increase in leaf biomass ratio LBR if shaded (vegetative stage)		none	–0.30, 0.01	≥−0.13			3

	Varieties corresponding to these rules				886-1 DCG0449 Virtual6		Isard	

**Pea techniques**								

	Time from last tillage to crop sowing		days	0, 127	<24.2	≥24.2		4

**Other-crop techniques**								

	Mean depth of mechanical weeding in rotation		cm	0, 5	≥3.4			5

	Mean tractor speed during mechanical weeding in Wheat		km/h			≥13.75		5

	Mouldboard ploughing April–October in Wheat		yes or no	0, 1			yes	3

	Time from last mechanical weeding to Oilseed rape harvest		days		<19			6

**Situation**								

	Initial weed seed bank is 6 harmful dicots promoting bee food				No	No	Yes	2

**D. Weed-infested yield - No herbicides in pea - Years with spring pea [tree of 39 branches, leaf averages = (0.01, 0.63)].**								

**Branches**	**B 1**			**B 2**		**B 3**		

N (prop of total)	0.7%			2.1%		4.0%		

Mean [0,1]	0.63			0.56		0.51		

**Pea parameters**			

Main rules	Same 7 first rules as in Table 5B (old plants keep their leaves, shading taller & leafier plants, max photosynthesis at lower temperatures, uniform leaf distribution along plant height etc.							

Main difference with Table 5B	Minimum growth period: PeaFloweringTT **≥**198°C days (which is much lower than all actual varieties)							

							Early & fast germination, tall & top-heavy plants, shading taller, narrower & top-heavier plants		

Varieties corresponding to these rules (in **bold**, same as in Table 5B)	**Cameor Kayanne Virtual2 Virtual7**					**Kayanne Virtual2**		

**Pea techniques**(in **bold**, same techniques as in Table 5B)			

Time from **last tillage** to crop sowing	<82 days							

**Sowing date**	<6 April							

Mechanically weeded field area	<52%					**≥**52%		

**Other-crop techniques:** Type (number of rules) (in **bold**, same techniques as in Table 5B)								

Wheat	Manure (2), **Sowing** (2), Rolling (1), Tillage (5), Mechanical weeding (3)			Manure (2), **Sowing** (2), Rolling (1), Tillage (5), Mechanical weeding (4), **Variety** (1)		Manure (1), **Variety** (2)		

Oilseed rape	Ploughing (1), Sowing (2)			Ploughing (3), Sowing (3), Tillage (3), Rolling (1), Mechanical weeding (1), Harvest (1)		Mechanical weeding (1)		

Average over rotation	**Tillage** (2), Mechanical weeding (2), Manure (1), Crop-residue shredding (1)			**Tillage** (2), Mechanical weeding (4), Manure (1), Crop-residue shredding (2)		Mechanical weeding (1)		

**E. Weed-infested yield - No tillage in pea - Years with spring pea [tree of 23 branches, leaf averages = (0.01,0.54)].**								

**Branches**	**B 1**			**B 2**		**B 3**		

N (prop of total)	1.6%			1.2%		0.6%		

Mean [0,1]	0.54			0.49		0.46		

**Pea parameters**								

Main rules	Same 7 first rules as in Table 5B (old plants keep their leaves, shading taller & leafier plants, max photosynthesis at lower temperatures, uniform leaf distribution along plant height etc.)							

Main difference with Table 5B	Higher minimum growth period: PeaFlowering TT **≥**329°C days (which is still much lower than all actual varieties)							

Other	No other rules (vs. 25 other rules for Tables 5B–B2, 9 further rules for Tables 5B–B1)							

Varieties corresponding to these rules (In **bold**, same as in Table 5B)	**Cameor Kayanne Virtual2 Virtual7**							

**Pea techniques** (in **bold**, same techniques as in Table 5B)								

Sowing density	**≥**107 seeds/m^2^			<107 seeds/m^2^				

**Other-crop techniques:** Type (number of rules) (in **bold**, same techniques as in Table 5B)								

Wheat	**Variety** (1)			**Variety** (1), Ploughing (2), Tillage (3), Mechanical weeding (3), Irrigation (2)		**Variety** (1)		

Oilseed rape				Sowing (2), Tillage (1), Rolling (1)		Tillage (1)		

Average over rotation	Herbicides (1)			Herbicides (2)		Herbicides (2), **Tillage** (3), Ploughing (1), Mechanical weeding (2), Irrigation (1), Crop-residue shredding (1)		

**F. Weed-infested yield - No tillage - Average over rotation including spring pea [tree of 31 branches, leaf averages = (0.09,0.53)].**								

**Branches**	**B 1**			**B 2**		**B 3**		

N (prop of total)	0.3%			0.3%		2.8%		

Mean [0,1]	0.53			0.51		0.43		

**Pea parameters**								

Main rules	Same 8 first rules as in Table 5E (long growth period, old plants keep their leaves, shading taller & leafier plants, max photosynthesis at lower temperatures, uniform leaf distribution along plant height etc.)							

Main difference with Table 5E	None							

Varieties corresponding to these rules (in **bold**, same as in Table 5E)	**Cameor Kayanne Virtual2 Virtual7**							

**Pea techniques** (in **bold**, same techniques as in Table 5E)								

	Sown < 20 March			Sowing depth **≥**4.5 cm		Sowing density **≥**88 seeds/m^2^		

**Other-crop techniques:** Type (number of rules) (in **bold**, same techniques as in Table 5E)								

Wheat	Sowing (1), Harvest (1)			Sowing (4), Herbicides (3)		Sowing (2), Herbicides (2)		

Oilseed rape	Herbicides (2)			**Sowing** (1), Herbicides (2)		Herbicides (4)		

Average over rotation				**Herbicides** (2)		**Herbicides** (5)		

Predictors (including surrogate variables that would split the data set exactly as the primary splitting variables) were ranked according to their relative Variable Importance Predictor (VIP, rescaled here by dividing by the total amount of variability explained by the tree, see section D.4 of the Supplementary Material). The CARTs were built, allowing up to “perfect” 20 surrogates (i.e., with raw agreement = 1) per split. In addition, the probability that the analyzed response variable increases when the predictor value increases were estimated by summing the relative number of individuals that split the branch toward a higher predictor value, over all tree nodes including the predictor as a primary or surrogate splitting variable. We defined the partial R^2^ of a predictor as its relative VIP multiplied by the total R^2^ of the tree. To assess the contribution of predictor classes (situation, pea parameters, pea techniques, other-crop techniques), partial R^2^ was calculated for each class, summing partial R^2^ over all predictors belonging to the class.

Finally, optimal combinations of pea parameter values and crop management techniques were identified for different performance goals, per variety type (spring or winter), analysis scale (years with pea or average over-rotation including all crops), and situation. Several individual performance goals were considered, maximizing either potential pea yield (i.e., yield in weed-free simulations), weed-infested pea yield (from weed-inclusive simulations), weed-based trophic resources for domestic bees, or weed species richness, or minimizing either crop yield loss due to weeds or field infestation. In addition, composite goals were included, called “Integrated” (maximizing weed-infested yield and minimizing herbicide use intensity), and “Agroecology” (the former plus maximizing bee-food offer). To identify these optimal combinations, CARTs were run per goal, variety type, and analysis scale (years with pea vs. average over-rotation). The three best branches were selected, i.e., those leading to the leaves with the highest indicator value (remember that, e.g., the lowest yield loss gives the highest rescaled indicator value). For multi-variate goals, the best branches were those with the highest minimum value of the constituting indicators. The best branch b of a given tree answers to the following condition:


$$\min_{\mathrm{over}i}\left({\text{T}}_{\text{bi}}\right)>\max_{\mathrm{over}b^{\prime }}\left(\min_{\mathrm{over}i}\left({\text{T}}_{b^{\prime }i}\right)\right)$$

where T_*bi*_ is the value of the indicator i for branch b.

Here, only a few examples were presented, the complete results are in sections E and F of the Supplementary Material.

## Results

### Parameterizing Contrasting Pea Varieties

The garden-plot experiments allowed estimating the parameters related to potential plant morphology and shading response. These parameters were among those that the most discriminated the seven pea varieties (see section C.3 of the Supplementary Material). Differences between varieties depended on the analyzed parameter (Figure 1). There was, for instance, little difference among varieties for the leaf biomass ratio, i.e., the ratio of leaf biomass divided by aboveground plant biomass (Figure 1E). This ratio was around 0.8 until nearly flowering onset and then continuously decreased until full maturity, regardless of the variety. Other parameters differed according to the seasonal type, e.g., at early stages, specific leaf area (SLA, ratio of total leaf area divided by total leaf biomass) was lower for the two spring varieties (Cameor and Kayanne) tested here than for the five winter varieties (except Isard, Figure 1A). But, most parameters greatly varied among varieties, regardless of seasonal or morphological type (i.e., afila vs. leafy).

### Trade-Offs Among Indicators of Pea Yield and Weed (Dis)services

**FIGURE 3:**
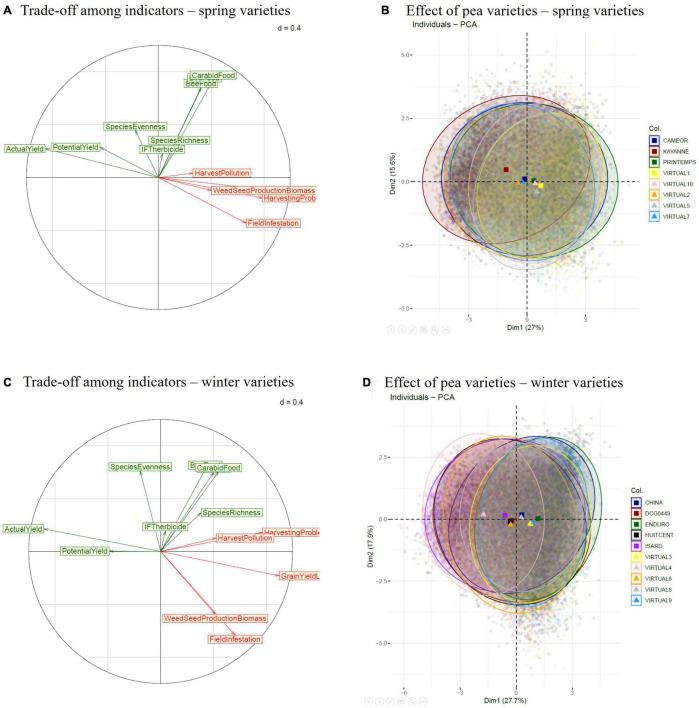
Trade-offs among indicators of pea yield and weed (dis)service identified with Principal Component Analyses (PCA) for spring **(A,B)** and winter pea varieties **(C,D)** on the nine simulated situations, using annual simulated data. Pea-yield and biodiversity indicators are shown in green, weed harmfulness for pea production is in red (Colbach, 2020 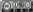).

Across the nine simulated situations, weed-infested pea yield (in the presence of weeds) was opposed to direct weed harmfulness for pea production (yield loss, harvest pollution by weed seeds and debris, harvesting problems) in the Principal Component Analysis (PCA), regardless of the pea variety type (Figures 3A,C). The potential yield (yield from weed-free simulations) was badly represented in the first two PCA axes (the corresponding arrow was short) and was little correlated to yield loss due to weeds (Pearson correlation coefficients = −0.23 and −0.30 for spring and winter varieties, respectively). This means that the situations maximizing potential pea yield were not necessarily good at controlling weeds, and vice-versa. Correlations among weed-harmfulness indicators were higher in spring pea (Figure 3A) than winter pea (Figure 3C). Irrespective of the variety type, weed harmfulness was not or little correlated to either herbicide use intensity or biodiversity (weed species richness and evenness, trophic resources for birds, carabids, and pollinators).

### Pea Varieties Differ in Terms of Yield and Weed (Dis)services

The ellipses corresponding to the different pea varieties were very large, indicating that their performance in terms of pea yield and weed (dis)services varied greatly with the situation, cropping system, year, weather repetition (Figures 3B,D). These ellipses mostly overlapped, indicating that simulation factors other than pea variety explained most of the variability. Despite this, a few tendencies could be observed. In spring pea, varieties were roughly ranked along a line ranging from yield to field infestation (Figure 3B). The best-performing variety was an actual one, i.e., Kayanne, the worst-performing varieties were all virtual ones. Winter pea varieties were ranked roughly along a line from weed-infested yield to yield loss due to weeds, with larger differences among varieties than for spring pea (Figures 3D vs. B). Actual varieties were all outperformed by a virtual one (Virtual 4), and the worst variety was an actual one (Enduro).

The trade-off between pea yield in the absence of weeds (i.e., potential yield) and pea resilience toward weeds (i.e., yield loss due to weeds) as well as crop impact on weeds (i.e., field infestation) was obvious on actual varieties (Table 1B). The variety with the highest potential yield (Isard) was intermediate in terms of yield loss and field infestation. Conversely, the variety with the highest field infestation (Cameor) was intermediate in terms of potential yield and yield loss. The two spring varieties (Cameor and Kayanne) presented the lowest weed-related yield loss and almost the best yield potential. Otherwise, there was no obvious link between average variety-performance indicators and major variety characteristics, i.e., leaf morphology (Cameor, China and DCG0449 did not differ from the rest), seed mass or maximum plant height (no correlation between these two traits and any of the three indicators of Table 1B).

There was also a trade-off between the average performance of actual varieties and their stability across situations, years, and weather repetitions (sd in Table 1B). For instance, the variety with the lowest average potential yield (China) had the second-highest yield stability; the variety with the most stable yield loss (Enduro) was also the one with the highest yield loss. Conversely, the variety with the highest field infestation (Cameor) also presented the most variable one. Generally, spring varieties (Cameor and Kayanne) presented the highest variability, regardless of the analyzed indicator.

### Which Simulation Factors Drive Potential Yield and Weed (Dis)services?

The more detailed analysis of the pea-variety impact with the help of the classification and regression trees (CART) showed that this impact greatly varied with the analyzed indicator and scale (Table 3). The more a weed (dis)service indicator was related to crop growth and production, the larger the effect of pea parameters was (larger partial R^2^ in Table 3) and the lower the effects of management techniques and situation were (lower partial R^2^ in Table 3). Potential yield (i.e., from the weed-free simulations) mostly depended on pea parameters (higher partial R^2^ than for other simulation factors in Tables 3A,C). The response of pea to weeds also depended on pea parameters as shown by weed-infested yield and weed-caused yield loss but the latter depended much more on crop management. The effect of pea parameters was even smaller when looking at indicators translating weed response to pea and management, whether quantitative (i.e., field infestation which is based on weed biomass) or qualitative (bee food offer and species richness which are based on weed traits and/or species).

The CART analyses of Table 3 confirmed some tendencies already identified with the Principal Component Analysis of Figure 3. For potential pea yield, pea parameters were more important in spring pea (Table 3A) than in winter pea (Table 3C) and vice-versa for pea techniques; for yield loss, the opposite was true, i.e., pea parameters were most important in winter pea and pea techniques most important in spring pea. Explained variability was smaller for weed-impact indicators than for potential yield, pointing to stronger interactions among simulation factors and to a larger impact of effects that were not included in the analysis, i.e., weather and stochastic effects (e.g., weed plant location). Generally, the effect of both pea parameters and pea management techniques was more visible during the years with pea (Tables 3A,C) than at the rotation scale (Tables 3B,D) where these effects were overshadowed by other-crop techniques. This was particularly true for rotation-scale yields (potential and weed-infested) as these indicators included yield data from crops other than pea. Though the management of crops other than pea was the most influential when analyzing weed (dis)services at the rotation scale, other-crop techniques also influenced both pea yield and, particularly, weed impacts in pea crops at the annual scale (Tables 3A,C).

The effect of simulation factors on the indicators of the composite performance profiles (“Integrated” and “Agroecology”) varied with varieties and scales. But generally, pea parameters were the most important for winter varieties, and pea techniques for spring varieties; the opposite was true for other-crop management. The situation had no effect at the rotation scale, and variability was least well explained for the “Agroecology” profile.

### Which Pea Parameters and Techniques Drive Potential Yield and Yield Loss?

Table 4 lists the main pea parameters (Table 4A) and techniques (Table 4B) driving potential yield and weed-caused yield loss in pea as well as the explanations for these effects, based on the functioning of the FLORSYS model. The results on the other-crop techniques, the other weed-impact indicators, and from the rotation scale are in section E of the Supplementary Material. In short, any parameter value which delays and/or reduces crop emergence (e.g., large *darknessReduction* or *gamma* values) decreased potential yield and, particularly, yield-loss control. Conversely, any parameter value which increases crop canopy volume (e.g., large *LBRlate* or *mu_HMmid* values) and crop growth duration (e.g., large *PeaFloweringTT* values) had the opposite effect (Table 4A). Shading response (*mu* parameters) was crucial: the more pea plants increase plant height (large *mu_HMmid* values) and leaf biomass (large *mu_LBRlate*) when shaded, the better. However, key parameters completely differed between pea-variety types and indicators: for instance, parameters driving germination and pre-emergent growth were crucial for yield-loss control in winter pea but not in spring pea or for potential yield. Field infestation was little affected (details in section E).

Interestingly, almost all parameters either always increased (probability values in Table 4A = 1) or always decreased indicator values (probability = 0), indicating a stable effect regardless of other parameters and management techniques. Three of the key parameters (*darknessReduction, gamma, rb*) did not vary among actual varieties as variety-specific information was unavailable when parameterizing pea in FLORSYS. All three concern pre-emergent processes. All other parameters vary as much for virtual as for actual varieties. Generally, the range of variation of the key pea parameters was smaller than the range for other crops and included in this latter range. The only exceptions concern plant morphology: when shaded, pea plants mostly reduced their leaf biomass ratio (*mu_LBR* < 0) whereas most other crops increase theirs (*mu_LBR* > *0*).

In terms of pea management, the technique driving crop growth duration, i.e., *sowing date*, was the most influential factor, but its effect depended on the pea variety type and other interactions (Table 4B). Delayed spring-pea sowing deteriorated the control of yield loss and field infestation whereas the effect varied for delayed winter-pea sowing. Field-infestation control systematically decreased but yield potential and yield-loss control usually improved (because of, e.g., less frost damage). All the other key techniques presented a stable effect, irrespective of pea varieties, though the effect could vary (i.e., the probability was not 0 or 1) depending on other conditions (e.g., other management techniques). Potential pea yield mostly depended on a dense (high *sowing density*) and uniform crop canopy (*row sowing*, small *interrow width*) whereas the control of yield loss and field infestation mostly depended on tillage aiming to reduce the weed seed bank, with depth and timing being crucial for success. Herbicides were the most important for field infestation, mechanical weeding for yield loss.

### Optimize Pea Varieties and Management

With the help of the classification and regression trees, optimal combinations of pea parameters and crop management techniques were identified, depending on the performance goal, the situation, the pea variety type, and the decision scale (see examples in Figure 2 and Table 5, with the complete set of branches in section F of the Supplementary Material). The examples presented here focused on pea yield, and were chosen to illustrate the impact of weeds, pea variety type, weed-control strategy, and analysis scale.

#### Maximize Potential Yield in Spring Pea in Herbicide-Based Systems

To optimize potential yield in spring pea in herbicide-based cropping systems, the choice of pea parameters was crucial (Table 5A). A set of common 29 rules described the pea varieties of the three best tree branches, corresponding to respectively the 6, 5, and 5% situations with the highest potential yield among the 35 branches of the tree. For these best configurations, plants photosynthesized well at low temperatures (low tPhoto2). When unshaded, their width increased with increasing biomass after emergence and during reproduction (high b_WMearly and b_WMlate, low WMearly) but it was homogeneous during the vegetative stage (low b_WMmid). Plants were stemmy before reproduction (low LBRearly and LBRmid) but kept investing a large proportion of biomass in leaves during reproduction (high LBRlate) though the leaves were small and thick (low SLAlate). Leaf distribution along plant height was uniform (RLHearly and RLHmid close to 0.5, low b_RLHearly and b_RLHlate), and plants were tall per unit biomass at all stages (large HMearly, HMmid, and HMlate). Shaded plants compensated by investing more biomass into leaves (large mu_LBRlate), with larger thinner leaves (large mu_SLAearly) and became even taller per unit biomass (large mu_HMmid). Growth duration needed to exceed a minimum threshold (PeaFloweringTT), germination was early (low g0, g50) and fast (large gb), leading to well-established seedling (large LA0) (Table 5A). These advantages came at a cost, being linked to less favorable features, such as a reduced root system (low rateCyl), which was moreover sensitive to soil compaction (low soilPen).

Many of these rules were respected by all the actual pea varieties tested here, e.g., a low leaf biomass ratio after emergence (LBRearly < 0.821 g/g) or a minimum time from emergence to flowering (PeaFlwoeringTT ≥ 295°Cdays). The most discriminating rules were the seven that drove the first split in the tree (i.e., order = 1 in the tree). They concern diverse traits such as the ability to photosynthesize at low temperatures (low tPhoto2), to keep a high biomass ratio during reproduction (large LBRlate), or to compensate shading by increasing plant height (large mu_HMmid). In the end, only three varieties verified all 29 rules, i.e., Kayanne, Virtual2, and Virtual 7.

The best two branches consisted of further, opposing 21 rules. Two virtual varieties corresponded to the best branch B1 (Virtual2 and Virtual7). The three branches moreover differed in terms of pea management, with B1 and B2 requiring a narrower interrow than B3. All three branches needed a high sowing density and an early sowing date. Rotation and the management of crops other than pea presented negligible effects.

#### What Changes in the Presence of Weeds?

When looking at weed-infested yield (Table 5B), the main rules for spring-pea varieties in herbicide-based systems remained the same for the three best branches corresponding to the 2.1% best performances, except that the pea growth period needed to be limited (low PeaFloweringTT). The latter rule cuts off part of weed-seed reproduction and infestation of future crops. In the best two branches, the emphasis was more on high leaf area than for potential yield, to the detriment of root-system expansion. The latter was closer to the soil surface, reducing available water for weed seed germination and emergence. Another difference was a slower crop emergence, which increased the efficiency of pre- and post-sowing herbicides as early emerging weeds would be more exposed to herbicide droplets. The varieties corresponding to the three branches comprised the same three varieties as for potential pea yield, with the additional Cameor variety.

Compared to the weed-free branches, the optimal weed-infested branches considerably differed in terms of rotation and crop management. In pea, tillage and herbicides became more important than sowing patterns. The best branch was characterized by a high crop diversity. Herbicide options were important but not in all crops: if stringent rules were applied in pea (branch B1), none were needed in other crops, and vice versa (B2 and B3).

#### Are Optimal Parameter Values the Same for Spring vs. Winter Varieties?

The rules for winter pea in herbicide-based systems were completely different (Table 5C) from the spring-pea rules. Winter-pea rules concerned either a fast root-system extension (large rateWidth and rateDepth) and a strong shading response in terms of leaf-biomass increase (large mu_LBRmid) (branches B1 and B2) or a slow root-system extension (low rateWidth and rateDepth), leaving more above-ground biomass for light interception (B3). The latter variety-type though only worked if the weed flora consisted of the six harmful dicots that promote the bee-food offer. The first branches corresponded to two actual varieties (886-1 and DCG0449) and the last one to one actual variety (Isard). Crop-management rules focused on tillage and mechanical weeding, and the discrimination between branches B1 and B2 was based on the delay between the last tillage operation and pea sowing.

#### What Changes in Herbicide-Free Pea Crops?

The weed-infested yield was higher for unsprayed than for sprayed spring pea, ranging from 0.51 to 0.63 (with 1 = the best weed-infested yield observed for pea in all simulations) for the 6.8% best-unsprayed spring-pea crops (Table 5D) compared to 0.53–0.55 for the 2.1% best-sprayed ones (Table 5B). Two main strategies were identified for unsprayed spring pea (Table 5D). Branch B1 and B2 had few pea-variety rules which were the same as the main rules in sprayed pea. To be successful, these few rules had to be combined with a great many constraints for crop management, mainly in the three most frequent crops (pea, wheat, oilseed rape). Conversely, branch B3 mainly focused on mechanical weeding but compensated with additional pea-variety rules, ensuring early crop establishment (in contrast to sprayed systems where this reduced herbicide efficiency), a better light interception by the crop, and more shading of weeds.

#### What Changes in Untilled Pea Crops?

In untilled spring pea, only the main usual 7–8 variety rules were useful (Table 5E). This resulted in a lower weed-infested yield (0.46–0.54 for the 3.4% best-untilled systems) than in either sprayed (Table 5B) or unsprayed pea (Table 5E). The best branch B1 was based on high-density pea and needed few other management rules, except on wheat varieties and herbicides at the rotation scale. But, the other two branches, with their lower pea densities, needed many other management rules, either at the rotation scale or specifically for the two most frequent crops, wheat, and oilseed rape. In all three branches, herbicide rules were crucial.

#### Do Rotation-Scale Analyses Lead to the Same Conclusions?

If the performance was analyzed at the rotation scale, the pea-parameter rules did not change, here is the example of continuously untilled cropping systems (Table 5F) compared to untilled pea and analysis focusing on pea yield only. The main difference concerned herbicides: these became even more crucial at the scale of the no-till system and/or in the two most frequent crops than when analyzing untilled pea.

#### To Which Extent Ideal Varieties Can Help to Reach Multi-Performance?

When more goals were included in the approach, e.g., reduced herbicide use and improved bee-food offer in addition to yield-loss control, the focus shifted from pea parameters to crop management throughout the rotation (particularly herbicide options, details in section F of the Supplementary Material). Because of the trade-off between biodiversity promotion and harmfulness control, no optimal branches reconciling all these goals could be identified. The best options improved yield (normed yield = 0.38–0.53) and reduced herbicide use (normed = 0.78–1, note that 1 corresponds to zero use) but bee food offer remained stubbornly low (normed = 0.07–0.15). Even the branch with the best bee food, this offer remained low (0.22) and came with a high price in terms of pea yield (0.1).

#### Conclusion

In conclusion, the main pea-parameter rules were the same for all performance goals, management strategies, and analyses scales, stressing the importance of early field occupation and shade response aiming to increase plant height and leaf area. However, further different rules were useful for individual goals, strategies, and scales. Some variety features only fitted to particular systems (e.g., delayed pea emergence is only advantageous in case of spraying and disastrous in unsprayed systems). Fewer parameter rules usually had to be compensated by more management rules. Similarly, if one of the two main weed-control levers, herbicide or tillage, was eliminated, further pea-parameter and/or management rules were needed.

## Discussion

### What Is New?

In the present paper, we combined detailed experimental measurements characterizing varieties with a simulation study to test actual and virtual varieties in virtual field networks in the example of pea, in order to identify ideotypes of varieties and cropping systems for agroecological weed management. This approach was inspired by our previous paper (Colbach et al., 2019) focusing on inter-species instead of intra-species differences, where we already discussed the novelty of this approach as well as its advantages and limits, in terms of variability of investigated situations, use of parameters reflecting intrinsic plant properties that vary little with the environment, and lower risk of confusion effects.

Here, we went several steps further to (1) include all species parameters instead of focusing on plant morphology and shading response, (2) shift from the species to the variety scale, and to (3) identify relevant crop parameters and, particularly, parameter combinations not only for a given production goal but also for contrasting cropping-system types. The present study differed in another key point: it not only aimed to determine ideotypes of varieties but also (4) ideotypes of crop management plans and cropping systems. Working at the cropping-system scale is particularly novel. Though ecophysiologists design ideotypes for different agronomic and pedoclimatic conditions and they even use sowing strategies (e.g., density, interrow width) to achieve ideal plant morphology via morphological plasticity, they usually disregard multiannual effects (Martre et al., 2015).

In terms of methodology, we shifted from real-life cropping systems and crops in our previous paper (Colbach et al., 2019) to virtual (randomized) systems and varieties in the present study, to explore a larger range of parameters/techniques and their combinations. The Latin Hypercube Sampling (LHS) plan for the simulation plan was particularly appropriate for this objective, as it was shown here to both decorrelate management variables (thus reducing the risk of confusing effects) and respect biological correlations among variety characteristics (thus avoiding biological nonsense).

The conclusion of our previous study comparing different species was very similar (Colbach et al., 2019). In addition to “generalist winners” (resulting from the main 7–8 rules identified in all spring-pea cases of Table 5), the present study also searched for ideotypes fine-tuned to individual situations (e.g., with or without herbicides) and variety types (spring vs. winter). Though the main parameter rules remained the same, the “specialized winners” greatly varied, in terms of relevant parameters and parameter combinations, parameter thresholds, and impacts on crop yield and weed (dis)services. Notably, combinations of minor parameters could be more efficient than a single optimum dominant one, particularly if combined with adapted crop management. This makes work more difficult, both for crop breeders (who need to produce contrasting varieties) and farmers (who need to identify the best variety for their production context).

### Are the Results Consistent With Previous Studies?

Our results are conditional on the prediction quality of FLORSYS which was shown to be adequate in a previous study (section “Domain of Validity”). In our previous simulation study, we further checked the consistency of simulated yield loss regarding weed and crop species and parameters, concluding that the species rankings and features identified as relevant were consistent with the literature (Colbach et al., 2019). Here, we specifically searched the literature for pea studies (Table 6). Generally, both our experimental and simulation results agree with the few literature reports on pea.

**TABLE 6:** Consistency of present results compared to literature reports.

Literature	Our results

A. What increases pea competitiveness against weeds?	
Plant height, early branching and early leaf area development (McDonald, 2003; Spies et al., 2017), plant height (wheat, Andrew et al., 2015)	Plant height per unit biomass, specific leaf area, leaf biomass ratio

Spring variety with their faster leaf growth (Crozat, 2010) and taller heights	Spring vs. winter varieties, taller varieties (i.e., Kayanne)

Early vigor (wheat, Andrew et al., 2015)	Early emergence and large early leaf area (winter pea and/or unsprayed pea)

Trade-off between yield potential and weed competition (wheat, Andrew et al., 2015)	Low correlation between potential (weed-free) vs. weed-infested yield or yield loss due to weeds

No effect of pea leaf-type on yield loss (McDonald, 2003)	No effect of afila feature

No effect of maturity on yield loss, variable effect of maturity (McDonald, 2003)	No effect on yield loss of earliness of flowering, once a minimum vegetative growth duration is ensured, no effect of flowering earliness

**B. Other observations**	

Higher susceptibility of spring peas to early limiting factors (plant losses, drought…:) (Lecomte et al.)	Variety parameters are more important for spring vs. winter pea, higher variability in performance in spring pea

Potential yield and instability of hr-winter pea > spring pea > Hr-winter pea (Lecomte et al.)	Same ranking with two exceptions: winter-hr Enduro was as bad as winter-Hr pea; winter-Hr DCG0449 was as good as spring pea

Some *afila* genotypes are competitive (Spies et al., 2017) and present rapid soil cover	*afila* vs. Leafy character was irrelevant

Shading response for specific leaf area (mu_SLA) averaged over 8 varieties at pod filling ∼ 0.45 (estimated from Akhter et al., 2009)	mu_SLA at BBCH = 9 for our 7 varieties in [0.45; 0.83], see Figure 1I

Leaf biomass ratio (LBR) at pod filling in [0.10; 0.19] (estimated from Akhter et al., 2009)	LBR for our 7 varieties in [0; 0.60] at BBCH = 9–10, see Figure 1

Shading response for leaf biomass ratio (LBR) at pod filling in [−0.28; 0.47] (estimated from Akhter et al., 2009)	mu_LBR at BBCH = 9 for our 7 varieties in [−0.35; 0.14], see Figure 1I

Weed-infested pea yield in [3.1; 4.6] t/ha, yield loss in [4; 43]%, weed seed production in [0.7; 1.2] t/ha, pea yield (t/ha) = 6.5 – 0.0028 weed yield (t/ha) (calculated from Jacob et al., 2017)	Potential and weed-infested yield of actual pea varieties in [1.9; 7.2] t/ha and [0; 5.9] t/ha respectively, yield loss in [−33; 99]%, weed seed production in [0; 8.5] t/ha, pea yield (t/ha) = 2.7 – 0.0025 weed yield (t/ha) (section **D.5** of the Supplementary Material)
Potential and weed-infested pea yield in [2.5; 2.1] t/ha and [0.01; 1.7] t/ha, respectively, yield loss in [−66; 97]% (McDonald, 2003)	

There were some discrepancies. For instance, the DCG0449 had a potential yield as high as the best spring pea variety in our study whereas field observations report that winter-Hr varieties such as DCG0449 present the lowest yield potential among all pea variety types because of their late maturity which exposes them to end-of-cycle stresses and by a higher susceptibility to diseases and lodging (Lecomte et al., in prep). Conversely, Enduro performed badly here in contrast to field studies which report yield similar to that of the other winter-hr variety, Isard. Our study showed that the genotypes were highly contrasted between and within pea types, and that seasonality (winter vs. spring and Hr vs. hr), leaf morphology (leafy vs. afila), and usage (protein vs. forage) are insufficient to characterize them.

As in field studies, the present simulation study demonstrated the effect of pea parameters on yield and competitiveness against weeds. However, it is difficult to compare these field reports to our pea parameters which were chosen to be independent of location (e.g., plant height per unit biomass in unstressed conditions) whereas most field-measured variables (e.g., plant height) are also the result of environmental conditions. Some mental arithmetic is needed to conclude how far our results are consistent with field reports (Table 6). Some appear contradictory at first glance, such as the higher yields and lower yield losses reported by Jacob et al. (2017). However, their weed infestation was much lower than ours, demonstrated by their lower weed seed production. Despite these differences, the slope linking pea yield and weed seed production was very similar in their experiments and our simulations. Moreover, other field observations reported lower pea yields, and yield loss ranges similar to ours (McDonald, 2003).

### Implications for Farmers and Crop Breeders

The identification of the parameters relevant for yield and weed control is a major stepping-stone toward decision support for farmers. The take-home message for pea varieties in the present study was the importance of early field occupation and shade response, though the exact features (nature and values of relevant parameters) depend on the targeted weed impacts. The next step will screen the existing pea varieties and identify those similar to the ideotypes. However, many of the parameters used here are not routinely measured during breeding (Zhao et al., 2006) and are estimated in controlled conditions (e.g., Gardarin et al., 2010) or on individual plants in garden plots as in section “Parameterizing Contrasting Pea Varieties.” This would mean retesting all varieties with the help of, e.g., high-throughput phenotyping platforms (Jeudy et al., 2016; Brichet et al., 2017).

The actual pea varieties tested here were not very good at controlling weeds. As at the species scale (Colbach et al., 2019), we found again that varieties or parameters that increase potential yield are not necessarily those that minimize weed-caused yield loss. This is often explained by a trade-off between community performance and competitiveness (Denison et al., 2003). But, even though this is a frequently reported antagonism (Sardana et al., 2017), it is not inevitable as shown by recent varietal improvement in rice (Mahajan et al., 2014, 2015). The low weed-control ability of the current pea varieties probably reflects past breeding history where the focus was on potential yield or disease resistance rather than competitiveness against weeds (Tayeh et al., 2015). The present results (particularly the pea-parameter rankings in section E of the Supplementary Material) are a starting point to guide crop breeders toward more competitive pea varieties.

Some features identified here are not consistent with current breeding trends. For instance, a longer crop growth duration tended to improve pea performance here whereas breeding programs currently aim to shorten the pea crop cycle in order to escape to limiting factors of the end of the crop cycle (high temperatures, water deficit), especially for spring and Hr-winter peas, whose cycle is positioned later than hr-winter peas. This points to one of the limits of the present study, which considered water to be non-limiting after plant emergence. Conversely, the present results do not question the current preference for *afila* varieties, which perform better with respect to diseases and lodging, as this parameter was not relevant here for weed regulation.

### From Theory to Practice: What Remains to Be Done?

The pea-parameter rankings of section E of the Supplementary Material and the optimal parameter-value × technique combinations of section F of the Supplementary Material are a first essential step for decision support to both crop breeders and farmers, proposing varieties for agroecological weed management depending on the cropping-system type and performance goal. Or, vice-versa, rules for adapting crop management to a given variety that was chosen, for instance, for protein content rather than for weed control. For practical use, the parameter rankings and decision trees must be transformed into an actual decision support system, which must be specified with future users, notably concerning the way these results should be visually represented in a graphical user interface.

“Packaging” is not the only issue. As mentioned in section “Implications for Farmers and Crop Breeders,” neither the here-tested virtual varieties nor the identified optimal parameter-value × technique combinations scored highly when attempting to achieve both high potential yield and high yield loss control (and even worse when adding other goals). Possibly, we simply missed the “winning” varieties as we only assessed seven actual and 10 virtual ones. To specifically search for these winners, if they exist, or to build a set of trade-off solutions that would be used by stakeholders to choose a compromise according to their constraints, optimization algorithms could be used (Press et al., 2007; Venter, 2010).

Another limitation of the applicability of the present results is that many of our parameters are difficult to measure (particularly those related to plant morphology and shading response) and are not routinely measured by plant breeders (Zhao et al., 2006). A solution would be to link our parameters (which are usually measured in controlled conditions and/or isolated plants) to routinely measured features and, if necessary, propose additional measurements that are easily carried out on dense canopies in field trials during varietal selection and testing. The same approach was already used at the species scale to estimate many of FLORSYS’ most difficult parameters (e.g., Gardarin et al., 2012; Colbach et al., 2020b) but has not yet been attempted at the within-species scale. Recent advances in artificial intelligence and image analysis will also be helpful, such as the correlation established recently between destructive measurements of above-ground biomass and contactless measurements of leaf area (Gée et al., 2021). New techniques for image segmentation (artificial convolutional neural networks) will also be crucial to discriminate different species (Bateman et al., 2020).

The present study focused on crop-weed competition for the light which is generally the main resource for which crops and weeds compete in conventional cropping systems (Wilson and Tilman, 1993; Perry et al., 2003). But with the current limitation of nitrogen fertilizer for environmental reasons (Swarbreck et al., 2019), crop-weed competition for nitrogen will become more prevalent. Moreover, as a nitrogen-fixating legume crop, pea will have a major advantage over weeds, at least in temperate cropping systems where there are no *Fabaceae* weeds. Such a study will soon be possible with the recent introduction of crop-weed competition for nitrogen into FLORSYS (Moreau et al., 2021).

Finally, the present simulation study was run in a single region, albeit with different weather series and weed floras. As variety performance has been shown to vary among regions and years (McDonald, 2003; Jacob et al., 2017), our study must now be repeated in other pedoclimates to adapt the selection and farming guidelines. These will probably vary, as they do here among cropping systems.

## Conclusion

The present sensitivity analysis of pea production and biological weed regulation to pea variety and crop management identified the key parameters that drive potential yield and competitivity against weeds in pea, depending on variety type and cropping system. These are pointers for selecting pea varieties in agroecological cropping systems aiming to regulate weeds by biological interactions. We also produced rules to guide farmers (1) to choose the best pea variety, depending on the production goal and the cropping system, and (2) to adapt crop management to a given pea variety and production goal. The present methodology can be applied to identify ideotypes for weed management in other crops or even crop mixtures. Further research is needed to resolve the trade-off between pea parameters that promote potential yield and those driving weed suppression and to link model parameters to variables routinely measured during crop breeding.

## Data Availability Statement

The raw data supporting the conclusions of this article will be made available by the authors, without undue reservation.

## Author Contributions

NC and DM designed and led the research. EF, LL, and FS carried out the experiments and measurements. AK and CL provided expert advice. CG performed the image analysis. NC and JV ran the simulations and analyzed the data. TM provided expert advice. NC and DM wrote the draft manuscript with the help of CL. All authors contributed to the article and approved the submitted version.

## Conflict of Interest

The authors declare that the research was conducted in the absence of any commercial or financial relationships that could be construed as a potential conflict of interest.

## Publisher’s Note

All claims expressed in this article are solely those of the authors and do not necessarily represent those of their affiliated organizations, or those of the publisher, the editors and the reviewers. Any product that may be evaluated in this article, or claim that may be made by its manufacturer, is not guaranteed or endorsed by the publisher.

## Footnotes

1
https://agreste.agriculture.gouv.fr/
