# Supplementary material for: Tracking Ideal Varieties and Cropping Techniques for Agroecological Weed Management: A Simulation-Based Study on Pea
Source: Front Plant Sci. 2022 Apr 4;13:809056. doi: 10.3389/fpls.2022.809056 (PMC9014269; doi:10.3389/fpls.2022.809056)
Supplement: Supplementary file 2 [file Data_Sheet_2.PDF]

Supplementary material

Tracking ideal varieties and cropping techniques for agroecological weed management: a simulation-based study on pea

Nathalie Colbach, Emeline Felten, Christelle Gée, Antony Klein, Laura Laurenzel, Christophe Lecomte, Florence Strbik, Jean Villerd, Delphine Moreau

Agroécologie, INRAE, Institut Agro, Univ. Bourgogne Franche-Comté, F_21000 Dijon
Nathalie.Colbach@inrae.fr

* Address for correspondence: Nathalie Colbach
INRAE, UMR1347 Agroécologie
BP 86510, 17 rue Sully
21065 Dijon Cedex, France
Nathalie.Colbach@inrae.fr

Content

Supplementary material Tracking ideal varieties and cropping techniques for agroecological weed management: a simulation-based study on pea 1
A. Additional details on the FLORSYS model 2
A.1 The annual life-cycle 2
A.2 Parameterized species 3
A.3 The effect of management practices 6
A.4 Indicators of weed impact on crop production and biodiversity 7
A.5 List of species parameters and origin for pea varieties 9
B. Additional information on the garden plot experiments 9
B.1 The biological meaning of the measured species/variety parameters 9
B.2 How to estimate plasticity/morphology parameters in garden plots 16
C. Additional results from the garden plot experiments 24
C.1 Parameter values of plant morphology and shading response 24
C.2 Correlations among morphology and shading-response parameters 25
C.3 Correlations among all variety parameters 27
D. Additional results from the virtual experiments (simulations) 28
D.1 Preliminary simulation study aiming to link weed species to weed impact 28
D.2 Further details on the simulation plan 30
D.3 Further explanations on Classification and Regression Trees (CART) 32
D.4 Variation in simulated output 34
D.5 Variation in yield and weed (dis)services across situations 35
E. Pea parameters and management techniques driving weed impact 37
F. CART for weed-impact indicators in different situations 37
G. Acknowledgements 38
H. References 38

A. Additional details on the FLORSYS model

A.1 The annual life-cycle

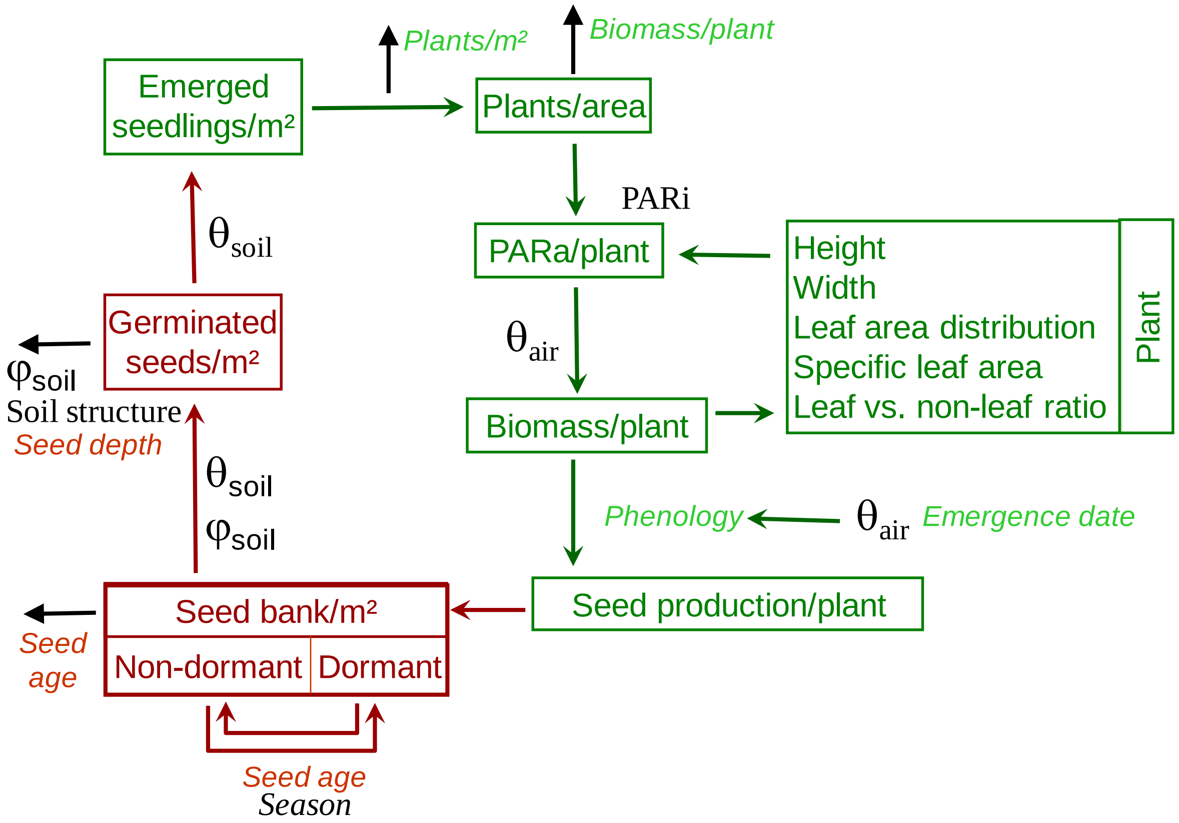

Figure 1. Life-stages (plants/m²) of annual weeds simulated in FLORSYS (Gardarin *et al.*, 2012; Munier-Jolain *et al.*, 2013; Colbach *et al.*, 2014b; Munier-Jolain *et al.*, 2014) with the effects of weed state variables (e.g. *Plants/m²*, *Seed age*), soil conditions (e.g. θ_{soil}) and daily weather variables (e.g. PARi). All variables are calculated daily. Black arrows (⟶) indicate losses through mortality (Nathalie Colbach)

A.2 Parameterized species

Table 1. The 26 annual weed species included in FLORSYS

Family	Species	EPPO code	Seed mass (mg)[§]
Poaceae	*Alopecurus myosuroides*	ALOMY	2.3
	Avena fatua	AVEFA	18.5
	Digitaria sanguinalis	DIGSA	0.63
	Echinochloa crus-galli	ECHCG	2.24
	Lolium multiflorum	LOLMU	2.05
	Panicum miliaceum	PANMI	4.3
	Poa annua	POAAN	0.3
	Setaria viridis	SETVI	1.4
Amaranthaceae	*Amaranthus retroflexus*	AMARE	0.38
	Ambrosia artemisiifolia	AMBEL	4.59
	Chenopodium album	CHEAL	0.56
Asteraceae	*Matricaria perforata*	MATIN	0.27
	Senecio vulgaris	SENVU	0.26
	Sonchus asper	SONAS	0.3
Brassicaceae	*Capsella bursa-pastoris*	CAPBP	0.14
Caryophyllaceae	*Stellaria media*	STEME	0.4
Euphorbiaceae	*Mercurialis annua*	MERAN	1.87
Geraniaceae	*Geranium dissectum*	GERDI	2.12
Malvaceae	*Abutilon theophrasti*	ABUTH	8.12
Plantaginaceae	*Veronica hederifolia*	VERHE	3.52
	Veronica persica	VERPE	0.67
Polygonaceae	*Fallopia convolvulus*	POLCO	6.52
	Polygonum aviculare	POLAV	1.52
	Polygonum persicaria	POLPE	1.9
Rubiaceae	*Galium aparine*	GALAP	7.37
Solanaceae	*Datura stramonium*	DATST	7.2
	Solanum nigrum	SOLNI	0.8

[§] Dry mass per seed

Table 2. The 30 cash and cover crop species and varieties included in FLORSYS

Family	Species	Variety	EPPO code	Seed mass (mg)[§]
Poaceae	*Avena strigosa*	Pratex	AVESG	18.11
	Festuca rubra	Greenlight	FESRU	0.86
	Lolium multiflorum		LOLMU	2.9
	Sorghum bicolor		SORVU	23.0
	Triticum aestivum	Caphorn	TRZAX	42.1
		Cézanne	TRZAX	45.5
		Orvantis	TRZAX	42.1
	xTriticosecale	Matinal	TTLSS	43.6
	Zea mays		ZEAMX	252
Asteraceae	*Guizotia abyssinica*	Azofix	GUIAB	2.51
	Helianthus annuus		HELAN	41.1
Boraginaceae	*Phacelia tanacetifolia*	Angelia	PHCTA	1.98
Brassicaceae	*Brassica napus*		BRSNN	4.4
	Camelina sativa		CMASA	1.35
	Raphanus sativus	Cardinal	RAPSR	12.1
	Sinapis alba		SINAL	6.5
Chenopodiaceae	*Beta vulgaris*		BEAVX	2.8
Fabaceae	*Glycine max*		GLXMA	185
	Lathyrus sativus	N-fix	LTHSA	162
	Lens culinaris	Anicia	LENCU	31.0
	Lens nigricans	Lentifix	LENNI	17.1
	Lotus corniculatus	Leo	LOTCO	1.13
	Medicago lupulina	Virgo	MEDLU	1.71
	Medicago sativa	Galaxy	MEDSA	2.00
	Phaseolus vulgaris	Booster	PHSVX	79
	Pisum sativum	886/1	PIBSX	131
		Cameor	PIBSX	157
		China	PIBSX	153
		DCG0449	PIBSX	102
		Enduro	PIBSX	187
		Isard	PIBSX	153
		Kayanne	PIBSX	183
	Trifolium alexandrinum	Tabor	TRFAL	3.64
	Trifolium pratense	Trevviso	TRFPR	2.20
	Trifolium repens	Aberdai	TRFRE	0.66
	Trigonella foenum-graecum	Fenusol	TRKFG	16.9
	Vicia faba	Diana	VICFX	270
		Gladice	VICFX	426
	Vicia sativa	Nacre	VICSA	50.4
Linaceae	*Linum usitatissimum*		LIUUT	7.4

[§] Dry mass per seed

Table 3. Major FLORSYS species traits and parameters and their range of variation

Trait/parameter	Unit	Mean	Min	Max
Relative growth rate	cm^2 °C^{-1} day^{-1}	0.020	0.011	0.046
Initial leaf area (ILA)	cm^2	0.20	0.013	0.70
Variation coefficient of ILA	cm^2 cm-^{2}	0.24	0.0061	1.27
Base temperature for growth and development	°C	4.4	0	12.0
Harvest index	g g^{-1}	0.29	0.010	0.86
Shape parameter for harvest index	No unit	1.01	0.77	1.40
Climbing	{yes, no}	12% Yes	No	Yes
Maximum plant height	cm	95	30	200
Maximum plant width	cm	106	20	200
Seed Weight	mg	3.07	0.14	18.50
Seed lipid content	g g^{-1}	0.16	0.030	0.47
Seed coat thickness	µm	65	10	231
Seed area	mm^2 mg^{-1}	3.91	0.21	17.50
Seed shape index	mm^2 mm^{-2}	0.21	0.050	0.47
Base temperature for germination	°C	4.3	0	11.5
Base soil water potential for germination	MPa	-1.12	-3.31	-0.45
Emergence season onset	Julian day	158	60	280
Emergence onset in spring	Julian day	85	20	140
End of emergence season	Julian day	177	70	310
Monocotyledonous species	{yes, no}	24% Yes	No	Yes
Specific leaf area (SLA)	cm^2 g^{-1}	189	89	301
Sensitivity of SLA to shade	No unit	0.61	0.17	1.20
Leaf biomass vs. total biomass ratio (LBR)	g g^{-1}	0.70	0.55	0.84
Sensitivity of LBR to shade	No unit	0.051	-0.31	0.43
Specific plant height or height per biomass (HM)	cm g^{-1}	38	8	136
Shape parameter for HM	No unit	0.33	0.10	0.59
Sensitivity of HM to shade	No unit	0.58	-0.11	1.19
Specific plant width or width per biomass (WM)	cm g^{-1}	116	14	1531
Shape parameter for WM	No unit	0.41	0.20	0.91
Sensitivity of WM to shade	No unit	0.35	-0.040	0.84
Median relative leaf area height (RLH)	cm cm^{-1}	0.49	0.37	0.67
Shape parameter for RLH	No unit	2.71	1.64	4.14
Sensitivity of RLH to shade	No unit	0.018	-0.54	0.62
Stimulating parasite germination	{yes, no}	40% Yes	No	Yes
Allowing parasite attachment	{yes, no}	36% Yes	No	Yes

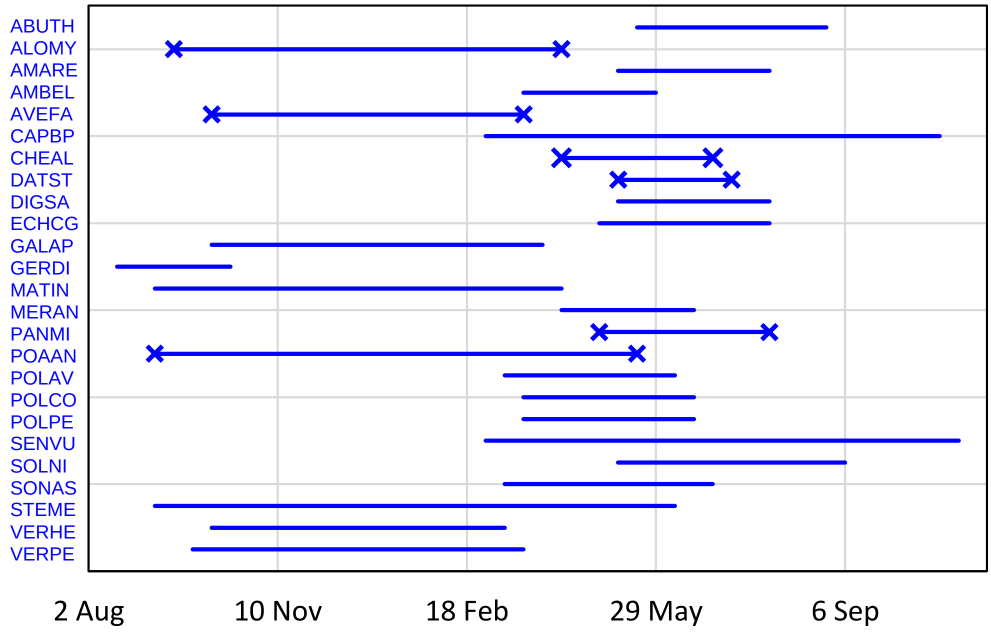

Figure 2. Potential emergence seasons of the 25 weed species (indicated by their EPPO code) included in FLORSYS. Grass weeds are indicated by crosses. (Nathalie Colbach CC BY-NC-ND 2016).

A.3 The effect of management practices

Table 4. Effects of cropping system components on the weed life-cycle (density and timing of stages) as simulated by FLORSYS (Gardarin *et al.*, 2012; Munier-Jolain *et al.*, 2013; Colbach *et al.*, 2014a; Colbach *et al.*, 2014b; Munier-Jolain *et al.*, 2014). The effect of other management techniques (e.g. nitrogen) is not yet implemented in FLORSYS.

Cropping system component (crops and management techniques)	Intermediate effect	Effect on weeds
Tillage (including post-sowing mechanical weeding)	Soil structure<br>Soil movements = f(soil structure)	Soil compaction increases mortality of germinated seeds<br>Seed burial decreases germination and increases pre-emergent mortality due to insufficient seed reserve<br>Seeds on soil surface germinate badly because of insufficient seed-soil contact<br>Germinated seeds close to soil surface often die because the top soil dries faster<br>Exposure of imbibed seeds to light if inverting tool<br>Triggering of germination flush if the soil is tilled in moist conditions<br>Destruction of germinated seeds, seedlings and plants; addition of newly produced seeds to seed bank if mature plants are killed
Crop species and variety (including undersown, associated and temporary crops)	Choice of cultivation techniques<br>Sowing season<br>Light availability in canopy	See effects of techniques<br>Selects weed species that are non-dormant at sowing season<br>Shading reduces photosynthesis and thus biomass accumulation and results in etiolation
Sowing date	Crop emergence date<br>Date of last tillage	The earlier the weed seedlings emerge relative to the crop, the better they survive<br>The later the last tillage, the more weed seeds have germinated already and are killed by the tillage
Sowing density	Reduces light availability in canopy	Shading reduces photosynthesis and thus biomass accumulation and results in etiolation
Sowing pattern	Variability in light availability in canopy	Irregular sowing leads to canopy gaps where weeds grow and reproduce better
Herbicides	Efficiency = f(active ingredient, technicity)<br>Efficiency decreases with canopy density, seed depth (for root-entering herbicides) and weed stage	Foliar herbicides kill emerged plants, root-entering herbicides kill unemerged and emerged plants whose seeds are close to soil surface, pseudo-root herbicides (entering via the shoot tip) kill emerging seedlings; root-entering and pseudo-root herbicides persist and act during several days.<br>Addition of newly produced seeds to seed bank if mature plants are killed and germination flush if soil is moist
Mowing & harvesting operations		Cuts plants and reduces biomass; the older the plants at mowing and the less biomass remain, the more plants die; addition of newly produced seeds to seed bank if mature plants are killed and germination flush if soil is moist
Manure	Adds layer on soil surface	Improves germination of surface seeds, slightly decreases germination and emergence of buried seeds<br>Adds seeds to soil seed bank
Irrigation	Increases soil moisture and water potential	Triggers weed seed germination if applied after drought<br>Makes germination and emergence faster<br>Interacts with techniques whose effects depends on soil moisture (tillage, mechanical weeding, soil compaction)
All (except irrigation)	Increase soil compaction via wheel traffic	Increases mortality of germinated seeds

A.4 Indicators of weed impact on crop production and biodiversity

Table 1. Synopsis of the indicators calculated from weed flora outputs predicted by the FLORSYS model. Indicators are calculated for each cropping season, i.e. from harvest of the previous crop to harvest of current crop (Mézière *et al.*, 2015; Colbach *et al.*, 2017).

A. Weed harmfulness for crop production

Indicator	Description	Equation	Variables
Crop production			
Yield loss	Crop yield loss due to crop:weed competition for light (%)	$100 \cdot (Y_0 - Y)/Y_0$	Y and Y_0=crop yield in weedy and weed-free simulations with the same cropping system ($g{\cdot}m^{-2}$)
Harvest pollution	Pollution of crop seed harvest by weed seeds and plant fragments (no unit), not calculated for grass crops, root crops and silage maize.	$\log_{10}\left[\frac{\sum_{i=1}^{S}(\alpha_{ic}\cdot S_i+\beta_{ic}\cdot B_i)}{Y}+0.0001\right]+4$	S_i, B_i =seed biomass and weed biomass produced by plants taller than harvester cutter bar ($g{\cdot}m^{-2}$)<br>B_c=crop biomass at harvest ($g.m^{-2}$)<br>Y=crop yield<br>α_{ic}, β_{ic} = coefficients of harvest pollution by weed seeds or green biomass
Production activity			
Harvesting difficulty	Technical problems induced by weeds at harvest.	$\log_{10}\left[\frac{\sum_{i=1}^{S}B_i}{B_c}+0.0001\right]+4$	B_i, B_c =fresh weed biomass and crop biomass taller than harvester cutter bar at harvest ($g{\cdot}m^{-2}$)
Farmer's field perception			
Field infestation	Daily weed biomass in the field averaged sowing date to harvest date ($t.ha^{-1}.day^{-1}$)	$\frac{\sum_{d=1}^{D}\sum_{i=1}^{S}B_{id}}{D}$	B_{id}=fresh weed biomass of i on day d ($t{\cdot}ha^{-1}$)<br>D=number of days
Pest increase due to weeds			
Disease risk	Additional crop yield loss due to increase in take-all disease in cereals caused by grass weeds (%)	$AD = YLD - YLD_0$	YLD and YLD_0 = crop yield loss due to disease in respectively weedy and weed-free simulations of the same cropping system. Output from TAKEALLSYS linked to FLORSYS with an interaction model (Mézière *et al.*, 2013)
Parasite risk	Risk of crop infection by parasitic plant *Phelipanche ramosa* due to weeds	$-\alpha \cdot I_{seed\_bank\_decline}$<br>$+ \beta \cdot I_{increase\_crop\_infection}$<br>$+ \gamma \cdot I_{tot\_stim} \cdot I_{repro}$	$I_{seed\_bank\_decline}$ is the risk of total parasite germination stimulated by weeds and is estimated from above-ground biomass of weeds that belong to parasite-stimulating species and that have not yet flowered, averaged over cultural campaign<br>$I_{increase\_crop\_infection}$ is the risk of parasite germination stimulated by weeds during host crops and is estimated from above-ground biomass of weed plants that belong to parasite-stimulating species and have not yet flowered, averaged over host crop season<br>$I_{parasite\_reproduction}$ is the product of the risk of parasite germination stimulated by weeds, and the risk of parasite seed production of weeds, the latter being estimated from above-ground biomass of weeds that belong to parasite-susceptible species and reached maturity<br>α, β and γ are positive parameters
Proxy for future weed harmfulness			
Future harmfulness	Risk that the current weed flora will impact future crop production	$\sum_{d=1}^{D}\sum_{i=1}^{S}SP_{id}$	SP_{id} = seeds produced by plants of species i on day d, between crop sowing and crop harvest

Weed species i ∈ {1,...S} with S the species richness. For indicators with log in the formula, 0.0001 was added to account for nil values. A +4 constant was added to indicators using a $\log_{10}(y+0.0001)$ *transformation to ensure that indicator values* ≥ 0.

B. Weed-related biodiversity indicators

Indicator		Description	Equation	Variables
Plant biodiversity				
Species richness	S	Number of weed species present during the cropping season ∈ [0, 25]		
Species equitability	E	Pielou's equitability (ratio of Shannon index of the community vs. Shannon maximum, i.e. if all the species of communities present the same abundance), varying between [0,1]	$E = H'/H_{max}$ with $H' = -\sum_{i=1}^{S} \frac{n_i}{N} \cdot \log_2(\frac{n_i}{N})$ and $H_{max} = \log_2 S$ $E = 0$ if $N=0$	n_i= daily number of plants of species i averaged over season (plants·m^{-2}) N= total daily number of weed plants averaged over season (plants·m^{-2})
Trophic resources for non-pest biodiversity				
Bird resource	B	Weed seeds important for farmland bird diet and present on soil surface between 1 October and 15 March	$B = \frac{1}{D}\sum_{d=1}^{D}(\log_{10}[\sum_{i=1}^{S}(s_{id} \cdot \gamma_i) + 0.0001] + 4)$	s_{id}=seed density on soil surface (seeds·m^{-2}) D = days γ_i=importance in the diet farmland birds (Wilson et al., 1999; Marshall et al., 2003); $\gamma \in \{1,2,3,4\}$.
Insect resource	I	Lipid-rich weed seeds for feeding granivore carabids, present on soil surface between 1 April and 1 October	$I = \frac{1}{D}\sum_{d=1}^{D}(\log_{10}[\sum_{i=1}^{S}(s_{id} \cdot \delta_i) + 0.0001] + 4)$	s_{id}=seed density of species i on soil surface on day d (seeds·m^{-2}) D = days δ_i=seed lipid content (%) of species i (Gardarin et al., 2011)
Pollinator resource	P	Weed flowers for feeding honey bees and open from 1 March and 1 November	$P = \frac{1}{D}\sum_{d=1}^{D}(\log_{10}[\sum_{i=1}^{S}(f_{id} \cdot \eta_i) + 0.0001] + 4)$	f_{id}=flowering plant density (plants·m^{-2}) D = days η_i=pollination value (Ricou et al., 2014); $\eta \in \{1, 2, ..., 7\}$.

A.5 List of species parameters and origin for pea varieties

Table 5. List and estimation method of parameters describing pea varieties in FLORSYS. A more detailed list of parameters can be found in section **E6** online.

Type of parameter	Number	Method of estimation	Reference
Pre-emergent temperature and water requirements	2	Petri dish experiments	(Raveneau *et al.*, 2011; Varela Nicola, 2017)
Germination dynamics	5	Petri dish experiments	(Raveneau *et al.*, 2011; Varela Nicola, 2017)
Pre-emergent growth	6	Pot experiments and function relationships	(Gardarin *et al.*, 2016)
Establishment	3	Greenhouse experiments and functional relationships	(Colbach *et al.*, 2020)
Effect of soil structure on germination and pre-emergent growth	2+7	Pot experiments and functional relationships	(Gardarin *et al.*, 2010; Gardarin *et al.*, 2016)
Base temperature and duration of development stages	1+16	STICS, greenhouse, garden-plot and field experiments	(Brisson *et al.*, 2009; Tayeh *et al.*, 2015; Colbach *et al.*, 2020), Section 2.2.2 in manuscript
Potential morphology and shade response per BBCH stage	2+(8+5)×11	Garden-plot experiments	(Colbach *et al.*, 2020), Section 2.2.2 in manuscript
Root system growth and structure	13	Greenhouse & field experiments, Archisimple simulations	(Pointurier *et al.*, 2021)
Temperature thresholds for photosynthesis	4	STICS	(Brisson *et al.*, 2009)
Temperature thresholds for frost damage per growth period	3×4	STICS and expertise from Christophe Lecomte	(Lecomte *et al.*, 2003; Brisson *et al.*, 2009; Castel *et al.*, 2017)
Light interception and use	2	STICS	
Seed weight	1	Field experiments	(Colbach *et al.*, 2020), Section 2.2.2 in manuscript
Harvest index	2	Field experiments	(Tayeh *et al.*, 2015), field experiments during Peamust projet (30-40 fields per variety, Lecomte, pers comm)
Seed energy content	1	Data base	

B.Additional information on the garden plot experiments

B.1 The biological meaning of the measured species/variety parameters

Most of this section was taken from the supplementary material online of Colbach et al. (2020).

B.1.1 List of species/variety parameters

Table 6. FLORSYS parameters for potential plant morphology and species/variety response to shading, based on (Munier-Jolain *et al.*, 2014)

Parameter name	Relative advance of growth stage at the time of parameter measurement	Unit	Range
A. Potential morphology (morphology variables in unshaded conditions)			
SLA0	Specific Leaf Area (leaf area vs leaf biomass)	$cm^2 \cdot g^{-1}$	[0;∞]
LBR0	Leaf biomass ratio (leaf biomass vs total above-ground biomass)	none	[0;1]
HM0	Specific (allometric) plant height (height vs. total above-ground biomass ratio)	$cm \cdot g^{-1}$	[0;∞]
b_HM	Shape parameter = Sensitivity of plant height to above-ground plant biomass (0=none)	none	]0;∞]
WM0	Specific (allometric) plant width (width vs. total above-ground biomass ratio)	$cm \cdot g^{-1}$	[0;∞]
b_WM	Shape parameter = Sensitivity of plant width to above-ground plant biomass (0=none)	none	]0;∞]
RLH0	Median relative leaf height (relative plant height below which 50% of leaf area are located)	$cm\ cm^{-1}$	[0;1]
b_RLH	Shape parameter for leaf distribution along plant height	none	]0;∞]
B. Response to shading (variation in morphology variables with shading intensity)			
SLA_mu	Response of specific leaf area to shading	none	[-∞;∞]
LBR_mu	Response of leaf biomass ratio to shading	none	[-∞;∞]
HM_mu	Response of specific height to shading	none	[-∞;∞]
WM_mu	Response of specific width to shading	none	[-∞;∞]
RLH_mu	Response of median relative leaf height to shading	none	[-∞;∞]

B.1.2 Principle for estimating effects of shading by neighbours

If seedlings emerge under an existing canopy, they are shaded by older and taller plants. The larger their leaf area is, the more they self-shade, i.e. leaves at the bottom of the plant are shaded by leaves at the top of the plant. The growth of each plant now not only depends on the plant's leaf area but also on how much light reaches this leaf area, and thus how the plant's leaf is located in space relative to other plants.

Munier-Jolain et al (2014) proposed to describe plant morphology as a series of variables describing plant volume and leaf area distribution inside this volume. Each variable could be predicted from a parameter describing the potential plant morphology in unshaded conditions, as well as the response of the variables in case of shading (Table 6). This principle was formalized as follows:

[1] $Variable_{ps} = \textbf{potential value}_s \cdot exp(\textbf{mu}_s \cdot shading\ index_p)$

Where $Variable_{ps}$ is the variable value for plant p of species or variety s, ***potential value$_s$*** is the potential value of species/variety s in unshaded condition, ***mu$_s$*** is the response of species/variety s to shading for the variable, and $shading\ index_p$ is the shading of plant p since it emerged. ***potential value$_s$*** and ***mu$_s$*** are parameters that depend on the species/variety but also on plant stage. The shading index of plant p on day d is the average shading perceived by the plant since its emergence, with recent shading having more effect than earlier shading:

[2] $$Shading\ index_{pd} = \frac{\sum_{d'=0}^{d} (d' \cdot S_{pd'})}{\sum_{d'=0}^{d} d'}$$

Where $S_{pd'}$ is the shading received by plant p on each day d' from emergence to day d. For details on how this was calculated in canopies in field experiments, see Munier-Jolain et al (2014). For details on how this was calculated for isolated plants in our garden plots, see section B.2.2.5.

The following sections explain the biological significance of the parameters listed in Table 6.

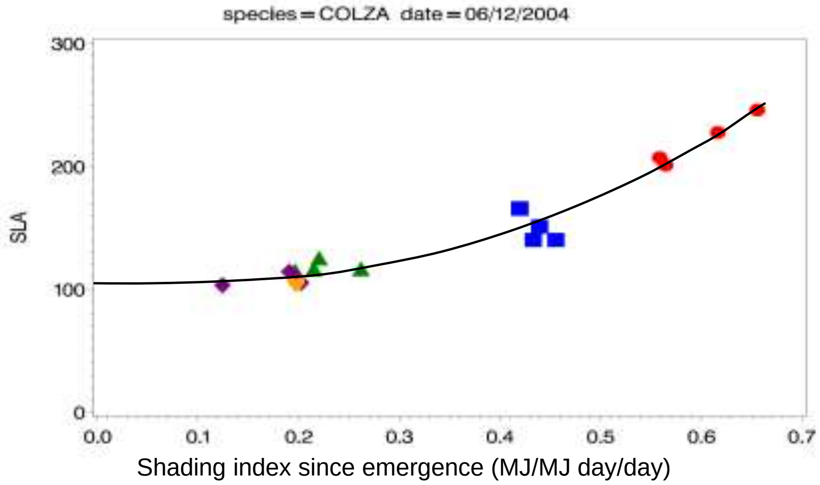

Figure 3. Variation in specific leaf area (SLA) of oilseed rape in December 2014 depending on shading (SI) since plant emergence (Munier-Jolain *et al.*, 2014). Line shows fitted non-linear equation *SLA = 107 exp(1.07 SI)* and symbols are observations from different shading conditions (Nathalie Colbach 2014)

B.1.3 Morphological variables in unshaded conditions

B.1.3.1 Specific leaf area SLA

The specific leaf area (SLA) is the efficiency for producing a large leaf area from a given leaf biomass. It is measured here from the total leaf area of the plant relative to its total leaf biomass, including petioles. A high SLA indicates thin large leaves, a low SLA means thicker smaller leaves (Figure 4).

High SLA	Low SLA
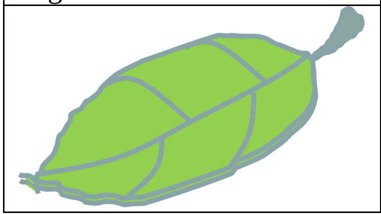	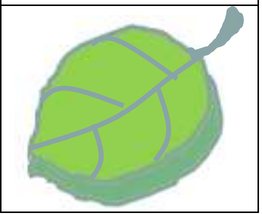

Figure 4. Schematic representation of leaves with contrasting specific leaf areas (Nathalie Colbach).

B.1.3.2 Leaf biomass ratio LBR

The leaf biomass ratio (LBR) is the part of the above-ground biomass that the plant attributes to leaves. It is measured here as the ratio of the total leaf biomass (including petioles) vs the total above-ground biomass. A high LBR indicates a leafy plant, a low LBR a stemmy plant (if flowering and seed production have not yet started) (Figure 5).

High LBR	Low LBR
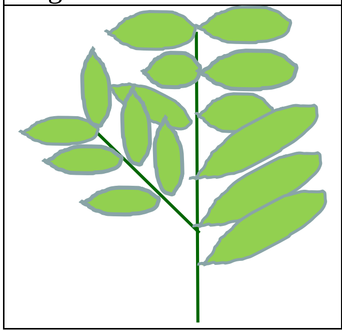	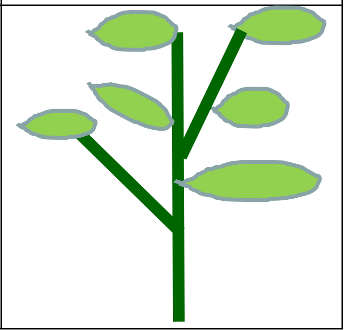

Figure 5. Schematic representation of plants with contrasting leaf biomass ratios (Nathalie Colbach).

B.1.3.3 Specific plant height HM and its shape parameter b_HM

The specific plant height (HM) is the plant height to the above-ground biomass. It is estimated by fitting a linear regression to $\log_n$-transformed plant height vs $\log_n$-transformed above-ground biomass (Figure 6). Specific plant height HM is the exp-transformed constant, the slope is the shape parameter b_HM. The shape of the equation was chosen by Munier-Jolain et al (Munier-Jolain *et al.*, 2014) who analysed plant morphology in different shading conditions over time. Here, we only worked two shading conditions (unshaded and highly shaded).

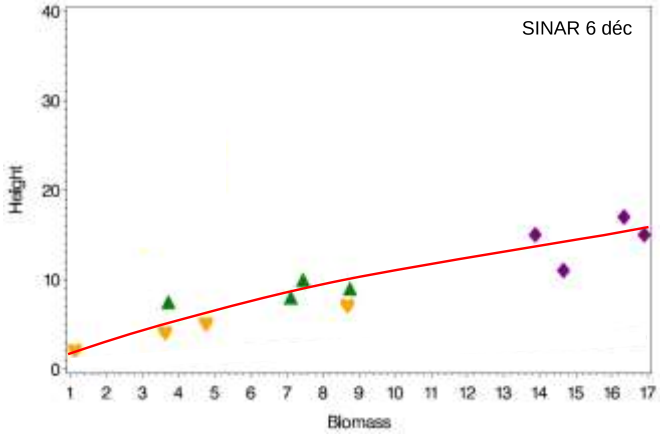

Figure 6. Fitting a linear regression $\log_n$(height, in cm) = a + b $\log_n$(biomass, in g/plant) to plant height vs above-ground plant biomass for plants growing in unshaded conditions. Example of *Sinapis arvensis* on December 6 (Munier-Jolain *et al.*, 2014). Specific plant height HM (cm/g) is exp(a), shape parameter b_HM (no unit) is b. The different symbols represent different shading conditions (see Munier-Jolain et al for details) (Nathalie Colbach 2014)

B.1.3.4 Specific plant height HM

The higher HM, the taller the plants are for a given biomass (Figure 7).

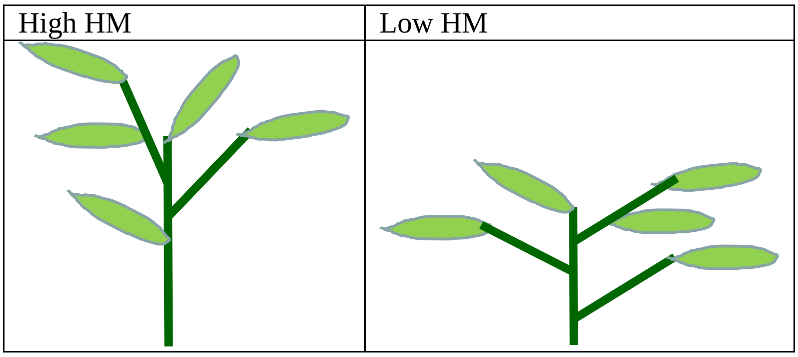

Figure 7. Schematic representation of plants with contrasting specific plant heights (Nathalie Colbach).

B.1.3.5 Shape parameter for specific plant height b_HM

The shape parameter b_HM determines the difference in height efficiency between light and heavy plants. The lower b_HM, the more efficient light plants are compared to heavy plants. If b_HM = 1, plants produce the same height relatively to a given biomass (Figure 8). If b_HM < 1, light plants produce more height relative to their biomass than heavy plants. Or, in other words, if b_HM < 1, light and heavy plants can have the same height. b_HM is never > 1.

b_HM = 1	b_HM < 1
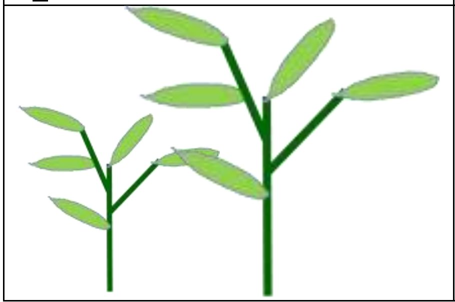	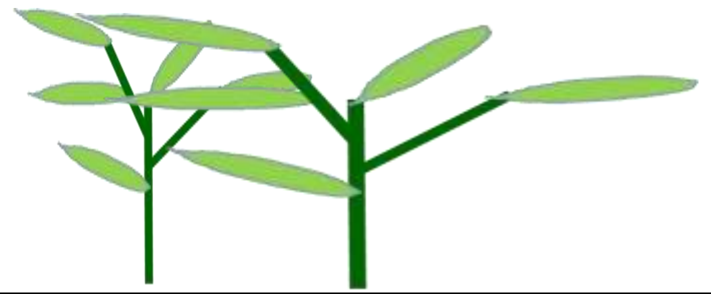

Figure 8. Schematic representation comparing light and heavy plants of species or varieties with contrasting shape parameters for specific plant heights (Nathalie Colbach CC BY NC SA).

B.1.3.6 Specific plant width WM and its shape parameter b_WM

The principles are the same as for specific plant height (section B.1.3.3).

B.1.3.7 Leaf area distribution along plant height (RLH, b_RLH)

B.1.3.8 Median relative leaf area height RLH

Median relative leaf area height RLH is the relative plant height below which 50% of the plant's leaf area are located (Figure 9).

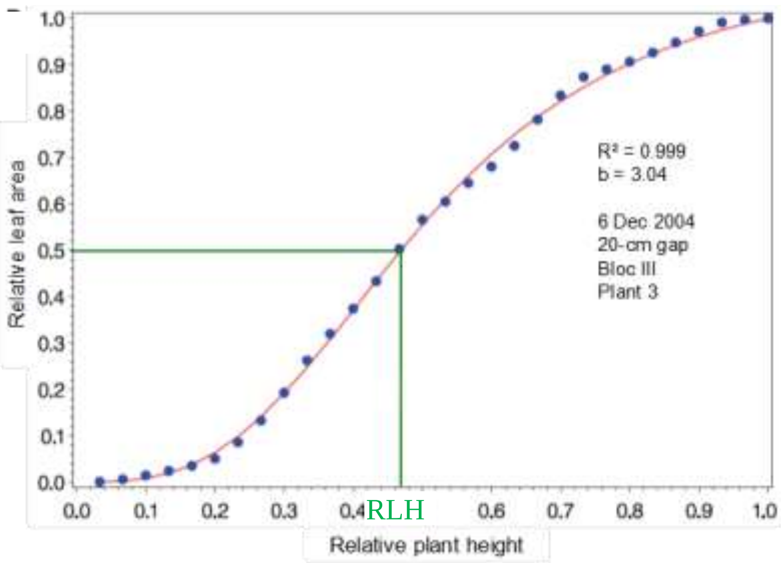

Figure 9. Distribution of relative cumulated leaf area (cm^2/cm^2) along relative plant height (cm/cm). Example of a *Sinapis arvensis* plant in a past field experiment (Munier-Jolain *et al.*, 2014). The non-linear equation (line) fitted to the observations (dots) can be found in section B.2.3.2.4 (Nathalie Colbach CC BY NC SA 2014)

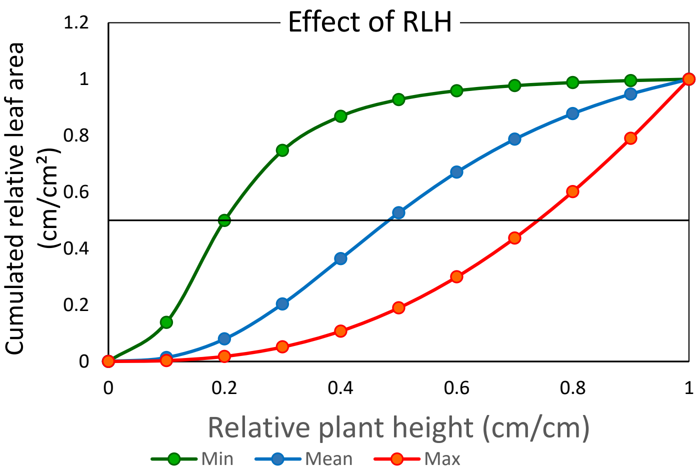

Figure 10. Sensitivity of leaf area distribution along plant height to median relative leaf height RLH. Min, mean and max values of RLH are 0.20, 0.50 and 0.75 (Nathalie Colbach CC BY NC SA)

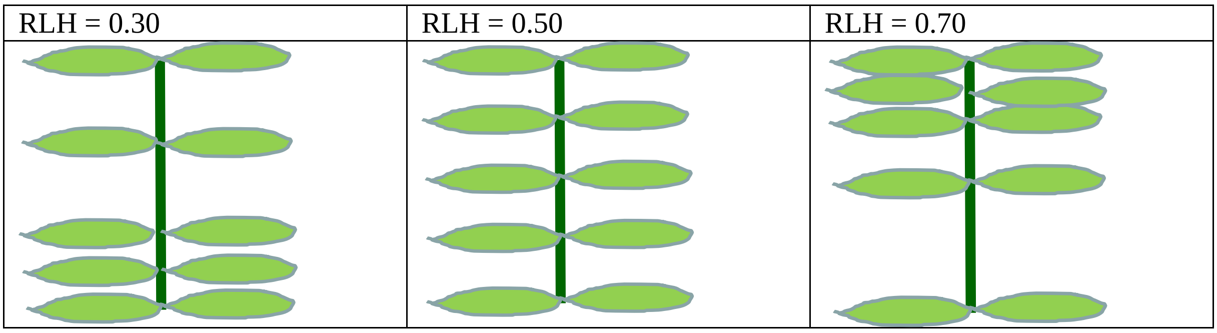

Figure 11. Schematic representation of leaf distribution along plant height for plants with contrasting relative leaf height values (Nathalie Colbach [CC BY-NC-ND]).

B.1.3.9 Shape parameter b_RLH

The significance of the shape parameter b_RLH is more complicated. It is proportional to the part of the leaf area at RLH height. If RLH is 0.50, then b_RLH = 1 means that leaves are distributed uniformly along plant height, b_RLH > 1 means that leaves are toward the middle of the plant, and b_RLH < 1 means that leaves are at the plant's extremities (Figure 12). If RLH is higher (e.g. 0.75), a b_RLH=1 means that half of the leaves are distributed homogeneously in the top quarter of the plant, and the rest in the bottom three quarter of the plant. If RLH is less than 1 (e.g. 0.25), the inverse distribution applies. High and low b_RLH values still indicate leaves concentrated at RLH and the plant extremities, respectively.

In the 52 crop and weed species investigated by Colbach et al. (2020), the plants with the highest b_RLH are also those with the highest RLH values, indicating that plants whose leaves are all at the bottom (picture at the very left of Figure 12) did not occur. b_RLH actually always exceeded 1.

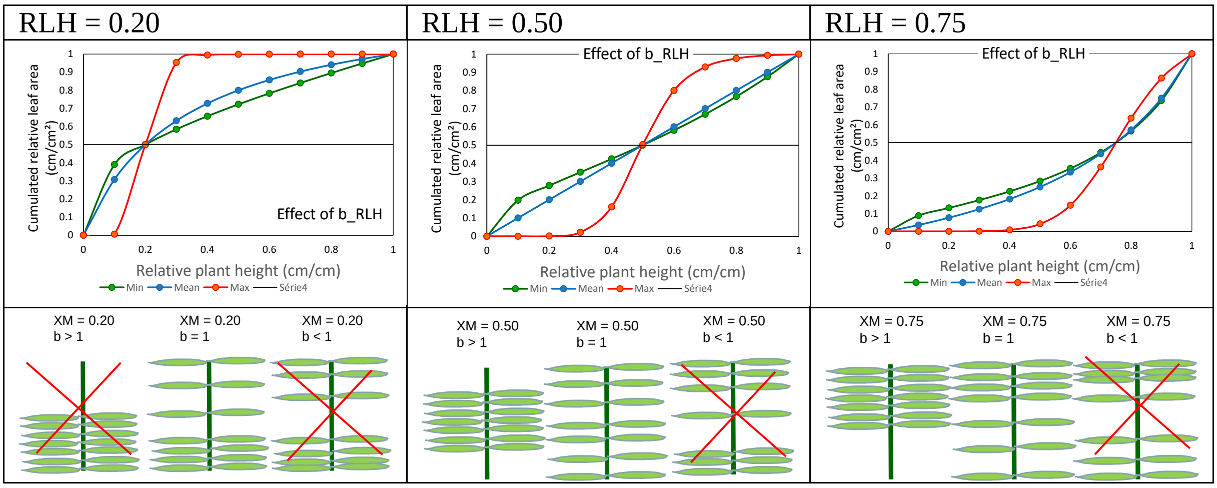

Figure 12. Schematic representation of leaf distribution along plant height for plants with contrasting shape parameter b_RLH values, depending on relative leaf height RLH values. Max, mean and min b_RLH values are those observed on observed in 52 crop and weed species (Colbach *et al.*, 2020). Red crosses indicate the combinations that were not observed in that study (Nathalie Colbach [CC BY-NC-ND] 2020).

B.1.4 Response parameters to shading

Table 7. Schematic representation of parameter response to shading observed in 52 crop and weed species (Colbach *et al.*, 2020)

	**Positive response parameter mu**		
	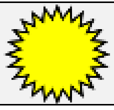	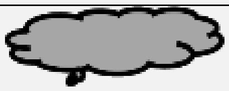	Reason for response to shading
SLA	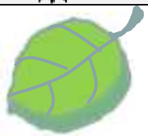	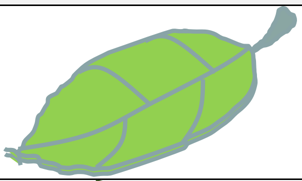	Increase light interception area with thinner larger leaves
LBR	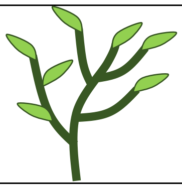	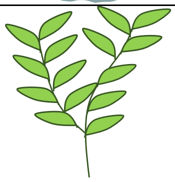	Increase light interception area by increasing leaf biomass to the detriment of stem biomass
HM	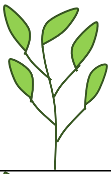	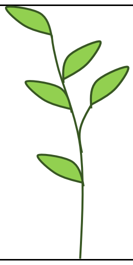	Reach light by increasing plant height
WM	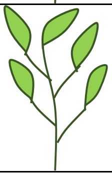	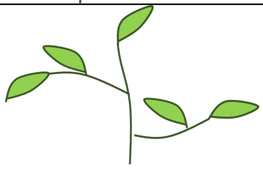	Avoid shade cast by neighbour by growing laterally
RLH	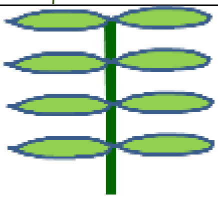	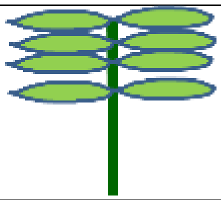	Reach light by moving leaf area toward the top
	**Negative response parameter mu**		
	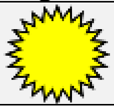	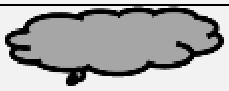	Reason for response to shading
SLA	No negative values in our experiments		
LBR	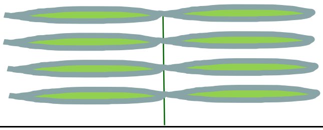	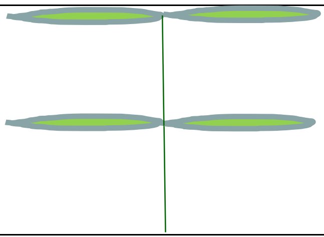	Reach light by increasing stem length
HM	Rare		
WM	Rare		
RLH	Rare		

B.2 How to estimate plasticity/morphology parameters in garden plots

B.2.1 Objective

The present section describes how to organize and analyze the data measured in garden plots for estimating plasticity/morphology parameters. This section was taken from the supplementary material of Colbach et al. (2020) and completed with data from the present experiments.

B.2.2 Measured data

B.2.2.1 Biomass and total leaf area

The measured data are collected in data files (e.g. excel), with one line per plant and the following columns:

- Sampling date,
- Species and variety,
- Shading treatment (sunny vs. shaded),
- Plant stage at sampling,
- Block number in experiment,
- Plant number,
- Plant height (cm),
- Plant width (cm),
- Area (cm^2) of:
  - Leaves (including petioles),
  - Stems,
  - Reproductive parts (flowers, seeds),
  - Total,
- Dry biomass (g) of
  - Leaves,
  - Stems,
  - Reproductive parts (flowers, seeds),
  - Total,

B.2.2.2 Growth stages

Plant growth stages must be monitored to determine when to sample (e.g. onset of flowering). Samples are usually taken at the following stages:

- 2 leaves,
- 4 leaves,
- 8 leaves for dicots, tillering for monocots,
- Onset of flowering,
- End of flowering.

B.2.2.3 Relative leaf area vs. relative plant height

The following data must be collected

- **Date of sampling**
- **Species and variety**
- **Treatment: sun vs. shade**
- **Plant stage at sampling**
- **Block number in experiment**
- **Plant number**
- Leaf area (cm^2) in a layer z (LA_z)
- Layer z, ranging from 1 (closest to soil surface) to 10 (top of plant)

These data result from image analysis of pictures showing vertical plant profiles, estimating leaf area in successive layers (usually 10).
Variables in **blue** are the same as in section B.2.2.1.

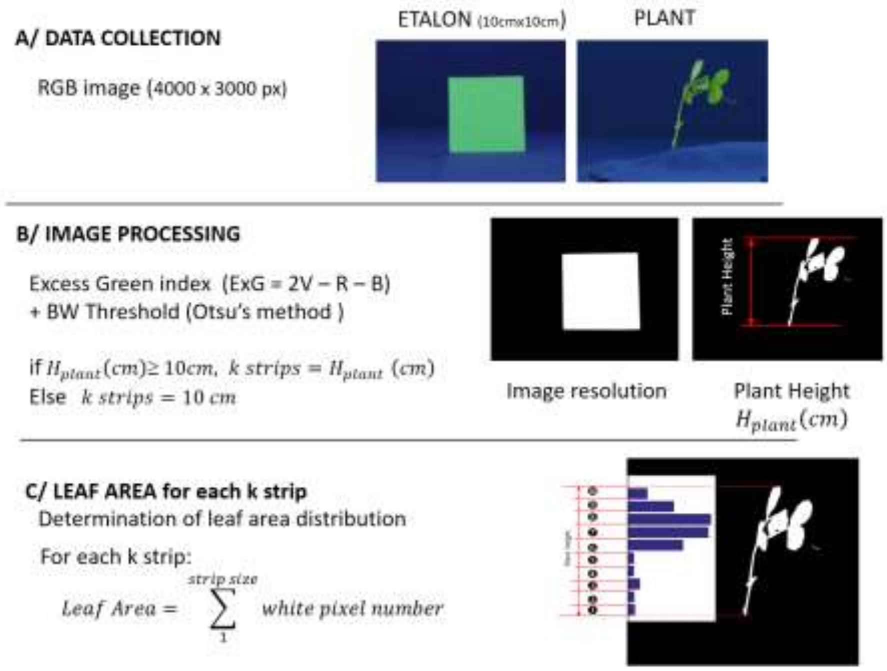

Figure 13 Flowchart of the image processing: Flowchart of the image processing: data acquisition (A), image processing (B), and output data: leaf area determined for each strip depending of the plant height (C) (Christelle Gée)

The methodological framework is presented in Fig.00 with the three main steps: data acquisition (**A**), image processing (**B**), and output data: leaf area determined for each strip depending of the plant height (**C**)

B.2.2.4 Temperature and incident light

Temperature and incident light must be measured continuously from plant emergence to sampling date and collected in a separate file.

B.2.2.5 Shading

During the experiments, incident photosynthetically active radiation (PAR) was measured with six quantum sensors (PAR; silicium sensors; Solems, Palaiseau, France) placed at 60 cm, 90 cm and 110 cm above soil surface, inside and outside the shading cage. Measurements were taken every 600 s and stored in a data logger (DL2e; Delta-T Devices, Cambridge, UK).The electric signal in mV (millivolts) was translated into PAR in µmol/m²/s by multiplying the measured value by the sensor's calibration coefficient. The average daily shading was estimated as 1 - the slope of the regression of the PAR measured at a given height inside vs outside the shading cage. Daily shading and the shading index since emergence (section B.1.2) inside the cage are identical as daily shading is constant over time.

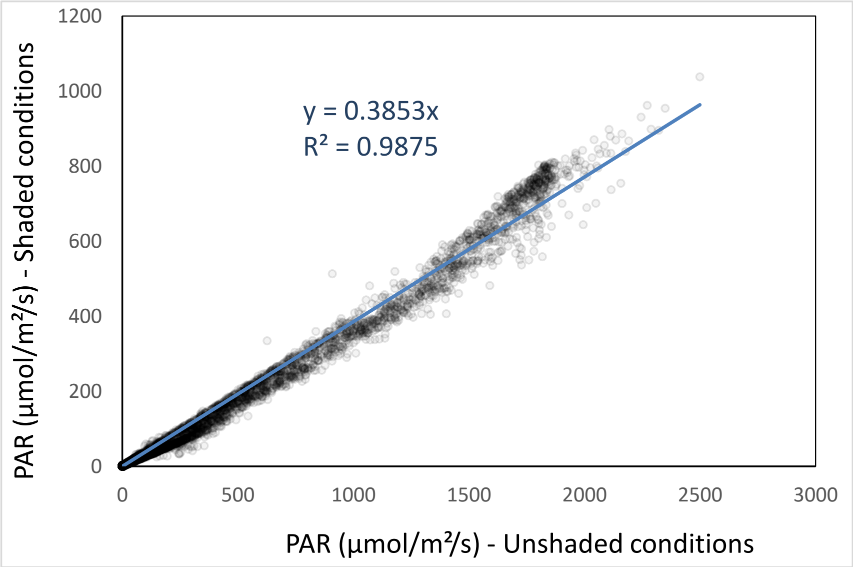

Figure 14. Incident photosynthetically active radiation (PAR) at a given distance from soil surface inside vs. outside the shading cage. Example of measurements from March to June 2017. Daily shading and shading index since plant emergence are 1-0.3853 = 0.6147 (Nathalie Colbach CC BY-NC-SA)

B.2.3 Calculate parameter values

B.2.3.1 List of parameters

The parameters of Table 6.B and C (section B.1.1) are to be calculated for each variety and sampling date

B.2.3.2 Potential morphology

B.2.3.2.1 Default situation

The values for several parameters/variables are simply calculated for each variety and sampling date as the mean of the four (or more) plants sampled at this stage in sunny conditions. This applies to the following parameters:

Parameter name	Relative advance of growth stage at the time of parameter measurement	Unit	Range
Potential morphology (morphology variables in unshaded conditions)			
SLA	Specific Leaf Area (leaf area vs leaf biomass)	$cm^2 \cdot g^{-1}$	[0;∞]
LBR	Leaf biomass ratio (leaf biomass vs total above-ground biomass)	none	[0;1]
H	Plant height	cm	[0;∞]
W	Plant width	cm	[0;∞]

H and W are used to calculate maximum plant height and width, which are considered to be easily measured species traits that will be used in the functional relationships. This is though only possible if there were no missing sampling dates for the variety.

B.2.3.2.2 Particular case of b_HM and b_WM

This applies to the following parameters:

Parameter name	Relative advance of growth stage at the time of parameter measurement	Unit	Range
Potential morphology (morphology variables in unshaded conditions)			
b_HM	Shape parameter b for specific plant height	none	]0;∞]
b_WM	shape parameter b for specific plant width	none	]0;∞]

These two parameters are estimated for each sampling date by fitting the following equation to height or width data measured on all the plants (eight or more) in both unshaded and shaded conditions at a given sampling date:

$$\log_n(\text{height}) = a + b \log_n(\text{biomass}) + c\,SI$$

$\log_n$ (width) = a + b $\log_n$ (biomass) + c SI

SI = shading index (i.e. incident PARa inside the shaded area vs incident PARa in the unshaded area of the garden plots), usually 0.60 in our experiments (caution: do not use 60 for 60% for instance!). If b >0, these values are used for b_HM and b_WM. Otherwise it will be estimated from previous or following stages (see section B.2.4).

The values of c are used for HM_mu and WM_mu (see section B.2.3.3.3). This applies to the following parameters:

Parameter name	Relative advance of growth stage at the time of parameter measurement	Unit	Range
Response to shading (variation in morphology variables with shading intensity)			
HM_mu	Response of specific height to shading	none	[-∞;∞]
WM_mu	Response of specific width to shading	none	[-∞;∞]

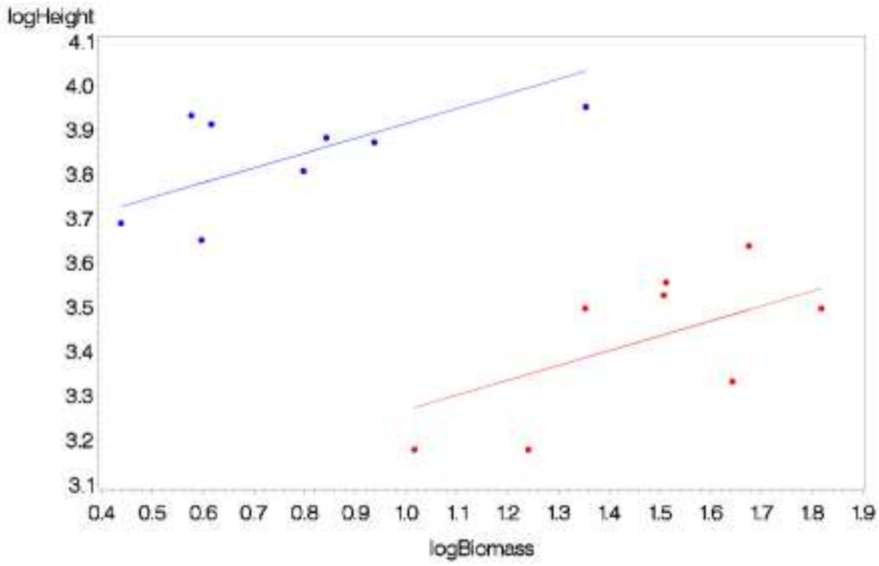

Figure 15. Example of fitting plant height vs above-ground plant biomass for two shading conditions (red: no shading, blue: 0.61 shading index). Variety Isard at flowering onset date (Nathalie Colbach [CC BY-NC-SA]).

B.2.3.2.3 Particular case of HM and WM

This applies to the following parameters:

Parameter name	Relative advance of growth stage at the time of parameter measurement	Unit	Range
Potential morphology (morphology variables in unshaded conditions)			
HM0	Specific (allometric) plant height (height vs. total above-ground biomass ratio)	$cm \cdot g^{-1}$	[0;∞]
WM0	Specific (allometric) plant width (width vs. total above-ground biomass ratio)	$cm \cdot g^{-1}$	[0;∞]

Once b_HM and b_WM are calculated for each species or variety and sampling date (section B.2.3.2.2)

- Calculate specific plant height and width for each plant of each light condition (including shaded conditions) and each sampling date as follows:
  - HM = height/biomass^{b_HM}
  - WM = height/biomass $^{b\_WM}$
- For each stage, HM0 and WM0 are respectively HM and WM averaged over all plants from the unshaded conditions.

B.2.3.2.4 Particular case of RLH0 and b_RLH

This applies to the following parameters:

Parameter name	Relative advance of growth stage at the time of parameter measurement	Unit	Range
Potential morphology (morphology variables in unshaded conditions)			
RLH0	Median relative leaf height (relative plant height below which 50% of leaf area are located)	cm cm^{-1}	[0;1]
b_RLH	Shape parameter for leaf distribution along plant height	none	]0;∞]

For each plant of each light condition and each sampling date,

- Calculate the relative cumulated leaf area (cm^2 cm^{-2}) in each measurement layer z as $RCLA_z = \sum_{i=1}^{z} LA_i/LA$ where LA_i is the leaf area in layer i and LA is the total leaf area of the plant
- Calculate the relative height for each layer z as $rh_z = z/NZ$ with NZ_i the number of layers used for measuring leaf area during picture analysis (section B.2.2.3)
- Fit relative cumulated leaf area $RCLA_z$ vs. relative height rh_z

$$RCLA_z = \frac{1-RLH^b}{1-2\cdot RLH^b}\cdot\left(1-\frac{1}{1+\left(\frac{1}{RLH^b}-2\right)\cdot rh_z^b}\right)$$

  where RLH (cm·cm^{-1}) is the relative height corresponding to RCLA=0.5, and b (adimensional) is proportional to the slope at RLH.
- For each stage, calculate RLH0 and b_RLH as the average of respectively RLH and b of all plants from unshaded conditions.

B.2.3.3 Response to shading

B.2.3.3.1 Principle explained with specific leaf area SLA

For each sampling date and species/variety, fit the following regression to SLA measured in unshaded and shaded conditions (eight plants or more):

[3] $\log_n(SLA) = a + b\ SI$

SI = shading index, usually 0.60 in our experiments (caution: do not use 60 for 60% for instance!). SLA_mu is b.

In the present experiment, there were only two light levels, unshaded (SI = 0) and shaded (SI = 0.6 for instance). But the adequacy of the equation was demonstrated by Munier-Jolain et al (2014) who tested five different light levels.

B.2.3.3.2 List of parameters to which the principle applies

This applies to the following parameters:

Parameter name	Relative advance of growth stage at the time of parameter measurement	Unit	Range
Response to shading (variation in morphology variables with shading intensity)			
SLA_mu	Response of specific leaf area to shading	none	[-∞;∞]
LBR_mu	Response of leaf biomass ratio to shading	none	[-∞;∞]
HM_mu	Response of specific height to shading	none	[-∞;∞]
WM_mu	Response of specific width to shading	none	[-∞;∞]
RLH_mu	Response of median relative leaf height to shading	none	[-∞;∞]

B.2.3.3.3 Particular case of HM_mu and WM_mu

HM_mu and WM_mu are estimated in the same model as b_HM_var0 and b_WM_var0 (section B.2.3.2.2).

B.2.4 "Smooth" parameter values

B.2.4.1 Interpolate missing data

B.2.4.1.1 Particular case of cotyledon stage

Shade response parameters are put to zero as plants are assumed to have no plasticity at emergence. If no measurements are available at plant emergence, values from the next stage are used for potential, unshaded parameters, and shading parameters are put to zero.

B.2.4.1.2 Particular case of mature plants

Specific leaf area SLA is often nil for this stage as there are no more leaves. Often, there are no measurements. In that case

- Leaf biomass ratio LBR is put to zero (no more green leaves for photosynthesis)
- Values of previous stage are used for other potential unshaded parameters,
- Shading response parameters LBR_mu and SLA_mu = 0 (no more plasticity for leaves as there no more leaves)

B.2.4.1.3 Other stages

If any parameters are missing for intermediate stages (e.g. b_HM and b_WM when b < 0, section B.2.3.2.2), the average of the values of the previous and subsequent stages is used.

B.2.4.2 Fit regression with plant age

The objective is to "smooth" parameter values vs. plant age, regardless of whether values are missing for some stages. Then, values predicted with these regressions will be used for all parameters and stages, again regardless of whether there were missing values. This will correct for inter-stage variability due to the low number of sampled plants. It will also give us values for the same stages for all species and varieties, regardless of the actual measurement dates in the experiments.

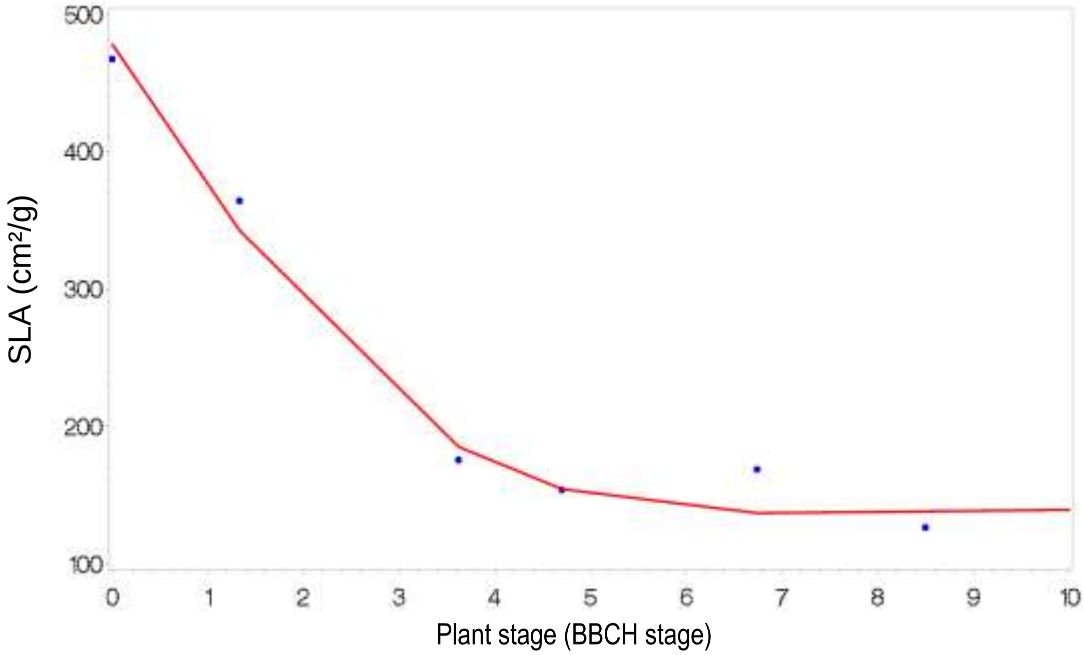

Figure 16. Fitting a local non-parametric regression of a morphology/plasticity parameters with plant age (line) to measurements (dots). Example of potential specific leaf area SLA0 of *Pisum sativum* cv China (Nathalie Colbach CC BY NC ND)

B.2.4.3 Data

B.2.4.3.1 General

The data used for smoothing are the parameters of section B.2.3.1 for as many stages as possible.

B.2.4.3.2 Additional data points based on assumptions

The following data are added:

- Response to shading at emergence (BBCH stage = 0) is assumed to be nil. Thus, for each species, data lines with shade response parameters mu=0 are added
- If no measurements were available at emergence (BBCH stage = 0), monocots are assumed to consist of leaves only (i.e. LBR0 = 1). Thus, for each monocot species, data lines with LBR0 = 1 are added

B.2.4.3.3 Particular case of HM and WM

HM and WM values can be missing at stages even if plant height and width were measured. This occurs when b values of the linear regression linking height or width to biomass were negative (see section B.2.3.2.2). In that case:

- estimate the missing b_HM and b_WM via smoothing (section B.2.4.5),
- calculate HM and WM for each plant of the concerned stages,
- calculate HM0 and WM0 as the averages of respectively HM and WM in unshaded conditions (section B.2.3.2.1).

B.2.4.4 Plant age

Plant stages are transformed into a continuous variable, based on the BBCH scale. Parameter values will be predicted for the following plant ages: 0 (emergence), 1, 2, 3, 4, 5, 6, 7, 8, 9, 10 (death).

B.2.4.5 LOESS regression

Local non-parametric regressions with total smooth are fitted to each parameter vs. time. Local regression to obtain a predicted value at a given point in the predictor space is done by doing a least squares fit that uses all data points in a local neighbourhood of the given point. This method has the advantage of not assuming any general shape of the relationship between parameter and time.

Linear smoothing is used if less than 6 sampling dates (7 for b parameters), quadratic local polynomial otherwise. Values are then predicted for sampling dates and for time $\in \{0, 1, 2, 3, 4, 5, 6, 7, 8, 9, 10\}$. Predicted values can be capped, e.g. predicted SLA0 must be > 0, predicted LBR0 must be ≥ 0 and ≤ 1 etc. Often, predicted values are capped by minimum and maximum observed values to avoid extremely small or large values in case of extrapolation for late stages when only a few early stages were measured.

B.2.5 Summary

	Additional data	Minimum number of sampling dates for quadratic smoothing	Minimum predicted value	Maximum predicted value	Various
Potential (unshaded) parameters					
SLA0		5	≥ 0		
LBR0	LBR_var0 = 1 at stage = 0 if monocot	5	≥ 0	≤1	
b_HM		5	≥ smallest measured value	≤ largest measured value	
HM0	Estimate missing HM0 values with predicted b_HM (section B.2.4.3.3)	5	≥ smallest measured value	≤ largest measured value	
b_WM		5	≥ smallest measured value	≤ largest measured value	Also capped by smallest and largest observed values when only 2 measurement dates or less
WM0	Estimate missing WM0 values with predicted b_WM (section B.2.4.3.3)	5	≥ smallest measured value		
RLH0		5	≥ 0	≤1	Also capped by smallest and largest observed values in case of linear smoothing
b_RLH		5	≥ smallest measured value	≤ largest measured value	
Response to shading					
SLA_mu	Mu=0 if stage = 0	5	≥ smallest measured value	≤ largest measured value	
LBR_mu	Mu=0 if stage = 0	5	≥ smallest measured value	≤ largest measured value	
HM_mu	Mu=0 if stage = 0	5	≥ smallest measured value	≤ largest measured value	
WM_mu	Mu=0 if stage = 0	5	≥ smallest measured value	≤ largest measured value	
RLH_mu	Mu=0 if stage = 0	5	≥ smallest measured value	≤ largest measured value	

C.Additional results from the garden plot experiments

C.1 Parameter values of plant morphology and shading response

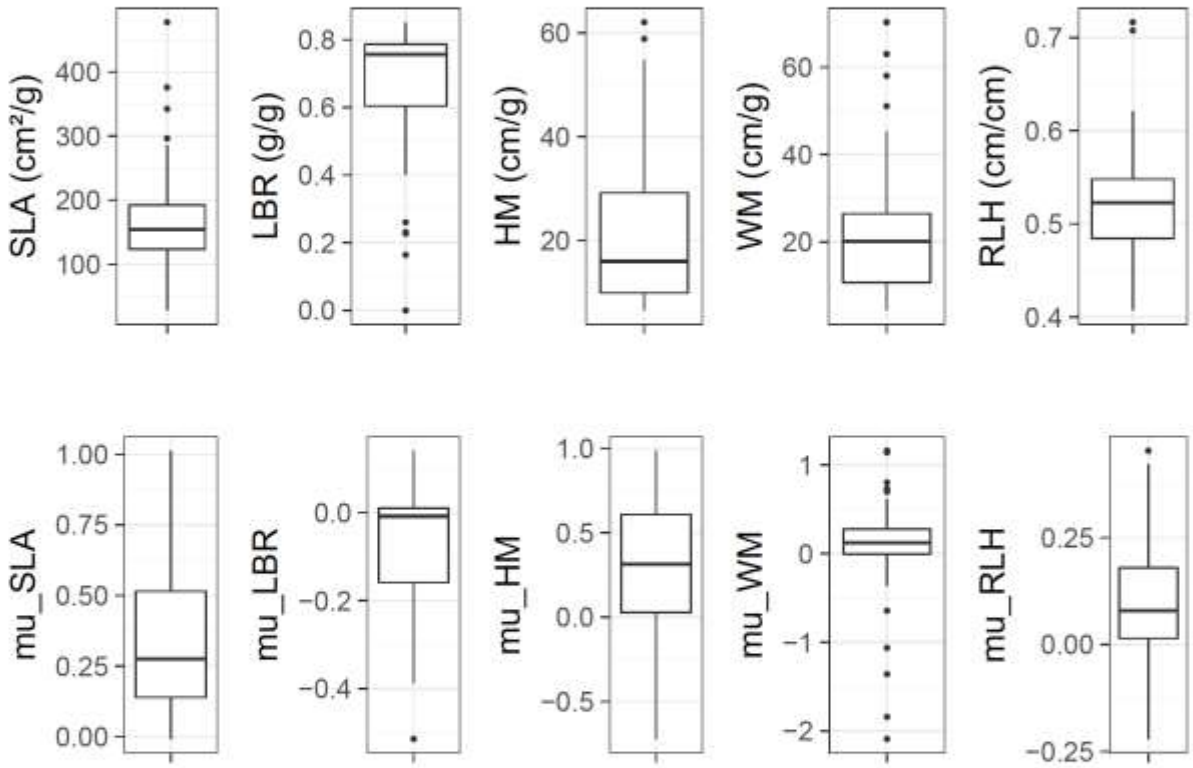

Figure 17. Boxplots of xxxx (Nathalie Colbach CC BY NC SA)

Table 8xxxx

	Min.	1st Qu.	Median	Mean	3rd Qu.	Max.
SLA	27.93	123.58	154.41	167.03	192.18	477.45
LBR	0.00	0.60	0.76	0.68	0.79	0.85
HM	6.51	9.90	15.97	21.59	29.16	62.05
WM	4.31	10.71	20.16	22.00	26.45	70.26
RLH	0.41	0.48	0.52	0.52	0.55	0.72
b_HM	0.04	0.12	0.21	0.21	0.27	0.41
b_WM	0.17	0.30	0.39	0.42	0.55	0.81
b_RLH	1.54	2.12	2.44	2.51	2.73	6.83
mu_SLA	-0.01	0.14	0.28	0.34	0.51	1.01
mu_LBR	-0.51	-0.16	-0.01	-0.07	0.01	0.14
mu_HM	-0.72	0.03	0.31	0.32	0.61	0.99
mu_WM	-2.09	0.00	0.12	0.09	0.27	1.16
mu_RLH	-0.22	0.01	0.08	0.10	0.18	0.45

C.2 Correlations among morphology and shading-response parameters

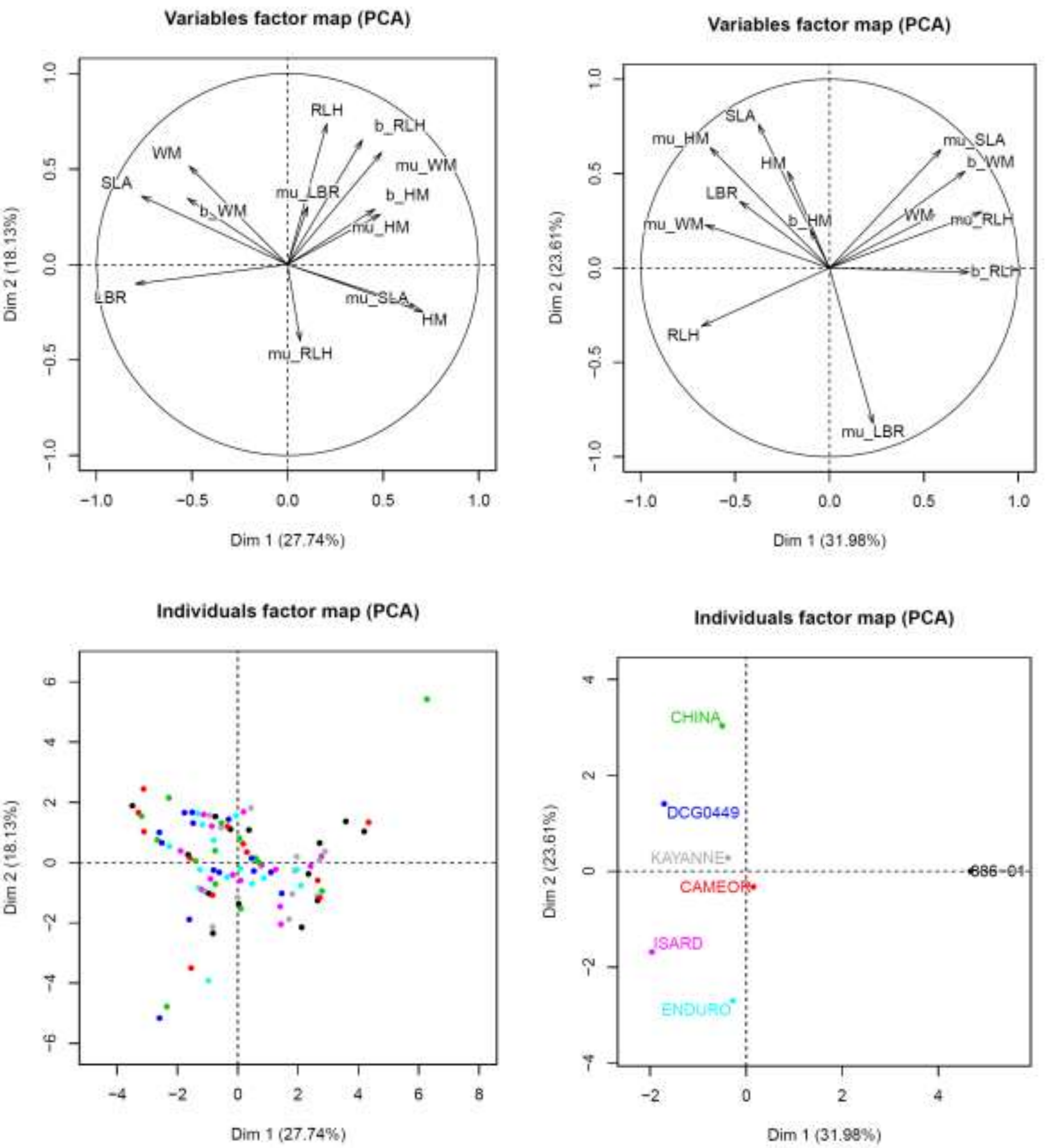

Figure 18. Principal Component Analysis on parameter values per plant (7 varieties x 11 stages) (left) and per variety (with values averaged over 11 stages) (right) (Nathalie Colbach).

Table 9. Correlations among parameters values of plant morphology and shading response. Cells are coloured from red (-1) to green (1). Bold cells show Pearson correlation coefficients that are significantly different from zero at p=0.05. For explanations on parameters, see section B.1.1

A. Per plant (7 variety x 11 stages)

Parameter		SLA	LBR	HM	b_HM	WM	b_WM	RLH	b_RLH	mu_SLA	mu_LBR	mu_HM	mu_WM	mu_RLH
Specific Leaf Area	SLA	1.00	**0.59**	**-0.42**	**-0.31**	**0.49**	**0.46**	0.15	-0.12	**-0.66**	0.03	**-0.24**	-0.12	-0.14
Leaf biomass ratio	LBR	**0.59**	1.00	**-0.61**	-0.17	**0.22**	**0.27**	**-0.29**	**-0.42**	**-0.71**	-0.12	**-0.29**	**-0.40**	0.05
Specific plant height	HM	**-0.42**	**-0.61**	1.00	0.02	**-0.25**	**-0.25**	-0.09	0.08	**0.61**	-0.05	**0.40**	0.14	0.00
Shape parameter b for HM	b_HM	**-0.31**	-0.17	0.02	1.00	**-0.21**	-0.15	0.18	**0.22**	0.16	0.11	**0.35**	**0.45**	-0.09
Specific plant width	WM	**0.49**	**0.22**	**-0.25**	**-0.21**	1.00	**0.73**	0.06	0.11	**-0.35**	-0.08	0.10	-0.01	**-0.30**
Shape parameter b for WM	b_WM	**0.46**	**0.27**	**-0.25**	-0.15	**0.73**	1.00	-0.09	0.16	-0.14	-0.02	-0.20	-0.11	0.03
Median relative leaf height	RLH	0.15	**-0.29**	-0.09	0.18	0.06	-0.09	1.00	**0.54**	-0.18	**0.28**	0.15	**0.50**	**-0.26**
Shape parameter for RLH	b_RLH	-0.12	**-0.42**	0.08	**0.22**	0.11	0.16	**0.54**	1.00	**0.32**	**0.45**	0.08	**0.39**	-0.10
SLA response to shading	mu_SLA	**-0.66**	**-0.71**	**0.61**	0.16	**-0.35**	-0.14	-0.18	**0.32**	1.00	-0.03	**0.24**	0.14	**0.29**
LBR response to shading	mu_LBR	0.03	-0.12	-0.05	0.11	-0.08	-0.02	**0.28**	**0.45**	-0.03	1.00	**-0.22**	0.11	0.19
HM response to shading	mu_HM	**-0.24**	**-0.29**	**0.40**	**0.35**	0.10	-0.20	0.15	0.08	**0.24**	**-0.22**	1.00	**0.56**	-0.16
WM response to shading	mu_WM	-0.12	**-0.40**	0.14	**0.45**	-0.01	-0.11	**0.50**	**0.39**	0.14	0.11	**0.56**	1.00	-0.10
RLH response to shading	mu_RLH	-0.14	0.05	0.00	-0.09	**-0.30**	0.03	**-0.26**	-0.10	**0.29**	0.19	-0.16	-0.10	1.00

B. Per variety (with values averaged over 11 stages)

Parameter		SLA	LBR	HM	b_HM	WM	b_WM	RLH	b_RLH	mu_SLA	mu_LBR	mu_HM	mu_WM	mu_RLH
Specific Leaf Area	SLA	1.00	0.21	0.55	-0.17	0.03	0.11	0.18	-0.41	0.18	**-0.93**	0.55	0.16	-0.11
Leaf biomass ratio	LBR	0.21	1.00	-0.24	0.51	-0.49	-0.13	0.07	-0.17	0.27	-0.07	0.65	**0.85**	-0.07
Specific plant height	HM	0.55	-0.24	1.00	-0.02	0.23	-0.09	0.05	-0.12	-0.01	-0.59	0.56	-0.20	-0.23
Shape parameter b for HM	b_HM	-0.17	0.51	-0.02	1.00	0.28	0.20	0.21	0.43	0.13	0.18	0.50	0.35	-0.24
Specific plant width	WM	0.03	-0.49	0.23	0.28	1.00	**0.79**	-0.11	0.48	0.18	-0.24	-0.19	-0.49	0.18
Shape parameter b for WM	b_WM	0.11	-0.13	-0.09	0.20	**0.79**	1.00	-0.51	0.48	0.63	-0.31	-0.20	-0.24	0.64
Median relative leaf height	RLH	0.18	0.07	0.05	0.21	-0.11	-0.51	1.00	-0.29	-0.72	0.01	0.20	0.12	**-0.92**
Shape parameter for RLH	b_RLH	-0.41	-0.17	-0.12	0.43	0.48	0.48	-0.29	1.00	0.55	0.43	-0.25	-0.56	0.44
SLA response to shading	mu_SLA	0.18	0.27	-0.01	0.13	0.18	0.63	-0.72	0.55	1.00	-0.20	0.11	-0.10	**0.83**
LBR response to shading	mu_LBR	**-0.93**	-0.07	-0.59	0.18	-0.24	-0.31	0.01	0.43	-0.20	1.00	-0.48	-0.15	-0.01
HM response to shading	mu_HM	0.55	0.65	0.56	0.50	-0.19	-0.20	0.20	-0.25	0.11	-0.48	1.00	0.60	-0.36
WM response to shading	mu_WM	0.16	**0.85**	-0.20	0.35	-0.49	-0.24	0.12	-0.56	-0.10	-0.15	0.60	1.00	-0.26
RLH response to shading	mu_RLH	-0.11	-0.07	-0.23	-0.24	0.18	0.64	**-0.92**	0.44	**0.83**	-0.01	-0.36	-0.26	1.00

C.3 Correlations among all variety parameters

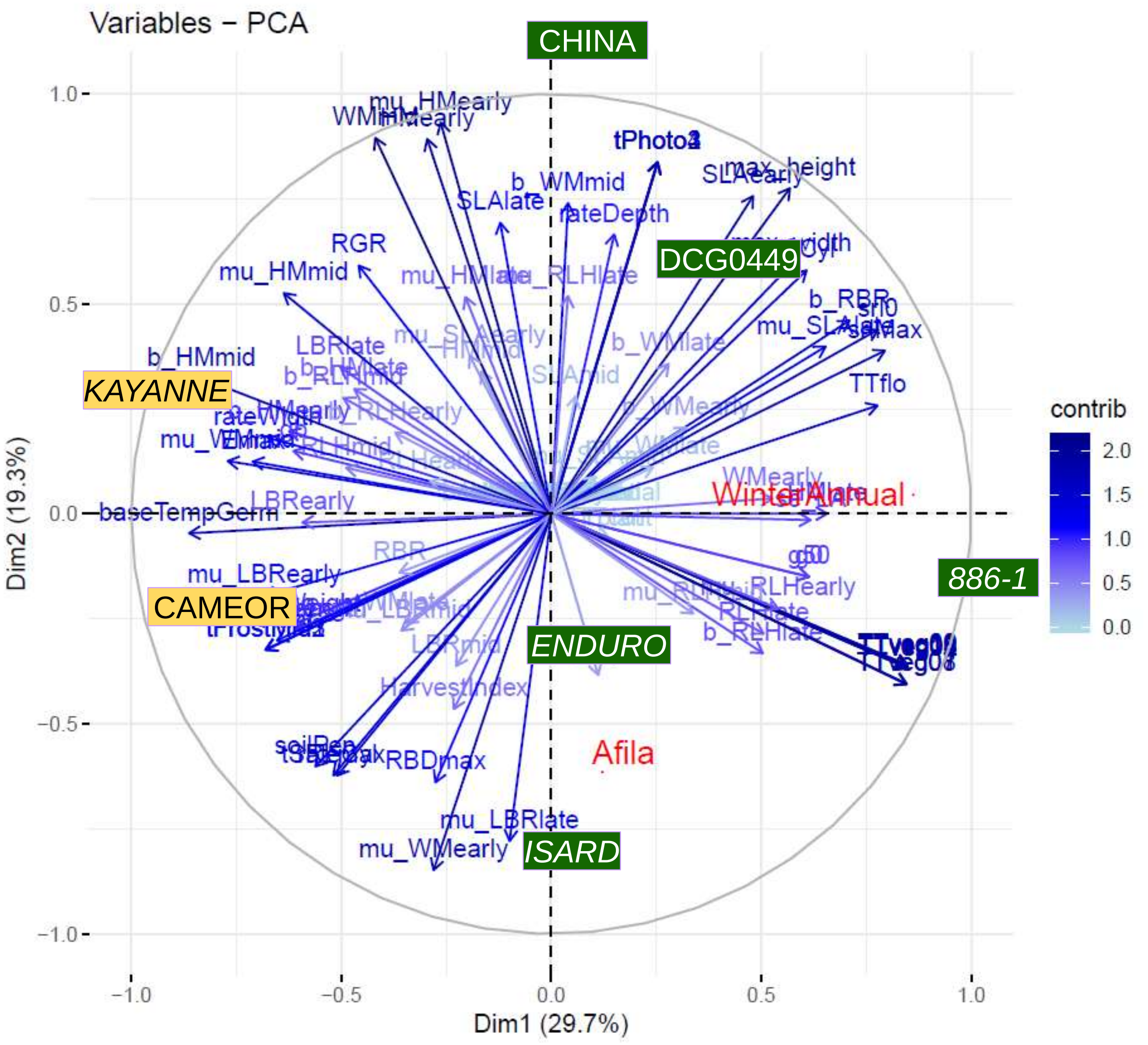

Figure 19. Principal Component Analysis on the 220 FLORSYS parameters and 7 pea varieties. To simplify, the 11 values corresponding to the 11 stages for the morphology and shading-response parameters were aggregated into early (0-2), vegetative (3-7) and late (8-10) parameters. Spring varieties are highlighted in yellow. The "afila" and "WinterAnnual" characteristics were projected onto the PCA. The darker the colour was, the more the parameter contributed to the first two axes. The longer the arrow was, the better the parameter was represented (Nathalie Colbach).

D. Additional results from the virtual experiments (simulations)

D.1 Preliminary simulation study aiming to link weed species to weed impact

A preliminary simulation study was run to analyse the impact of weed-flora composition on crop production and weed (dis)services.

D.1.1 Material and methods

The simulation study was based on 900 randomly built cropping systems, using a Latin Hypercube Sampling (LHS) plan and the following rules:

- Crop were chosen from oilseed rape, winter wheat, pea and barley, with a least one pea and one wheat crop in the rotation,
- Pea varieties were chosen from the same 18 varieties as in the main simulation study (7 actual, 10 virtual, 1 based on STICS simulations,
- Cropping techniques were drawn randomly, respecting agronomic constraints for each crop.

Simulations were run, using a soil and weather recorded in Burgundy. Each cropping system was simulated over 12 years and repeated with 5 different weather series. The whole simulation series was repeated after removing all herbicides from the cropping systems.

The analysed outputs were the crop yield and the weed (dis)services described in section 0. The link between weed-species traits and weed-impact indicators was analysed, using RLQ and 4th-corner analyses. using the library ade4 (Chessel *et al.*, 2004) of R (R Core Team, 2016). The RLQ analysis was initially developed to investigate correlations between cultural techniques (R matrix) and species traits (Q matrix) via weed species densities (L matrix). Here, we used the simulated weed-impact indicators for the R matrix. The Q matrix consisted of FLORSYS parameters for the 26 weed species. The L matrix comprised the simulated weed plant density. Weed species wereaggregated into functional groups based on a Ward ascendant hierarchy classification using the hclust() function of R according to the Euclidian distances separating coordinates of species in the RLQ multidimensional space.

D.1.2 Create contrasting weed species pool for further simulations

Based on the RLQ analysis (Figure 20), two particular contrasting weed species pools were identified:

- The "harmful" pool consisted of the six species (groups B and D in the lower right-hand quadrant of Figure 20) that increased yield loss without increasing trophic resources for bees,
- The "harmful dicots promoting bee food" (groups E and F in the lower left-hand quadrant of Figure 20) that both increased yield loss and trophic resources for bees.

The third weed-flora pool used in the manuscript consisted of all 26 species.

A. Principal Component Aanalysis on indicators of crop production and weed (dis)services

B. Correspondence Analysis on the weed species densities

C. Principal Component Analysis on weed-species traits

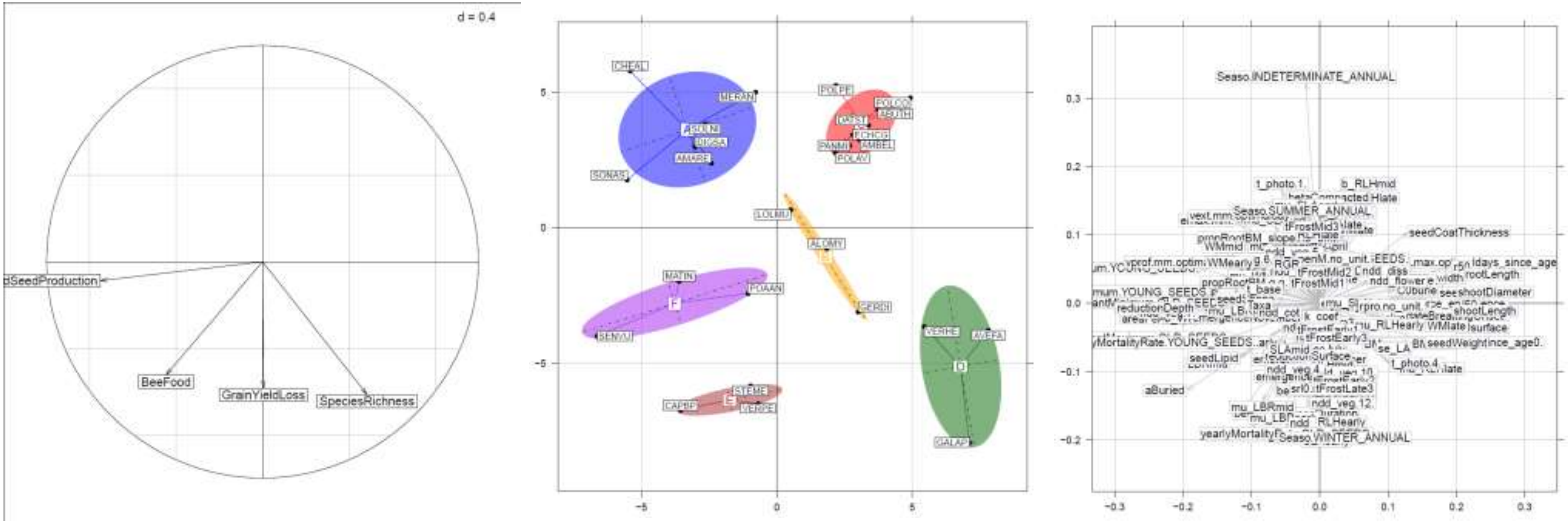

Figure 20. The weed species (shown with EPPO codes) and species traits that drive weed impacts on crop production and weed (dis)services. Synthetic representation of the RLQ results with crop production and weed (dis)services simulated by FLORSYS as matrix R, simulated weed plant densities as matrix L, and FLORSYS species parameters as matrix Q. Weed species were clustered into groups, following a Ward ascendant hierarchy classification. (Nathalie Colbach 2021 CC BY-NC-SA)

D.2 Further details on the simulation plan

D.2.1 Correlations among CART inputs

To check that the simulation succeeded in decorrelating cropping system practices, Pearson correlation coefficients were calculated among CART inputs, using the cor() function of R.

Table 10. Correlations among inputs to Classification And Regression Trees (CART). Median values of Pearson correlation coefficients between pea parameters, crop management techniques, rotation and situation

Variety type	Variable type	Variable type								
		Trait	Pea	Wheat	Sunflower	OSR	Barley	Maize	Rotation	Situation
Spring	Parameter	0.30	0.03	0.03	0.05	0.02	0.04	0.06	0.02	0.02
	Management techniques in									
	Pea	0.03	0.06	0.05	0.07	0.05	0.05	0.07	0.08	0.07
	Wheat	0.03	0.05	0.06	0.07	0.05	0.05	0.07	0.08	0.07
	Sunflower	0.05	0.07	0.07	0.08	0.07	0.08	0.07	0.07	0.07
	OSR	0.02	0.05	0.05	0.07	0.06	0.05	0.07	0.08	0.08
	Barley	0.04	0.05	0.05	0.08	0.05	0.08	0.07	0.06	0.09
	Maize	0.06	0.07	0.07	0.07	0.07	0.07	0.09	0.08	0.07
	Rotation	0.02	0.08	0.08	0.07	0.08	0.06	0.08	0.21	0.12
	Situation	0.02	0.07	0.07	0.07	0.08	0.09	0.07	0.12	0.17
Winter	Parameter	0.23	0.02	0.02	0.05	0.02	0.04	0.05	0.02	0.02
	Management techniques in									
	Pea	0.02	0.06	0.04	0.05	0.04	0.04	0.06	0.08	0.06
	Wheat	0.02	0.04	0.05	0.05	0.04	0.04	0.06	0.07	0.07
	Sunflower	0.05	0.05	0.05	0.07	0.06	0.06	0.06	0.06	0.07
	OSR	0.02	0.04	0.04	0.06	0.06	0.04	0.06	0.07	0.08
	Barley	0.04	0.04	0.04	0.06	0.04	0.06	0.06	0.06	0.07
	Maize	0.05	0.06	0.06	0.06	0.06	0.06	0.08	0.07	0.07
	Rotation	0.02	0.08	0.07	0.06	0.07	0.06	0.07	0.21	0.13
	Situation	0.02	0.06	0.07	0.07	0.08	0.07	0.07	0.13	0.17

D.2.2 The actual and virtual varieties

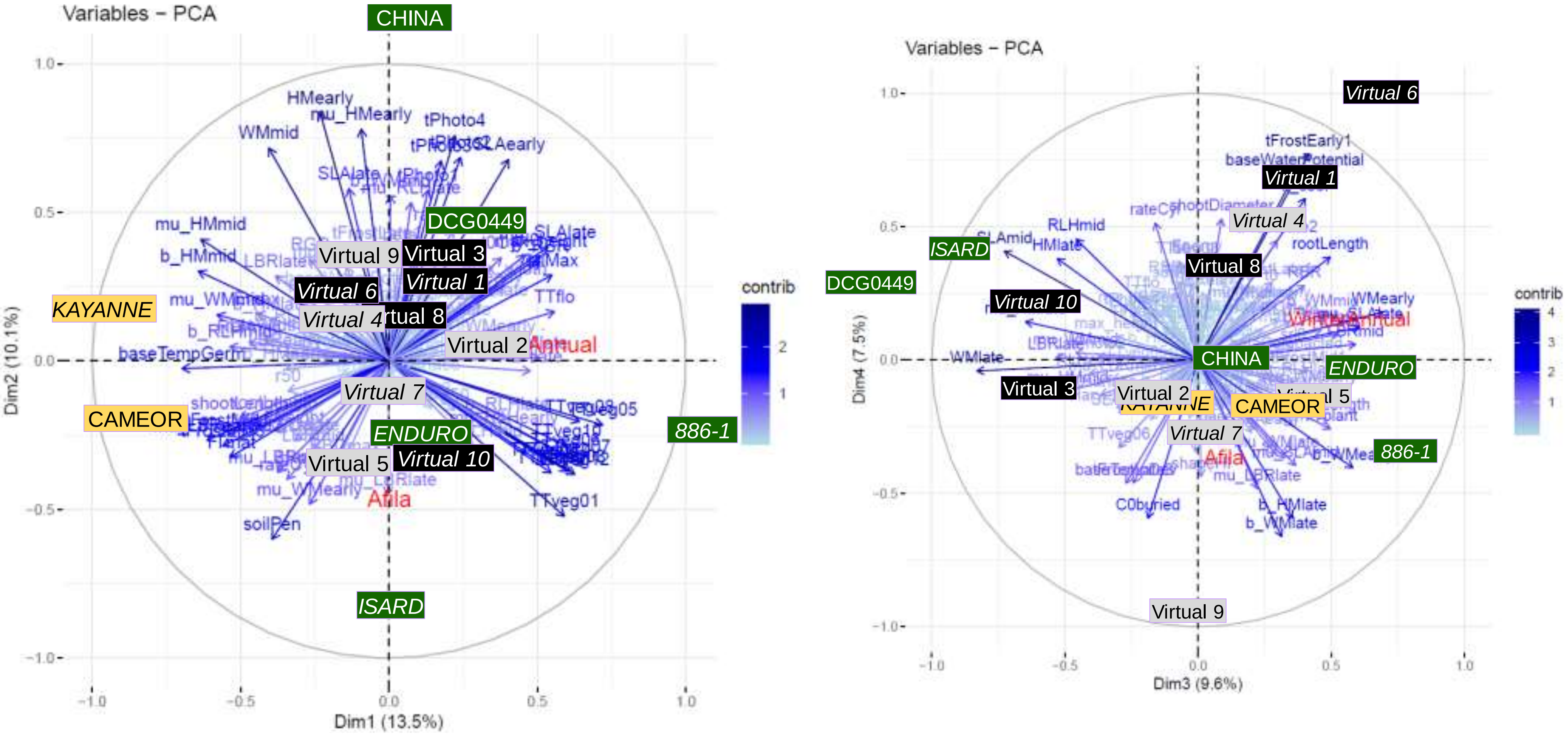

Figure 21. Principal Component Analysis (PCA) of the pea parameters that describe the pea varieties in FLORSYS. Spring varieties are in yellow (for actual varieties) and grey (for virtual varieties), winter varieties in green and black.(Nathalie Colbach [CC BY-NC-ND])

D.3 Further explanations on Classification and Regression Trees (CART)

D.3.1 Surrogate variables

Considering a node, let X be the selected predictor (called "primary variable" in the following). The node is split into two child nodes according to $X>x$ where x is the selected split value. Let n be the number of individuals in the parent node, and n_L (resp. n_R) the number of individuals in the left (resp. right) child node. Then $n=n_L+n_R$. A surrogate variable for the parent node is a predictor X' for which a split value x' can be found such that the resulting child nodes are similar to the original ones in that they contain almost the same individuals. Intuitively, surrogate variables explain the same component of variability as the primary variable, but they do not explicitly appear in the tree. As it would be erroneous to state that all variables that do not explicitly appear in the tree are of no importance in predicting the response variable, the variable importance (VIP) quantifies the amount of variability explained by each predictor in the tree, including the variability explained by nodes where the predictor is a surrogate. Then, a predictor may have a non-null importance even if never appears in the tree, because it acts as a surrogate in at least one node. But it also follows that the sum of the amount of variability explained by all non-null importance variables exceeds the total amount of variability explained by the tree, since a given variability component may be explained by a primary variables and surrogates.

D.3.2 Variable importance (VIP)

D.3.2.1 Amount of variability in a node

$$VarNode = \sum_{i=1}^{n} (y_i - \bar{y})^2$$

where n is the number of individuals that belong to the node, y_i the response variable value for the individual i, and $\bar{y}$ the mean of the response variable over the n individuals of the node.

D.3.2.2 Ratio and amount of variability explained by a node

Variability ratio explained by a node:

$$improve = 1 - \frac{VarNode(leftChildNode) + VarNode(rightChildNode)}{VarNode(parentNode)}$$

(this ratio is called "improve" in the CART literature).

Amount of variability explained by a node:

$$\mathrm{VarExpNode} = \mathrm{improve} \times VarNode(parentNode)$$

D.3.2.3 Raw and adjusted agreement of a surrogate

Raw agreement for a surrogate variable: considering the original left/right classification of individuals according to the primary variable, the raw agreement for a surrogate variable is the number of correctly classified individuals n_+ divided by n the total number of individuals in the node. A node is correctly classified the surrogate sends it in the same direction (left/right) than the primary variable.

"go with the majority rule": this rule emulates a naïve surrogate that would send all individuals to the direction where the primary variable sent the majority of individuals.

Let $n_{maj} = max(n_L, n_R)$, then the surrogate agreement is adjusted as follows to avoid selecting surrogates by chance only:

$$adjAgree = \frac{n_+ - n_{maj}}{n - n_{maj}}$$

D.3.2.4 Raw and relative variable importance (VIP) computation

The raw VIP of a predictor is the sum of the amounts of variability explained by each node for which the predictor is either the primary variable or a surrogate. In the latter case, the amount is weighted by its adjusted agreement. More formally:

```
rawVIP is initialized to 0 for each predictor
For each node
  Let X be the primary variable
  rawVIP(X) = rawVIP(X) + VarExpNode
  For each surrogate X'
    rawVIP(X') = rawVIP(X') + adjAgree*VarExpNode
  End for
End for
```

Being a sum of squared differences, the unity of the raw VIP is the squared unity of the response variable, which is not very intuitive. Moreover, being a sum over the tree nodes, the importance of a predictor can only grow with the size of the tree, making it difficult to compare the importance of a predictor between trees of different sizes. Thus we defined the relative VIP by dividing the raw VIP by the total amount of variability explained by the tree, which can be computed as the difference between the original variability (variability in the root node) and the amount of variability still lying in the set of leaf nodes.

$$\mathrm{totVarExpl} = \mathrm{VarNode(root)} - \sum_{\mathrm{i} \in leaves} \mathrm{VarNode(i)}$$

Note that this total amount of explained variability can equivalently be computed as the sum of the raw VIP computed as above by removing the inner loop on surrogate splits.

$$RelativeVIP(X) = \frac{rawVIP(X)}{totVarExpl}$$

D.3.2.5 Probability of positive relation between a predictor and the response

In order to quantify whether the predictor X varies in the same direction as the response variable, we compute a probability of positive relation as follows. For each node that involves X, the number of individuals is added to the numerator if X and the response variable move in the same direction, and to the denominator in any case.

```
Numerator(X) = 0
Denominator(X) = 0
For each node i for which X is either the primary variable or a surrogate,
containing ni individuals
      Denominator(X) = denominator(X) + ni
      If (X and Y both increase when moving to the left child node) OR
      (X and Y both decrease when moving to the left child node)
            Numerator(X) = numerator(X) + ni
      End if
End for
```

D.4 Variation in simulated output

Table 11. Distribution of indicator values of crop production and weed impacts simulated with FLORSYS

A. All cropping systems and years

	Indicator - unit											
	Potential crop yield	Actual crop yield	Grain yield loss	Potential pea yield	Actual pea yield	Pea yield loss	Species richness	Bee food offer	Field infestation	Herbicide use intensity	Weed seed production	
Statistics	MJ/ha	MJ/ha	% (t/t)	t/ha	t/ha	% (t/t)	Number of species	No unit	t/ha	TFI	seeds/m²	t/ha
**mean**	**123943**	**61704**	**47.7**	**3.6**	**1.7**	**56**	**15.8**	**0.36**	**2.25**	**1.96**	2.53E+05	2.1
min	0	0	-100.0	0.0	0.0	-99	0.0	0.00	0.00	0.00	0.00E+00	0.0
p05	31579	645	-1.9	0.4	0.0	0	5.0	0.00	0.03	0.00	1.10E+02	0.0
p10	49446	1974	0.6	0.8	0.0	3	5.0	0.00	0.13	0.00	7.88E+02	0.0
p25	67565	13052	10.0	2.1	0.2	22	9.0	0.06	0.73	1.00	1.46E+04	0.1
median	104664	47902	48.0	3.4	1.0	63	18.0	0.26	1.69	2.00	1.16E+05	1.0
p75	171446	82358	85.9	5.0	2.8	90	21.0	0.56	3.21	3.00	3.64E+05	2.9
p90	227701	152195	97.5	6.4	4.6	98	24.0	0.87	5.10	4.00	6.85E+05	6.1
p95	263460	196099	99.2	7.2	5.7	99	24.0	1.09	6.29	4.00	9.63E+05	8.2
max	482416	483420	100.0	11.5	11.3	100	26.0	3.24	19.81	4.66	3.99E+06	33.8

B. Years with actual pea varieties only

	Indicator - unit											
	Potential crop yield	Actual crop yield	Grain yield loss	Potential pea yield	Actual pea yield	Pea yield loss	Species richness	Bee food offer	Field infestation	Herbicide use intensity	Weed seed production	
Statistics	MJ/ha	MJ/ha	% (t/t)	t/ha	t/ha	% (t/t)	Number of species	No unit	t/ha	TFI	seeds/m²	t/ha
**mean**	**180672**	**82325**	**57.4**	**4.4**	**2.1**	**55**	**16.1**	**0.38**	**2.35**	**1.90**	3.08E+05	2.5
min	1	0	-99.1	0.0	0.0	-99	0.0	0.00	0.00	0.00	0.00E+00	0.0
p05	40802	758	0.4	1.2	0.0	0	5.0	0.00	0.04	0.00	1.46E+02	0.0
p10	67667	2005	2.6	1.9	0.1	2	6.0	0.00	0.18	0.00	1.92E+03	0.0
p25	127303	10225	23.1	3.2	0.3	20	10.0	0.07	0.80	1.00	3.09E+04	0.3
median	187378	50392	66.4	4.6	1.5	62	18.0	0.28	1.76	2.00	1.70E+05	1.4
p75	235823	141307	91.2	5.7	3.6	90	22.0	0.59	3.39	3.00	4.25E+05	3.6
p90	277161	213900	98.0	6.6	5.2	98	24.0	0.89	5.26	4.00	8.05E+05	6.8
p95	302188	248063	99.2	7.2	5.9	99	24.0	1.10	6.41	4.00	1.12E+06	8.6
max	426698	399332	100.0	9.2	9.1	100	26.0	2.72	17.26	4.66	3.99E+06	29.4

D.5 Variation in yield and weed (dis)services across situations

Each yield and weed-impact indicator was analysed with a linear model using the lm() function of R software version 4.0.1 (R Core Team, 2021) as a function of situation, year since simulation onset, their interaction as well as weather repetition. Average indicator values per situation were compared using the lsmeans() function and a Tukey test to account for the unbalanced data set as only years with pea were used in these analyses.

In the virtual experiments, the potential pea yield varied considerably among cropping systems of a given situation (e.g. conventional 3-year reference, organic, longer rotation…), but the means per situations were similar (Figure 22.A). There was though a slight increase in potential pea yield in the longest and most diverse rotation (6-year), as well as in the non-till systems.

Field infestation varied much more across situations (Figure 22.C). The strongest decrease compared to the reference occurred with the weed species pool consisting of weeds both harmful for crop production and beneficial for bees. Conversely, the highest field infestation occurred when starting with the most harmful weed species only. Any change in management reduced field infestation, particularly if more herbicides were sprayed (no till), but also if mechanical weeding was added (complete and organic) and/or tillage were intensified (organic), even when these changes occurred to the detriment of herbicide intensity (organic). Shorter rotations (2-year) increased field infestation and longer rotations decreased it (4-year and 6-year), though the latter effect was less visible in the 6-year rotation with its many spring crops. The same situation ranking was observed for yield loss though the differences among situations were smaller (Figure 22.B).

When looking at species richness, the situation ranking was roughly the opposite, except for the sharp drop in species richness in no till. Variations in average bee food were small though significant (Figure 22.F). Bee food offer was overall low. Most noticeable was the increase in bee food in no till and with the initial weed seed bank including the most beneficial species as well as the drop with the seed bank consisting mostly of harmful grass weeds.

Finally, herbicide use intensity was consistent with the simulation plan (Figure 22.D). It was the same in all situations except in organic systems (where it was nil) and no till (which was compensated by increased herbicide use.

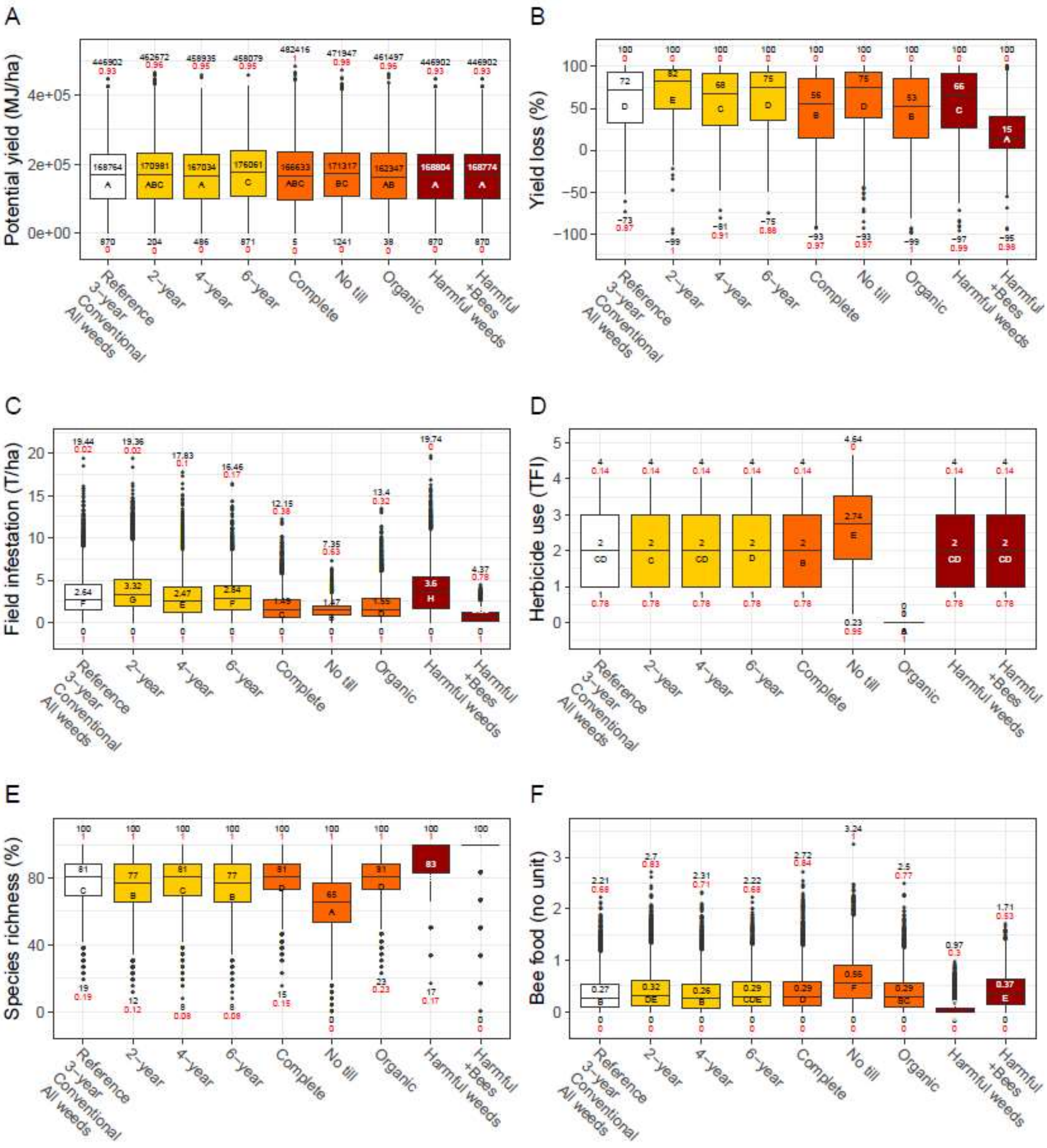

Figure 22. Potential pea yield (from weed-free simulations) and weed (dis)service indicator in years with pea from 400 cropping systems x 12 years x 10 weather repetitions per situation simulated with FLORSYS. Numbers above and below whiskers are minimum and maximum values of each indicator in each situation: in black, untransformed values, in red values rescaled to [0,1] with 0 and 1 respectively worst (lowest yield or biodiversity, highest harmfulness or herbicide use) and best values (the opposite) over all 9 situations. Numbers inside the boxes are median values. Boxes including the same letters show indicators whose means are not significantly different at p=0.05 among what ??? (Nathalie Colbach 2020 [CC BY-NC-ND]).

E. Pea parameters and management techniques driving weed impact

See SupplMatOnlineSectionE.xls

This section comprises the complete results (partial R^2, Variable Importance VIP, probabilities of effects) of the classification and regression trees analysing weed (dis)service indicators as a function of situation, pea parameters, pea management techniques and other-crop management techniques, per pea-variety type (spring vs winter) and analysis scale (years with pea vs average over rotation)

F. CART for weed-impact indicators in different situations

See Colbach et al - SupplMatOnlineSectionF.zip

This section comprises the complete list of decision trees allowing to identify the combinations of pea parameters, pea management techniques and other-crop techniques needed to reach a given objective in terms of weed (dis)service indicators, based on the classification and regression trees analysing weed (dis)service indicators as a function of pea parameters, pea management techniques and other-crop management techniques, per pea-variety type (spring vs winter) and analysis scale (years with pea vs average over rotation). Some specific cases are also included, e.g., without herbicides, without tillage etc.

The zip file comprises two directories:

- AllBranches comprises csv-files called *Feuilles_Indicator_VarietyType_Scale_Systems.csv* with the following variables:
  - idLeaf: Identity of terminal leaf node
  - n = number of individuals in this leaf
  - MeanIndicator = mean indicator value (if single-indicator tree) or mean of mean indicator values (if multi-indicator tree) for the leaf. Indicator values were normed into [0, 1] where 0 was the worst value (e.g., lowest yield, high yield loss, etc) in the data set and 1 the best (highest yield, lowest yield loss etc)
  - A list of rules describing the successive splits (with primary and surrogate variables) in the CART to reach the leaf.
- BestBranches comprises files called
  - *BestBranches_Indicator_VarietyType_Scale_Systems.csv* with the three best branches corresponding to the indicator x variety type x analysis scale … combination indicated in the file name. Branches of multi-indicator trees were ranked based on the average of the constituting indicators.
  - *BestMinBranches_Indicator_VarietyType_Scale_Systems.csv* for multi-indicator trees where the three best branches were chosen as the ones with the highest minimum values of the constituting indicators.

Indicator is one of the following (see details in section A.4):

- BeeFood: weed-based trophic resources for domestic bees,
- EnergyYield: yield in MJ/ha in the presence of weeds,
- FieldInfestation: infestation of cash crops with weed biomass,
- GrainYieldLoss: grain yield loss due to weeds,
- PotentialEnergyYield: yield in MJ/ha in the absence of weeds
- SpeciesRichness: weed species richness,
- P_Integrated: combining actual yield and herbicide use intensity,
- P_Agroecology: combining the previous as well as bee food offer.

VarietyType is either *spring* or *winter* pea. If missing (or *All*), all varieties were included.
Scale is either *AnnualPea* (only years with pea) or *Rotation* (average over all years, including non-pea crops).
Systems can be *WithHerbicides* (with herbicides in pea if *AnnualPea*, in rotation if *Rotation*), *NoHerbicides* (no herbicides), *NoTill* (without tillage before pea or in rotation). If missing (or *All*), all crops and cropping systems were included.

G. Acknowledgements

This project was supported by INRAE, the French National Research Agency (ANR) Project Investissements d'Avenir PeaMUST (adaptation multistress des protéagineux) under the grant number ANR-11-BTBR-0002, the European Union's Horizon 2020 Research and innovation programme under grant agreement N 727217 (ReMIX project), the Casdar RAID project funded by the French Ministry in charge of Agriculture and Food (Ministère de l'Agriculture et de l'Alimentation, avec la contribution financière du compte d'affectation spéciale 'Développement agricole et rural').

H.References

Brisson, N., Launay, M., Mary, B., Beaudoin, N., 2009. Conceptual basis, formalisations and parameterization of the STICS crop model. éditions Quae,

Castel, T., Lecomte, C., Richard, Y., Lejeune-Hénaut, I., Larmure, A., 2017. Frost stress evolution and winter pea ideotype in the context of climate warming at a regional scale. OCL 24, D106, 10.1051/ocl/2017002.

Colbach, N., Busset, H., Roger-Estrade, J., Caneill, J., 2014a. Predictive modelling of weed seed movement in response to superficial tillage tools. Soil & Tillage Research 138, 1-8

Colbach, N., Collard, A., Guyot, S. H. M., Mézière, D., Munier-Jolain, N. M., 2014b. Assessing innovative sowing patterns for integrated weed management with a 3D crop:weed competition model. European Journal of Agronomy 53, 74-89, http://dx.doi.org/10.1016/j.eja.2013.09.019.

Colbach, N., Bockstaller, C., Colas, F., Gibot-Leclerc, S., Moreau, D., Pointurier, O., Villerd, J., 2017. Assessing broomrape risk due to weeds in cropping systems with an indicator based on a simulation model. Ecological Indicators 82, 280–292, dx.doi.org/10.1016/j.ecolind.2017.05.070.

Colbach, N., Moreau, D., Dugué, F., Gardarin, A., Strbik, F., Munier-Jolain, N., 2020. The response of weed and crop species to shading. How to predict their morphology and plasticity from species traits and ecological indexes? European Journal of Agronomy 121, 126158, https://doi.org/10.1016/j.eja.2020.126158.

Gardarin, A., Dürr, C., Colbach, N., 2010. Effects of seed depth and soil structure on the emergence of weeds with contrasted seed traits. Weed Research 50, 91-101

Gardarin, A., Dürr, C., Colbach, N., 2012. Modeling the dynamics and emergence of a multispecies weed seed bank with species traits. Ecological Modelling 240, 123-138, http://dx.doi.org/10.1016/j.ecolmodel.2012.05.004.

Gardarin, A., Coste, F., Wagner, M.-H., Dürr, C., 2016. How do seed and seedling traits influence germination and emergence parameters in crop species? A comparative analysis. Seed Science Research 26, 317-331, https://doi.org/10.1017/s0960258516000210.

Lecomte, C., Giraud, A., Aubert, V., 2003. Testing a predicting model for frost resistance of winter wheat under natural conditions. Agronomie 23, 51-66, 10.1051/agro:2002068.

Mézière, D., Lucas, P., Granger, S., Colbach, N., 2013. Does integrated weed management affect the risk of crop diseases? A simulation case study with a grass weed and a soil-borne cereal disease. European Journal of Agronomy 47, 33-43, http://dx.doi.org/10.1016/j.eja.2013.01.007.

Mézière, D., Petit, S., Granger, S., Biju-Duval, L., Colbach, N., 2015. Developing a set of simulation-based indicators to assess harmfulness and contribution to biodiversity of weed communities in cropping systems. Ecological Indicators 48, 157-170, http://dx.doi.org/10.1016/j.ecolind.2014.07.028.

Munier-Jolain, N. M., Guyot, S. H. M., Colbach, N., 2013. A 3D model for light interception in heterogeneous crop:weed canopies. Model structure and evaluation. Ecological Modelling 250, 101-110, http://dx.doi.org/10.1016/j.ecolmodel.2012.10.023.

Munier-Jolain, N. M., Collard, A., Busset, H., Guyot, S. H. M., Colbach, N., 2014. Investigating and modelling the morphological plasticity of weeds in multi-specific canopies. Field Crops Research 155, 90-98, http://dx.doi.org/10.1016/j.fcr.2013.09.018.

Pointurier, O., Moreau, D., Pagès, L., Caneill, J., Colbach, N., 2021. Individual-based 3D modelling of root systems in heterogeneous plant canopies at the multiannual scale. Case study with a weed dynamics model. Ecological Modelling 440, 109376, https://doi.org/10.1016/j.ecolmodel.2020.109376.

Raveneau, M. P., Coste, F., Moreau-Valancogne, P., Lejeune-Hénaut, I., Dürr, C., 2011. Pea and bean germination and seedling responses to temperature and water potential. Seed Science Research 21, 205-213

Tayeh, N., Aubert, G., Pilet-Nayel, M.-L., Lejeune-Hénaut, I., Warkentin, T. D., Burstin, J., 2015. Genomic Tools in Pea Breeding Programs: Status and Perspectives. Frontiers in Plant Science 6, 10.3389/fpls.2015.01037.

Varela Nicola, F., 2017. Découverte de l’agriculture française, détermination de la température de base de plusieurs variétés de pois et participation à des expérimentations en serre et au champ de l’UMR Agroécologie. AgroSup Dijon-ARFAGRI, Dijon, France.
